# Evolution, structure and function of the putative biosynthetic gene cluster of the fungal secondary metabolite myriocin, a potent inhibitory sphingolipid

**DOI:** 10.1038/s42003-026-10708-9

**Published:** 2026-07-17

**Authors:** Ben Rutter, Michael A. Herrera, Gustavo Perez Ortiz, Claudio Greco, Ben Ashley, Hai Deng, Abigail Hay, Cameron Bedford, Marius Wenzel, Stephen Mondo, Zachary Konkel, Jason C. Slot, Qinxi Ma, Nicolas Helmstetter, Rhys A. Farrer, Dominic J. Campopiano, Alexandra C. Brand

**Affiliations:** 1 School of Medicine, Medical Sciences & Nutrition, University of Aberdeen, Aberdeen, UK; 2 School of Chemistry, University of Edinburgh, Edinburgh, UK; 3 Department of Biosciences, Swansea University, Swansea, UK; 4 Department of Chemistry, School of Natural & Computing Sciences, University of Aberdeen, Aberdeen, UK; 5 Medical Research Council Centre for Medical Mycology at the University of Exeter, Exeter, UK; 6 Centre for Genome-Enabled Biology & Medicine, University of Aberdeen, Aberdeen, UK; 7 School of Biological Sciences, University of Aberdeen, Aberdeen, UK; 8 US Department of Energy Joint Genome Institute, Lawrence Berkeley National Laboratory, Berkeley, CA 94720 USA; 9 Department of Plant Pathology, The Ohio State University, Columbus, USA; 10 Living Systems Institute, University of Exeter, Exeter, UK

**Keywords:** Sphingolipids, Fungal evolution, Expression systems, Natural product synthesis

## Abstract

Myriocin is a fungal secondary metabolite exploited worldwide as a powerful inhibitor of sphingolipid biosynthesis through its structural similarity to sphingosine. We identify the putative myriocin biosynthesis gene cluster (BGC) through de novo sequencing of two producing fungi, *Isaria sinclairii* and *Mycelia sterilia*, yielding genomes of 25.2 Mb and 34.2 Mb encoding 27 and 20 secondary metabolite BGCs, respectively. BGCs #5 in *I. sinclairii* and #18 in *M. sterilia* both shared and expressed the polyketide synthase (PKS) and alpha oxo-amine synthase (AOS) predicted for myriocin biosynthesis, with 74% and 79% sequence similarity, respectively. Analysis of a 2,236-fungal-genome database suggests the pathway originated in the *Sordariomycete* ancestor, presenting in two major clades distinguished by PKS gene orientation. The placement of thermophilic *M. sterilia* suggests myriocin BGC acquisition through horizontal gene transfer, but its origin in *I. sinclairii* is ambiguous. Heterologously-expressed IsMyrA bound aminomalonate, and a protein-protein docking interface was identified between the acyl carrier protein and IsMyrA. A model of PKS domain function, the roles of the PKS and AOS genes and the synteny of the putative myriocin biosynthetic gene cluster across 34 carrier species of ascomycetes is presented.

## Abstract (web summary)

Genome sequencing, expressed gene function and phylogenetic analyses of two myriocin-producing fungi provide insights into to the genetic origins of this natural-product sphingolipid inhibitor and its distribution within the Pezizomycotina.

## Introduction

**Fig. 1:**
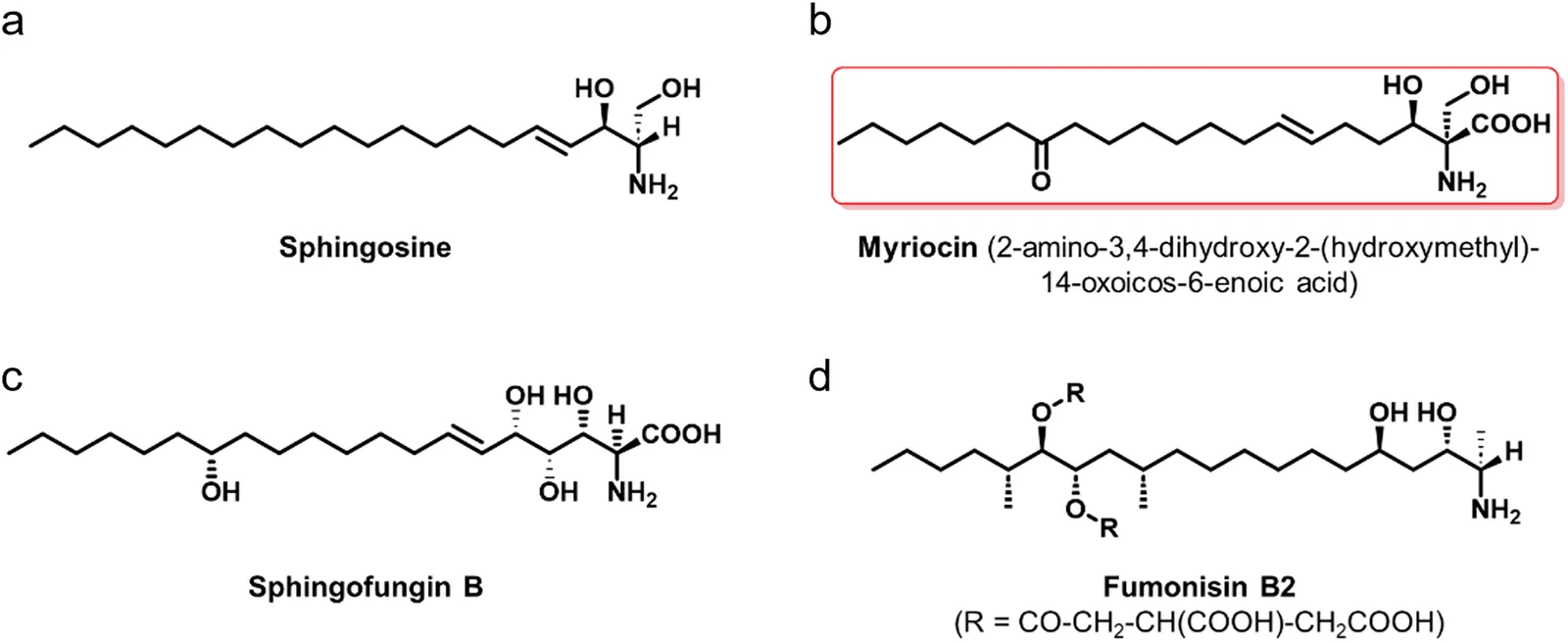
Chemical structures of Sphingosine-Like Mycotoxins (SLMs). **a** Sphingosine; **b** Myriocin (boxed); **c** Sphingofungin B; **d** Fumonisin B2.

Myriocin is a fungal secondary metabolite with a sphingosine-like structure—a long-chain fatty acid with an amino head group (Fig. 1a, b). It is also known as thermozymocidin and ISP-1: ((2*S*, 3*R*, 4*R*)-(*E*)-2-amino-3, 4-dihydroxy-2-hydroxymethyl-14-oxoeicos-6-enoic acid). Myriocin was first identified in independent screens for antifungal activity from natural products from the fungi *Myriococcum albomyces*^1^ (now *Melanocarpus albomyces*), *Mycelia sterilia*^2^ and *Melanconis flavovirens*^3^. A screen for fungal metabolites with immunosuppressant activity identified a compound from *Isaria sinclairii* that inhibited graft rejection in a rat dorsal skin transplant model and exhibited 10–100-fold more potency than cyclosporin A^4^. This compound, ISP-1, was found to be identical to myriocin. By binding an aldimine within the active site of serine palmitoyltransferase (SPT), myriocin inhibits the condensation reaction between serine and Palmitoyl-CoA that generates 3-ketosphinganine. Conserved in yeast and mammals^5^, this is the first committed step in the biosynthesis of complex sphingolipids that are important components of lipid membranes and cellular signalling^6–8^. Myriocin therefore shares a similar mode of action to the sphingofungins (Fig.1c), which are potent SPT inhibitors first isolated from *Aspergillus fumigatus* and *Paecilomyces variotii* in the 1990s^9,10^. Another well-known class of sphingolipid inhibitors are the fumonisins (Fig. 1d), which block the formation of complex sphingolipids by inhibiting Ceramide Synthase (CerS)^11^. Crucially, the potent bioactivity of myriocin, sphingofungins and fumonisins stems from their structural and chemical resemblance to sphingolipids.

Myriocin has been synthesised through a derivatization programme^12^ into a water-soluble form, Fingolimod (‘Gilenya’, Novartis) and was licensed in 2010 as the first oral drug against the relapsing-remitting autoimmune disease, multiple sclerosis^13^. In the human body, Fingolimod is phosphorylated to form Fingolimod-P, which out-competes sphingosine in binding Sphingosine-1-phosphate 1 (S1P_1_) receptors on central memory T-cells (TCM), inducing their internalisation and degradation. This renders TCMs insensitive to the S1P gradient that directs their migration from the lymph node to the bloodstream, from whence they attack myelinated axons in the central nervous system.

Myriocin as an inhibitor of sphingolipid metabolism is also relevant to the study of viral infections, including Hepatitis B^14^, Hepatitis C^15^ and Herpes Simplex Virus (HSV) replication^16^, where there is a potential application for myriocin in the development of anti-tumour (HSV)-based vectors. As an antifungal, myriocin is of interest in the field of medical mycology and shows strong activity against invasive fungal pathogens such as *Candida albicans* (SC5314) (MIC_50_ = 0.12 µg /mL)^17^, *Aspergillus fumigatus* (Af293) (MIC_50_ = 8 µg/mL)^18^ and *Candidozyma auris* (MIC_50_ = ≤ 0.5 µg/mL)^19^.

The primary myriocin-producing fungi have been identified as members of the *Sordariomycetes* class of Ascomycota. Although genome sequences are available for many of these species, the gene cluster responsible for myriocin biosynthesis has not previously been identified. *I. sinclairii* (teleomorph *Ophiocordyceps sinclairii*) is a haploid entomopathogen native to Asia^4^. *I. sinclairii* spores infect insect larvae of the orders *Hymenoptera* and *Lepidoptera*, where growth within the host leads to the death of the insect. *Mycelia sterilia*, which has not been phylogenetically classified, produces three times the amount of myriocin produced by *I. sinclairii*^20^. However, ‘*Mycelia sterilia*’ is a generic name for the artificial taxonomic group of fungi that do not produce spores, instead growing as fluffy colonies thought to propagate through fragmentation. As a result of the phylogenetic analysis undertaken in this study, we can place *M. sterilia* (ATCC20349) in the *Chaetomiaeceae* family of the *Sordariomycetes* class of fungi.

Even though myriocin is recognised as a potent secondary metabolite with valuable research and clinical applications, its biosynthetic pathway has not been elucidated. Here we assembled de novo the genomes of two myriocin-producing fungi, *I. sinclairii* (ATCC24400) and *M. sterilia* (ATCC20349), using combined Illumina and Oxford Nanopore sequencing to identify the biosynthetic gene cluster (BGC) responsible for myriocin production. Comparative genomics revealed that the two fungi shared a single homologous BCG comprising 6–7 core genes, which we designate as *myrA, B, C, D, F, G* and *H*, where *myrB* and *myrA* encode a polyketide synthase and PLP-dependent alpha oxo-amine synthase (AOS), respectively, that are predicted to be required for myriocin biosynthesis. Heterologous expression of *Is*MyrA and protein modelling confirmed preferential binding, in vitro and in silico, of a C_18_ fatty acid backbone to aminomalonate, and not to L-serine, placing myriocin within an emerging functional class of fungal secondary metabolites that inhibit sphingolipid and ceramide biosynthesis. We term these ‘Sphinganine-Like Mycotoxins (SLMs)’ that include fumonisin and phytosphingosine, which also act on the sphingolipid/ceramide biosynthetic pathway. Examination of published fungal genomes revealed that homologs of this putative myriocin BGC are ancestral in two major orders of *Ascomycota – Xylariales* and *Eurotiales* – where each order exhibits a different general arrangement of the BGC. However, placement of *I. sinclairii* within *Ophiocordycipitaceae* (*Hypocreales*) and *M. sterilia* within the *Chaetomiaceae* (*Sordariales*) provided exemplars of the insertion into these clades of other species carrying this BGC, consistent with a broader distribution of this cluster and multiple potential horizontal gene transfer events that have introduced the BGC outside the two main orders.

## Results

### *I. sinclairii* undergoes temperature-dependent morphogenesis, and *M. sterilia* is a constitutively filamentous thermophile

**Fig. 2:**
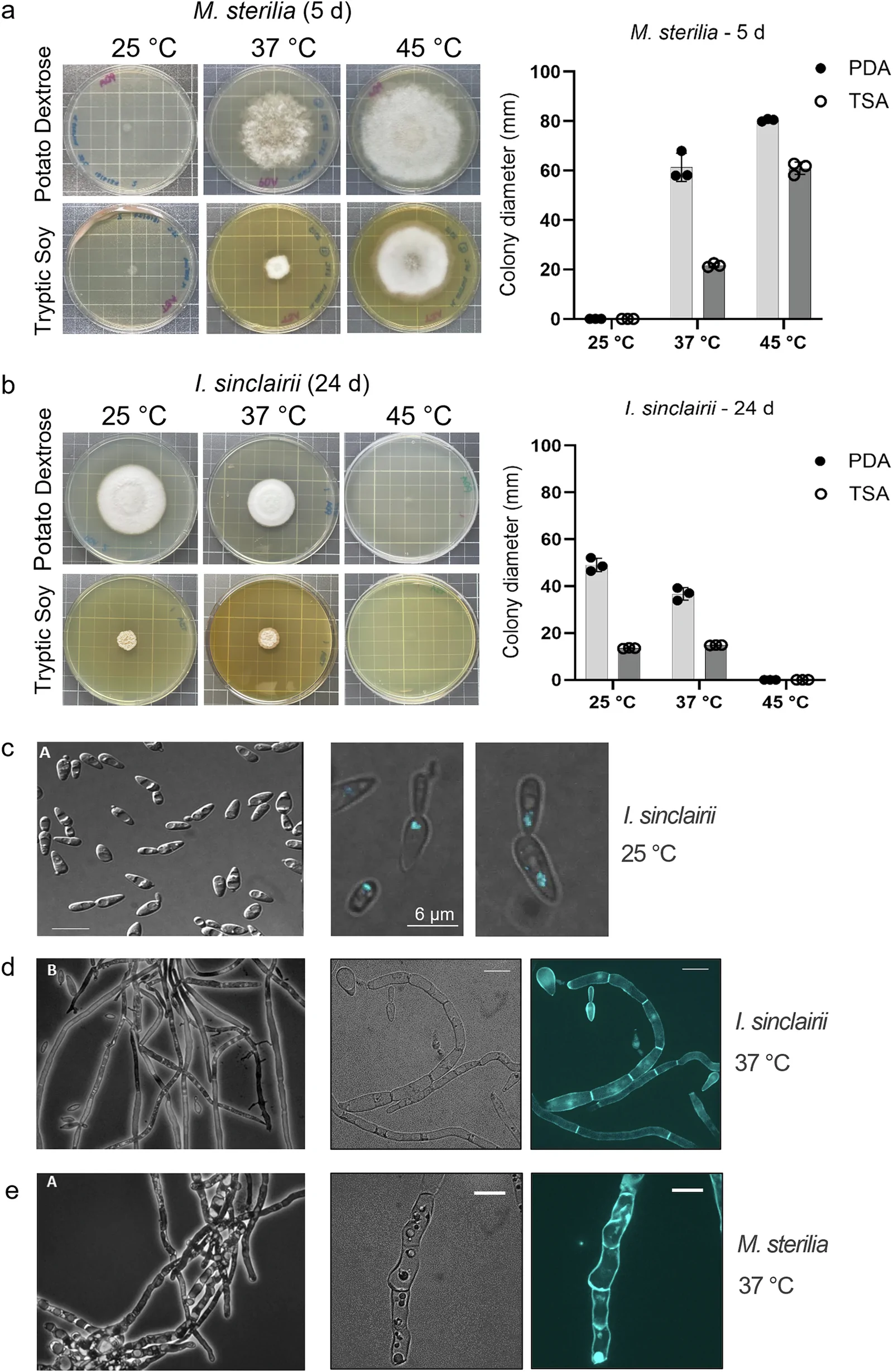
Temperature-dependent dimorphism of *I. sinclairii* and the constitutively filamentous growth of thermophilic *M. sterilia*. The effect of growth medium and temperature on colony growth by **a**. *M. sterilia* at 5d and **b**. *I. sinclairii* at 24 d, on potato dextrose agar (grey bars) or tryptic soy agar (black bars) at 25, 37, or 45 °C. **c** In liquid culture at 25 °C, *I. sinclairii* formed elongated budding yeast cells. Staining with DAPI revealed cells were mononucleate. **d** At 37 °C, *I. sinclairii* formed hyphal filaments. Staining with Calcofluor White showed strong fluorescence at hyphal septa relative to the parallel-sided cell wall. **e** Growth of *M. sterilia* was constitutively filamentous, exhibiting septal-bound compartments of variable width and irregular morphology. Bars = 10 µm.

Beyond their shared ability to produce myriocin, little is known of the biology of *I. sinclairii* or *M. sterilia*. To understand more about their lifestyles, the effect of temperature and growth medium on cell morphology and colony growth were determined. *M. sterilia* did not grow on either solid potato dextrose agar (PDA) or tryptic soy agar (TSA) at 25 °C, but colony diameter increased with temperature at 37 °C and 45 °C, reaching the plate periphery within 5 d (Fig. 2a). The faster growth rate at 45 °C compared to 37 °C classifies *M. sterilia* as a thermophilic fungus^21^. In contrast, the growth of *I. sinclairii* negatively correlated with temperature on solid media, with maximal growth at 25 °C and none at 45 °C (Fig. 2b). Although both fungi grew faster on potato dextrose than on tryptic soy agar, the growth of *I. sinclairii* on both media was slow compared to *M. sterilia* and colonies did not reach the plate periphery in 24 d. The two fungi also exhibited differential susceptibility to antifungal compounds: *I. sinclairii* was sensitive to the antifungal drugs, fluconazole and hygromycin B, but not to nourseothricin, while *M. sterilia* was sensitive to hygromycin B and nourseothricin, but not to fluconazole (Figure S1). These results point to hygromycin B as a shared potential selectable marker for genetic modification approaches in these two fungi, although *M. sterilia* was up to 10-fold more sensitive to this drug than *I. sinclairii*.

In liquid fermentation medium at 25 °C, *I. sinclairii* grew as a mononucleate elongated yeast (Fig. 2c). No growth for *M. sterilia* was observed at this temperature, as seen on solid medium. At 37 °C, both fungi grew as hyphal filaments (Fig. 2d, e), indicating that *I. sinclairii* undergoes a yeast-to-hyphal switch at the higher temperature. *I. sinclairii* hyphae were parallel-sided, and septa stained more strongly than the hyphal cell wall with Calcofluor White, suggesting that septa have a relatively high chitin content in this fungus. Under all conditions tested, *M. sterilia* grew only in the hyphal form, which formed hyphal aggregates in liquid media. Hyphae were non-parallel-sided with expanded regions in the walls adjacent to septa. Both fungi therefore grew as strings of non-separated cell compartments, as opposed to undergoing the coenocytic cytoplasmic streaming observed in many filamentous fungi. Together, these findings indicate that, although *M. sterilia* and *I. sinclairii* both maintain a putative myriocin BGC, their lifestyles are otherwise divergent.

### Myriocin content in *I. sinclairii* and *M. sterilia* cells and supernatant

**Fig. 3:**
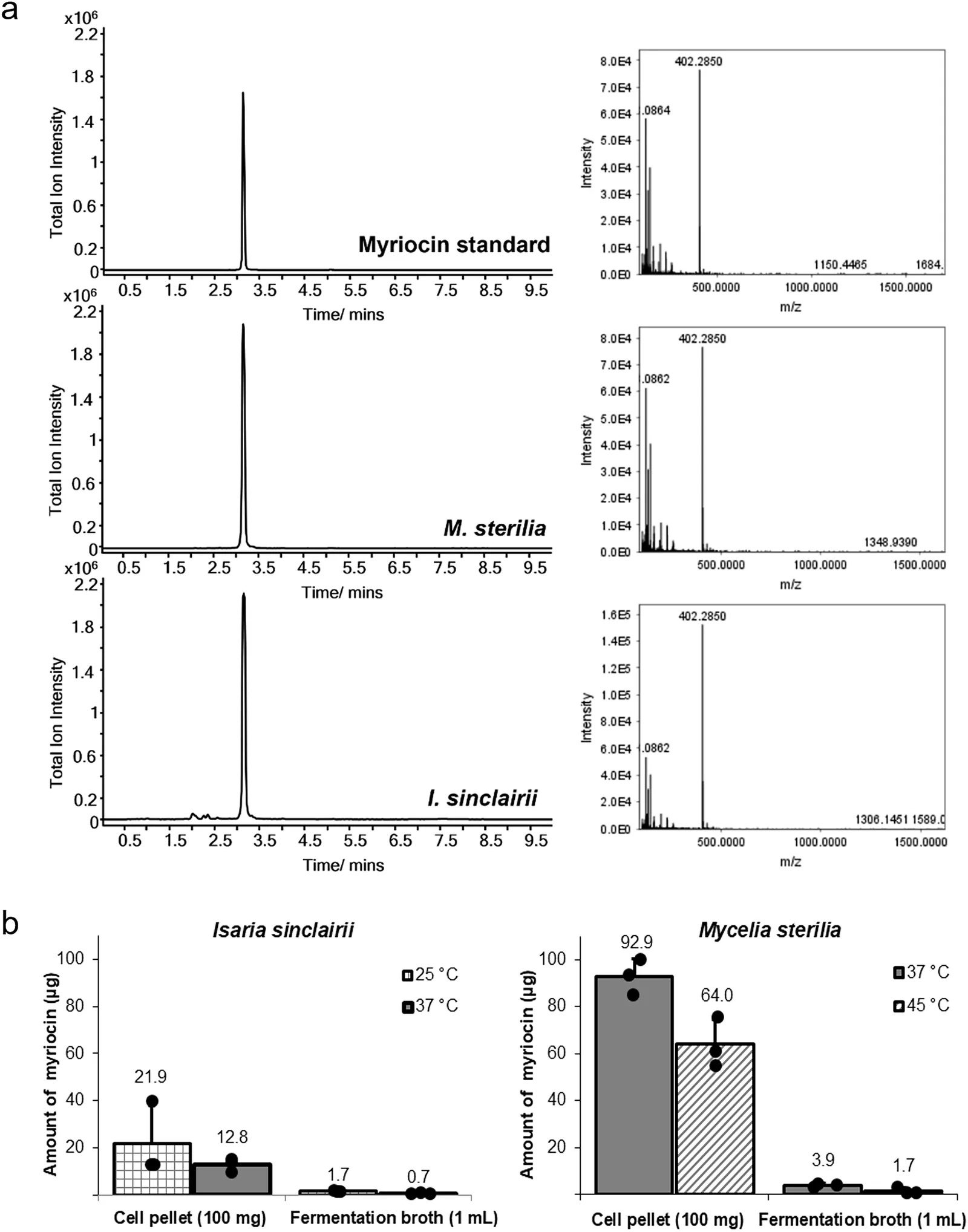
Detection and quantification of myriocin in fungal cell pellets and supernatant. **a** Detection of myriocin using UHPLC-HRMS analysis: Extracted ion chromatograms (402.285) in positive mode of myriocin chemical standard, *M. sterilia* and *I. sinclairii* organic extracts with their relative mass spectra, with linked axis. LCMS method: 30–98% (MeCN:H_2_O), 10 min. **b** Quantification of myriocin extracted from the cell pellet or fermentation broth by *I. sinclairii* at 25 °C and 37 °C (left) or *M. sterilia* at 37 °C and 45 °C (right) at 24 d. The mean weight of myriocin (µg per 100 mg dried cells or per 1 mL culture medium) is reported above each column (*n* = 3 independent samples, bars = SD).

The known structure of myriocin (Fig. 1) and availability of a commercial standard allowed us to compare the production and secretion of myriocin by *M. sterilia* and *I. sinclairii* at their two permissive growth temperatures. *I. sinclairii* has previously been reported to secrete myriocin at detectable levels after two-stage culture for 10d at 30 °C in fermentation medium^4^. For a direct comparison, the two fungi were incubated for 24d in fermentation medium at 25 °C and 37 °C for *I. sinclairii* or 37 °C and 45 °C for *M. sterilia*, before extraction of 100 mg of desiccated cell pellets and 1 mL fermentation broth with ethyl acetate, followed by liquid chromatography-high resolution mass spectroscopic (UHPLC-HRMS) analysis (Supplementary Data Set 1). A metabolite eluting at 3.1 min with the mass-to-charge ratio (*m/z*) of 402.285 [M + H]^+^ was identified in the cell pellet and fermentation broth of both fungi (calcd: 402.277, Δ = 0.46 ppm), corresponding to myriocin (C_21_H_39_O_6_N) (Fig. 3a). The metabolite had the same retention time and mass spectrum as that of the myriocin chemical standard (Fig. 3a). Both fungi also produced a minor metabolite eluting at 1.9 min, with an *m/z* of 428.228, which matches a previously-reported myriocin derivative (C_21_H_41_O_7_N^+^, calcd: 418.28, Δ = −0.54 ppm) (Figure. S2a, b)^20^, but no standard was available to confirm the structure. Myriocin production was quantified in both strains against a chemical standard and by integrating the area of the peak in the extracted ion chromatogram. In *I. sinclairii*, myriocin production was ~2-fold higher per 100 µg biomass and per 1 mL culture medium at 25 °C compared to 37 °C (Fig. 3b). Surprisingly, in *M. sterilia*, myriocin production was nearly 1.5-fold higher at 37 °C than at 45 °C, despite its faster growth rate on agar plates at the higher temperature. A comparison of the mean cell biomass produced by the two fungi in 50 mL fermentation medium grown at 37 °C for 24d showed that *I. sinclairii* generated significantly more biomass (0.33 ± 0.04 mg) than *M. sterilia* (0.25 ± 0.05 mg) (*p* ≤ 0.0285, *n* = 5 biologically independent samples) but yielded ~5-fold less myriocin per 50 mL culture. Overall, *M. sterilia* produced more than 7-fold the amount of myriocin per 100 µg cell pellet than *I. sinclairii*, and secreted 5.5-fold more myriocin per 1 mL of supernatant. Retention of such high levels of myriocin in the cell pellets relative to secreted amounts suggests these fungi have an effective resistance mechanism to the myriocin they produce.

### De novo sequencing and genome assembly for *I. sinclairii* and *M. sterilia*

To identify the myriocin BGC, the genomes of *I. sinclairii* and *M. sterilia* were sequenced using the Illumina MiSeq and the Oxford NanoPore MinION sequencing platforms. The k-mer spectra of the Illumina data indicated a single well-defined coverage peak for both species and suggested a haploid genome size of c. 20 Mbp for *I. sinclairii* and

c. 30 Mbp for *M. sterilia*, with little evidence for diploidy in either species (heterozygosity was estimated as 0.37 and 1.71%, respectively). However, read coverage also suggested an increase in sequence redundancy in the *M. sterilia* assembly compared to *I. sinclairii* (7.32% vs 4.04% redundant read alignments; 1.37% vs 0.65% redundant assembly length), so a small degree of heterozygosity in a diploid *M. sterilia* genome cannot be ruled out. High-quality genome assemblies were achieved with total lengths of 25.2 Mb for *I. sinclairii* and 34.2 Mb for *M. sterilia* (Figure. S3a), which fall within the range for Ascomycota (up to 63 Mbp)^22^. The assemblies were highly contiguous (*N*_*50*_ = 1.4 Mb and 817 kb for *I. sinclairii* and *M. sterilia*, respectively) and had BUSCO completeness scores of 98.6% and 99.3%, respectively. In both genome assemblies, raw read coverage per contig clearly identified a single high-coverage contig derived from the mitochondrial genome (*I. sinclairii*: 158,893 bp [contig Segkk24, NCBI accession RXPI01000025]; *M. sterilia*: 180,440 bp [contig Segkk118, NCBI accession RXPH01000119]). We identified 8,167 and 8,706 protein-coding genes, respectively. Analysis by antiSMASH fungal (v 7.1.0) identified 27 and 20 putative secondary metabolite BGCs for *I. sinclairii* and *M. sterilia*, respectively (Figure S3b, c). The *I. sinclairii* genome appeared to contain a broader range of secondary metabolite BGCs than *M. sterilia*, with putative representatives of 7 of 8 classes, including 3 RiPP (ribosomally synthesized and post-translationally modified peptides (reviewed in ref. ^23^) BGCs, compared to 5 classes and no RiPP BGCs in the latter. However, RiPP predictions in fungal genomes can include false positives due to misclassification of non-RiPP short ORFs or tailoring enzymes^24–26^. Similarly, ‘indole’ BGC predictions may lack a true indole moiety and instead represent other alkaloid variants. These putative BGCs were therefore not subject to further analysis in this study.

### Myriocin biosynthetic gene cluster prediction, identification, synteny and expression

**Fig. 4:**
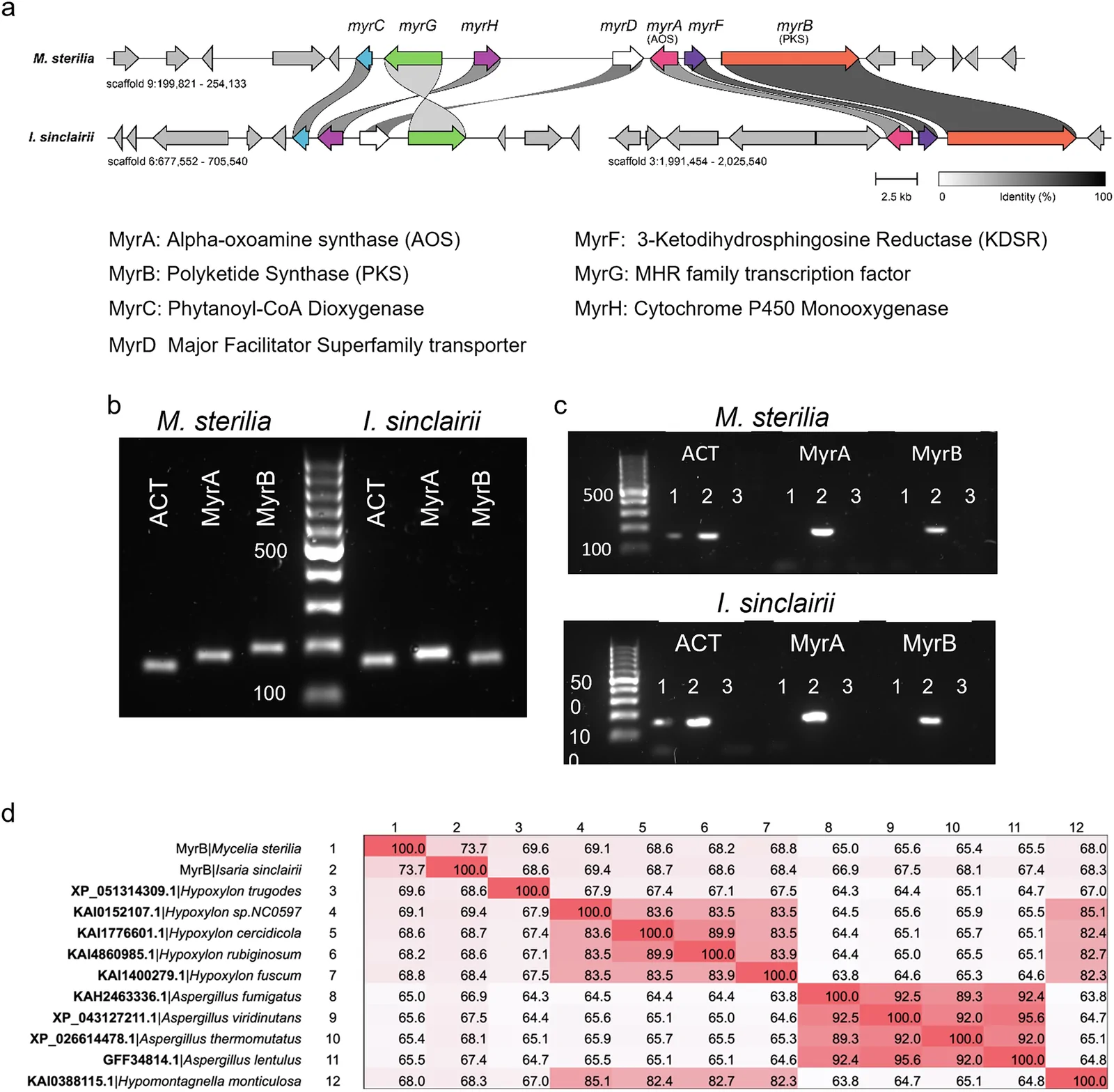
Synteny in *I. sinclairii* and *M. sterilia* genomes of a shared biosynthetic gene cluster (BGC) and expression of genes encoding the iPKS and AOS required for myriocin biosynthesis. **a** Synteny plot for the 7 core genes (listed) shared between *I. sinclairii* and *M. sterilia*; Grey genes lack additional homology between the strains so indicate the flanking regions of the myriocin BGC. **b** PCR products from genomic DNA of actin, *myrA* and *myrB*, shown against a 100 bp DNA ladder. For uncropped gels, see Figure S13. **c** RT-PCR products from *M. sterilia* and *I. sinclairii* of actin, *myrA* and *myrB*, shown against a 100 bp DNA ladder (1 = Day 10, 2 = Day 20, 3 = No nucleic acid control); **d** Percent identity matrix of the highest sequence homologs of *Is*MyrB and *Ms*MyrB (iPKSs) identified using a BLASTp search of fungal genomes. NCBI accession numbers are highlighted in bold. Heat map range: Strong red = 100% identity, white = > 64% identity.

The structure of myriocin suggests that it is derived from an amino acid head-group and a C_18_ fatty acid-like precursor (Fig. 1). Long-chain fatty acids in secondary metabolites are assembled by megasynthases that are usually either a fatty acid synthase (FAS) or a polyketide synthase (PKS). The fatty acid intermediate produced by the megasynthase is then combined with an amino acid by a pyridoxal 5’-phosphate-dependent (PLP-dependent) alpha-oxo-amine synthase (AOS)-like enzyme, as observed in other fungi (see section on AOS below). As genes encoding the enzymes involved in secondary metabolite pathways tend to cluster within the genome, we searched for coding sequences in the genomes of both fungi for possible PKS and AOS genes that were paired within a BGC. The search revealed that both fungi contained 9 BGC clusters that encoded a putative PKS gene. Within these 9, only one cluster in each fungus contained both a PKS and an AOS gene – Cluster 5 in *I. sinclairii* and Cluster 18 in *M. sterilia* (Figure S4a). Closer inspection showed that, within these PKS-AOS gene pairings, one gene encodes a highly reducing iterative type I polyketide synthase (iPKS) of 2500-2513 aa (73.5% identity, 99% coverage), while the other encodes a predicted malonyl-CoA selective acyltransferase (AOS/AT) of 480 aa (78.8% identity). These genes were designated as *myrB* and *myrA*, respectively. Clusters 5 and 18 also contained 5 further genes with high similarity and synteny (Fig. 4a), and were assigned as *myrC, myrD, myrF, myrG, myrH* (see below). Identification in these two myriocin-producing species of a single shared gene cluster that uniquely encodes the predicted PKS-AOS gene pair, along with 5 further shared genes, strongly suggested that they function in myriocin biosynthesis. To test this further, expression of the key *myrB* and *myrA* genes was examined by RT-PCR at two time-points (Days 10 and 20) in cells grown under conditions that induce myriocin biosynthesis, using actin expression as a positive control. While actin was expressed at both time points, no signal was seen for either *myrA* or *myrB* at Day 10 (Fig. 4b, c). However, a strong signal was observed at Day 20 for both genes. Therefore, *myrA* and *myrB* exhibit the same expression profile in culture conditions coincident with myriocin biosynthesis, providing further evidence that these two genes function as predicted within the myriocin BGC.

### Comparative and structural analysis of myriocin biosynthesis genes, *myrB, myrA and myrF*

We undertook comparative and structural analyses of three key enzymes from the identified clusters that are predicted to be involved in the first steps of myriocin biosynthesis, the iPKS, the AOS and the ketodihydrosphingosine reductase (KDSR).

### The myriocin iterative polyketide synthase (iPKS, MyrB*)*

**Fig. 5:**
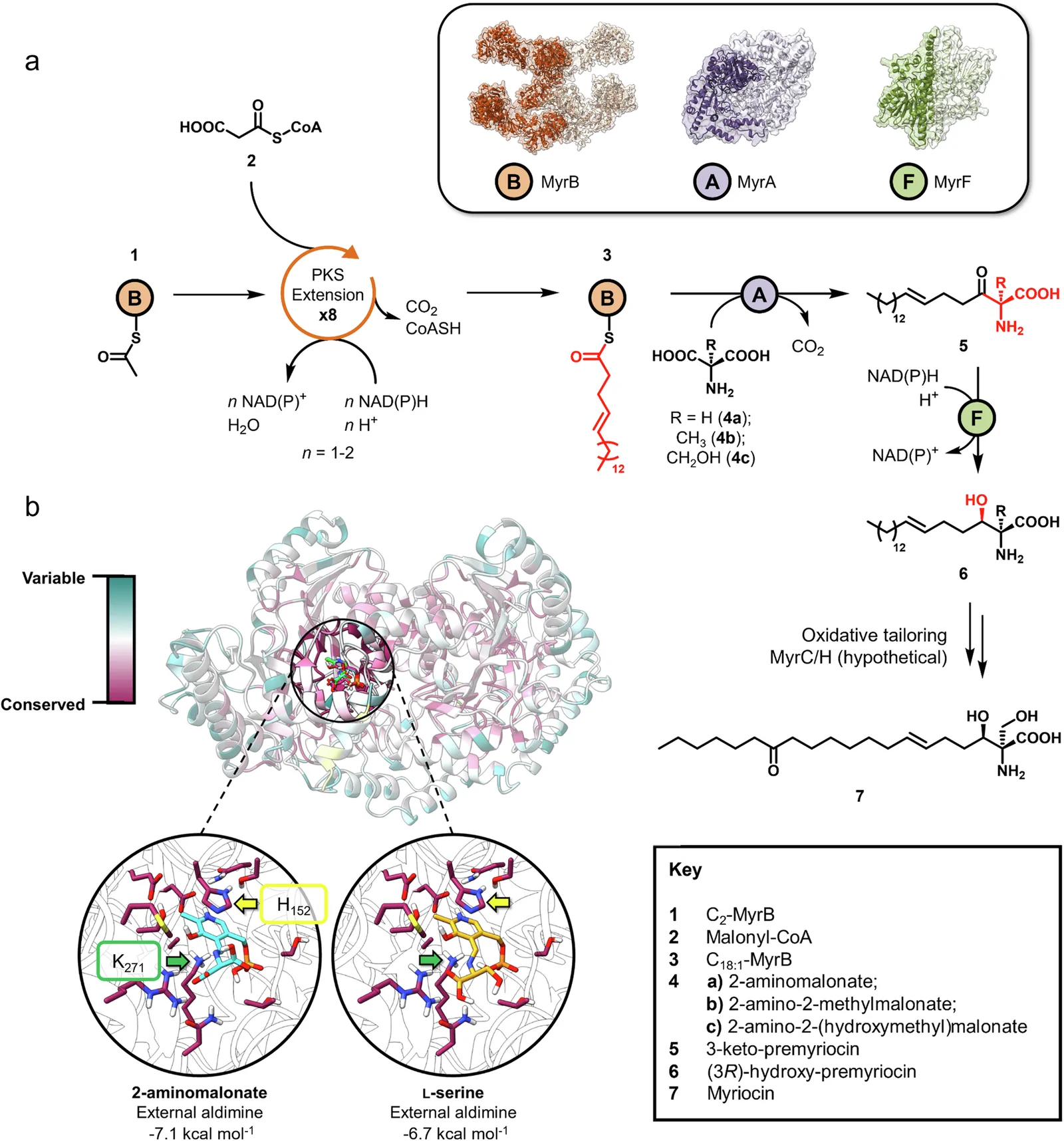
Myriocin structure and proposed biosynthetic pathway. **a** Hypothetical biosynthetic cascade towards myriocin via MyrB, MyrA and MyrF. The C_18:1_ scaffold is produced by the MyrB PKS and released by the MyrA AOS via condensation with 2-aminomalonate (or derivative). Following ketoreduction by the MyrF KDSR, the final molecule is  generated by oxidative tailoring, likely by MyrH and MyrC. **b** Substrate binding to MyrA, the α-oxo-amine synthase (AOS) involved in the condensation of an amino headgroup with a fatty acid chain. Molecular docking experiments using Autodock Vina. 2-aminomalonate and l-serine were docked as PLP external aldimines. Binding affinities (kcal mol^−1^) using the Vina force field are shown. Residues are coloured by conservation; maroon residues are highly conserved, whereas teal residues are mutable. Green and yellow arrows show the positions of the catalytic K_271_ and H_152_, respectively.

The predicted MyrB iPKSs from both fungi share high sequence identity and domain organisation with ‘hallmark’ fungal synthases that remain to be fully characterised but are known to produce a variety of polyketide natural products^27^. The iPKSs with highest sequence identities to *Is*MyrB and *Ms*MyrB were found in *Aspergillus* species (65–66%, ≥99% coverage), *Hypoxylon* species (68–70%, ≥99% coverage) and *Hypomontagnella monticulosa* (≥68%, 99% coverage) (Fig. 4d). The predicted *Is*MyrB and *Ms*MyrB iPKSs also share domain-level homology with the well-characterised iPKSs from *Aspergillus terreus*, LovB (28–29%, 83% coverage), which produces the nonaketide backbone of the cholesterol-lowering blockbuster drug, lovastatin, and where the cryo-EM structure revealed the domain architecture of this megasynthase (PDB: 7CPX)^28^. Similarly, the *A. terreus* LovF iPKS (33–34%, 97% coverage), which catalyses the formation of the key early diketide intermediate that is processed by LovB^29^, displays the same eight-domain architecture as the predicted MyrB iPKSs. We therefore predicted that the iPKSs identified in *I. sinclairii* and *M. sterilia* are responsible for the C_18:1_ carbon scaffold of the myriocin precursor, formed by the iterative Claisen-like condensation of one acetyl-CoA starter unit with eight malonyl-CoA extender units (Fig. 5a). A similar PKS enzyme, SphB, was recently identified in the biosynthetic pathway of sphingofungin, an inhibitor of sphingolipid biosynthesis expressed by *Aspergillus fumigatus*^30^. In accordance with this nomenclature, the two iPKSs we identified are hereby annotated as putative pre-myriocin synthases, *Is*MyrB and *Ms*MyrB, from *I. sinclairii* and *M. sterilia*, respectively.

Protein modelling shows that the MyrBs exhibit canonical fatty acid synthase (FAS)- like and highly-reducing PKS-like architectural features^31,32^, including a predicted homomeric interface comprising the β-ketoacyl synthase (KS), dehydratase (DH) and enoyl reductase (ER) domains (Figure S5a, b). Of note, both MyrBs possess an *S*-adenosylmethionine-dependent methyltransferase (SAM-MT) domain bearing key active site mutations, including a T → R mutation situated in the highly conserved *S*-adenosylmethionine binding pocket (R_1403_ in *Is*MyrB and R_1424_ in *Ms*MyrB (Figure S6a, b)^33–36^. The occlusion of this binding site by a bulky, charged sidechain likely inactivates the MyrB SAM-MTs and may explain the lack of methyl branching in myriocin; these domains may instead serve a solely architectural role in the iPKSs.

### The myriocin AOS (MyrA)

As *myrB* encodes no identifiable chain-terminating domain such as a thioesterase or a thioester reductase, we reasoned that the release of the polyketide product is likely to be mediated by a *trans*-acting enzyme. It is known that the release of the dimethyl-branched C_18_ fumonisin precursor from the iPKS in *F. verticillioides*, Fum1, is facilitated by the AOS, Fum8^37,38^, which catalyses the PLP-dependent condensation of the precursor with L-alanine. Recently, Bissell et al. reported a similar AOS-dependent release mechanism in the producers of another SLM, sphingofungin, in which *A. fumigatus* and *Paecilomyces variotii* utilise 2-aminomalonate (rather than L-alanine or L-serine) to yield the sphingofungin precursor, 3-keto-presphingofungin^30^. The unusual AOS was named SphA. We therefore hypothesised that the head group of the myriocin molecule may originate via a similar AOS-mediated condensation between the C_18:1_ polyketide and an amino substrate. The genes encoding AOS homologues in *I. sinclairii* and *M. sterilia* BGCs are hereafter named *IsmyrA* and *MsmyrA*, respectively.

While the two *myrA* sequences are highly similar to each other, they bear little relation to the AOS involved in fumonisin biosynthesis, Fum8 (< 20% sequence identity). In contrast, *IsmyrA* and *MsmyrA* share >60% sequence identity with *sphA* from *A. fumigatus* (*AfsphA*) and *P. variotii* (*PvsphA*). Additional close homologues were identified in *Penicillium polonicum*, *Monosporascus sp. CRB9-2* and *Neonectria ditissima*. Structurally, MyrA is predicted to resemble several 8-amino-7-oxononanoate synthases (BioF, 1BS0 and 1DJ9) and serine palmitoyltransferases (SPT, PDB: 2JG2 and PDB: 2W8J) whose structures are deposited in the Protein Data Bank (PDB) (pLDDT ≥ 94.0, pTM = 0.93, Figure S7a,b). Using these structural models, l-serine and 2-aminomalonate were docked into the active site as PLP-bound intermediates (Fig. 5b), enabling the identification of the key catalytic lysine (K_271_ in *Is*MyrA and *Ms*MyrA) and conserved pocket residues (including N_63_, H_152_, M_209_, A_231_, D_237_ and R_413_). Given its demonstrated utilisation by *Paecilomyces variotii* SphA, we also considered 2-amino-2-(hydoxymethyl)malonate as a potential substrate for MyrA, which would yield the hydroxymethyl moiety of the myriocin headgroup directly. However, since neither the bioavailability nor biosynthesis of this molecule is known for *I. sinclairii* and *M. sterilia*, this was not investigated further.

Furthermore, a plausible protein-protein docking interface was identified between the MyrB acyl carrier protein (ACP) and MyrA from both species (pTM = 0.88), with the catalytic serine of the ACP positioned directly above the substrate tunnel leading to the catalytic K_271_ of MyrA (approximately 3.4–3.8 nm away, Figure S8a). The overall orientation of the protomers compares favourably (albeit not identically) to the recently solved *E. coli* BioF/crypto-ACP crosslink by Chen et al (PDB: 8DLE)^39^. Furthermore, the AOS-ACP docking interface shows electrostatic complementarity between the electropositive surface of MyrA and the acidic

MyrB ACPs, which may underpin the mutual recognition between these two proteins as observed in other ACP-dependent biosynthetic cascades (Figure S8b, c)^40,41^.

### The myriocin KDSR-like enzyme (MyrF)

Like sphingosine (and sphingosine analogues), myriocin features an (*R*)-hydroxy substituent at the C_3_-position. Therefore, the 3-keto-premyriocin must be reduced by a 3-ketodihydrosphingosine reductase (KDSR)-like enzyme to generate (3 *R*)-hydroxy-premyriocin. The KDSR candidates were identified in both *I. sinclairii* and *M. sterilia* BGCs and are hereafter named *Is*MyrF and *Ms*MyrF, respectively. As with *myrB* and *myrA*, *IsmyrF* and *MsmyrF* share high sequence identity with each other (72.3%, 99% coverage) and with homologues from other ascomycetous fungi (including *Aspergillus*, *Hypoxylon* and *Penicillium* species). The MyrFs were modelled as homodimers (pLDDT ≥ 93.9, pTM ≥ 0.95, Figure S9a,b) and feature Rossmann folds ubiquitous across the NAD(P)H-binding short-chain dehydrogenase/reductase (SDR) oxidoreductase family. A 600-650 A^3^ binding pocket was identified by topological analysis of the MyrFs, each encompassing the glycine-rich nucleotide binding motif TGxxGxxG (T_15_GGSGGLG_22_ in *Is*MyrF and T_13_GGSGGLG_20_ in *Ms*MyrF); NADP^+^ was docked in these binding pockets in a productive conformation, comparable to homologous SDR crystal structures, including the recently-solved KDSR from *Cryptococcus neoformans* (PDB: 8JAT, 6D9Y, 3P19, Figure S10a, b)^42,43^.

Taken together, our analysis suggests that myriocin production follows a similar biosynthetic logic to sphingofungin and fumonisin (Fig. 5). In a hypothetical biosynthetic cascade, the monounsaturated C_18:1_ polyketide scaffold is first synthesised by MyrB and released by MyrA via a Claisen-like condensation with 2-aminomalonate (or a derivative thereof), yielding 3-keto-premyriocin. The subsequent ketoreduction is performed by MyrF to generate (3 *R*)-hydroxy-premyriocin. Additional tailoring reactions are required to introduce the (4 *R*)-hydroxy and 14-keto functional groups. As oxidation at these positions cannot be achieved by a PKS, these modifications are most likely catalysed by a pair of dedicated tailoring enzymes. By homology, we suggest that the cytochrome P450 monooxygenase, MyrH, and the phytanoyl-CoA dioxygenase homolog, MyrC, may provide this oxidative tailoring. Overall, we hypothesise that the combination of iPKS, AOS and KDSR-like biocatalysts acts as a fingerprint for the biosynthesis of sphingosine analogues across many fungal species.

### MyrA heterologous expression and substrate binding

**Fig. 6:**
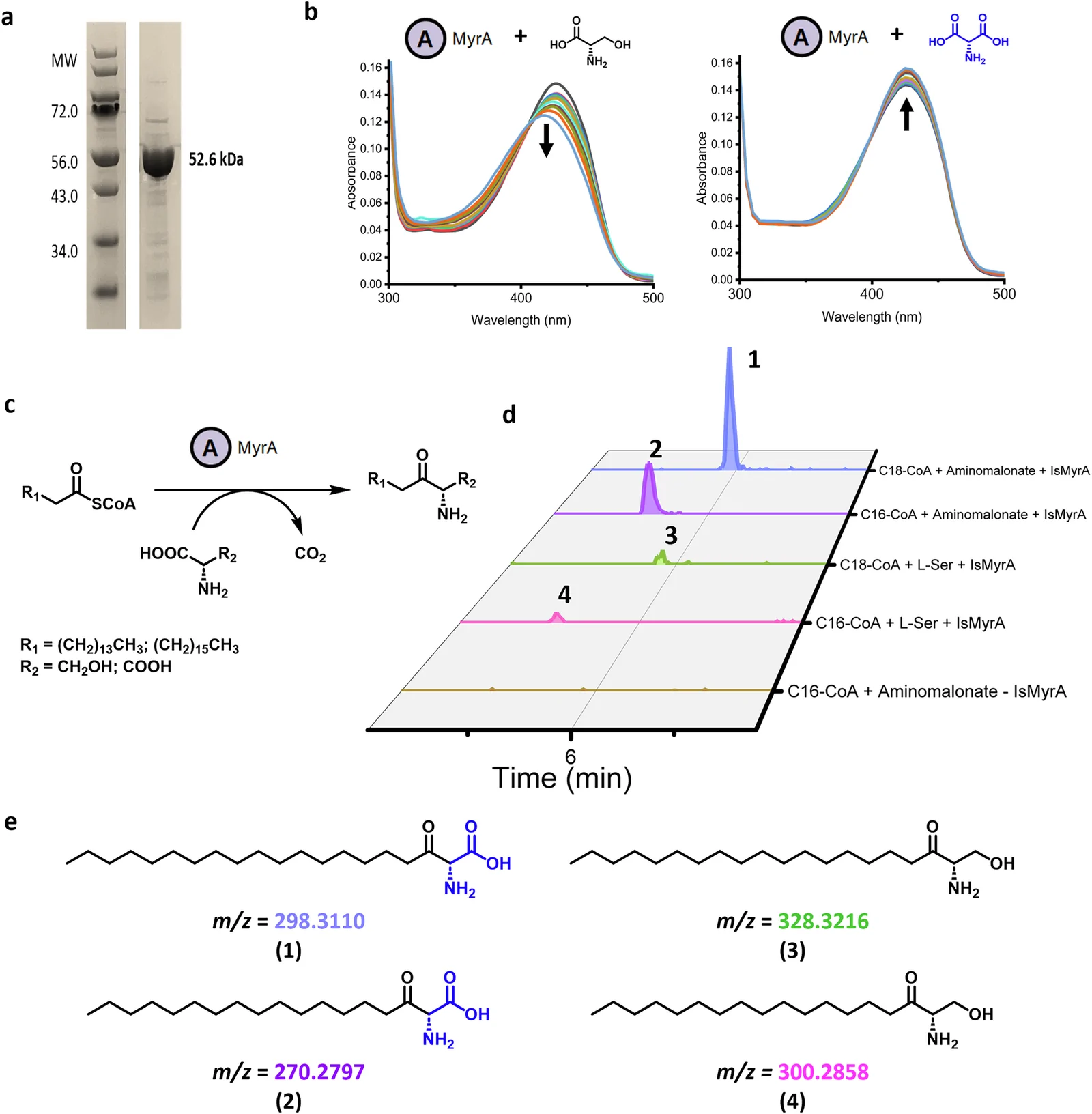
Aminomalonate is the preferred substrate of *Is*MyrA in vitro. **a** SDS-PAGE gel of purified recombinant *Is*MyrA. **b**. UV–vis spectrum of 40 μM *Is*MyrA in increasing concentrations of L-serine (left) and aminomalonate (right). For an uncropped gel image, see Figure S13. **c** Reaction of *Is*MyrA with C_16_ and C_18_ acyl-CoA thioester substrates to sphingosine products with L-Ser and aminomalonate. **d** Extracted ion chromatogram (EIC) overlays of the reaction of IsMyrA with acyl-CoAs and amino acids. **e** The corresponding *m/z* = [M + H^+^] ion observed for each product shown in (**d**) with its molecular formula.

To interrogate the functionality of the myriocin AOS enzyme, the N- and C- termini of the *Is*MyrA protein coding sequence were identified through analysis of published transcriptomics studies previously undertaken in fungi with an identified myriocin BGC homologue (Figure S11a and see below). The *Is*MyrA protein carrying a C-terminal His-tag was synthesised and expressed in *Escherichia coli*. The recombinant *Is*MyrA protein was soluble and was purified by affinity and size-exclusion chromatography (Fig. 6a and S11b–d). *Is*MyrA displayed the characteristic UV–VIS spectrum associated with a PLP-dependent enzyme with a maximum absorbance at 420 nm (Fig. 6b). The ability of the enzyme to generate the PLP:external aldimine form by binding L-serine was measured by the addition of increasing concentrations of the amino acid (0–80 mM). Addition resulted in increased absorbance at 420 nm suggesting *Is*MyrA bound this amino acid. By analogy with SphA, titration of aminomalonate with *Is*MyrA led to a small shift of a few nm to a lower wavelength (410 nm) and a decrease in the intensity of the signal (Fig. 6b). This is consistent with the prediction that *Is*MyrA binds this unusual amino acid^30^. The predicted product of the condensation of the *Is*MyrB polyketide intermediate with an amino acid substrate by the AOS, *Is*MyrA, is 3-keto-premyriocin^30^ (Fig. 5a). In the absence of recombinant acylated *Is*MyrB, acyl-CoA thiol esters were used as substrates in an enzymatic assay to determine if *Is*MyrA was catalytically active. The substrates with C_16_ and C_18_ acyl chains were used as they are closer in length to the predicted natural substrate. Prompted by the work of Bissell et al., both aminomalonate and L-serine were tested as amino acid substrates for the decarboxylative Claisen condensation (Fig. 6c)^30^.

Using an established DTNB assay that monitors the release of CoASH from the acyl-CoA substrate (Figure. S11e)^44^, the optimal amino acid and acyl-CoA substrate combination was aminomalonate, where C_18_-CoA gave the highest activity (Figure. S11f). To further support the observed activity, Liquid-Chromatography-Mass Spectroscopy (LCMS) was used to identify the products formed. The two expected decarboxylated products (Fig. 6d and Figure S11g) were observed, with the greatest product formation detected for a key C_20_ myriocin amino-ketone intermediate that contains a carboxylated head-group (Fig. 6e). Unlike SphA from the biosynthesis of sphingofungin, which only accepts aminomalonate, *Is*MyrA also catalysed the reaction using L-serine, since we also detected the C_20_ product when this amino acid was used (Fig. 6d). However, based on the increased product generated when aminomalonate was used as the substrate, we predict this to be the most suitable substrate for *Is*MyrA for production of a 3-keto-premyriocin intermediate by a decarboxylative Claisen condensation reaction. These experimental data are also supported by docking studies where the key PLP:amino acid external aldimine intermediates are shown to bind in a highly conserved active site (Fig. 5b). Given the high sequence identity (78.8%, Figure S4a) between *Is*MyrA and *Ms*MyrA, it is likely that they use the same substrate combination.

### Distribution and evolution of homologs of myriocin/sphingolipid BGCs across *Pezizomycotina*

**Fig. 7:**
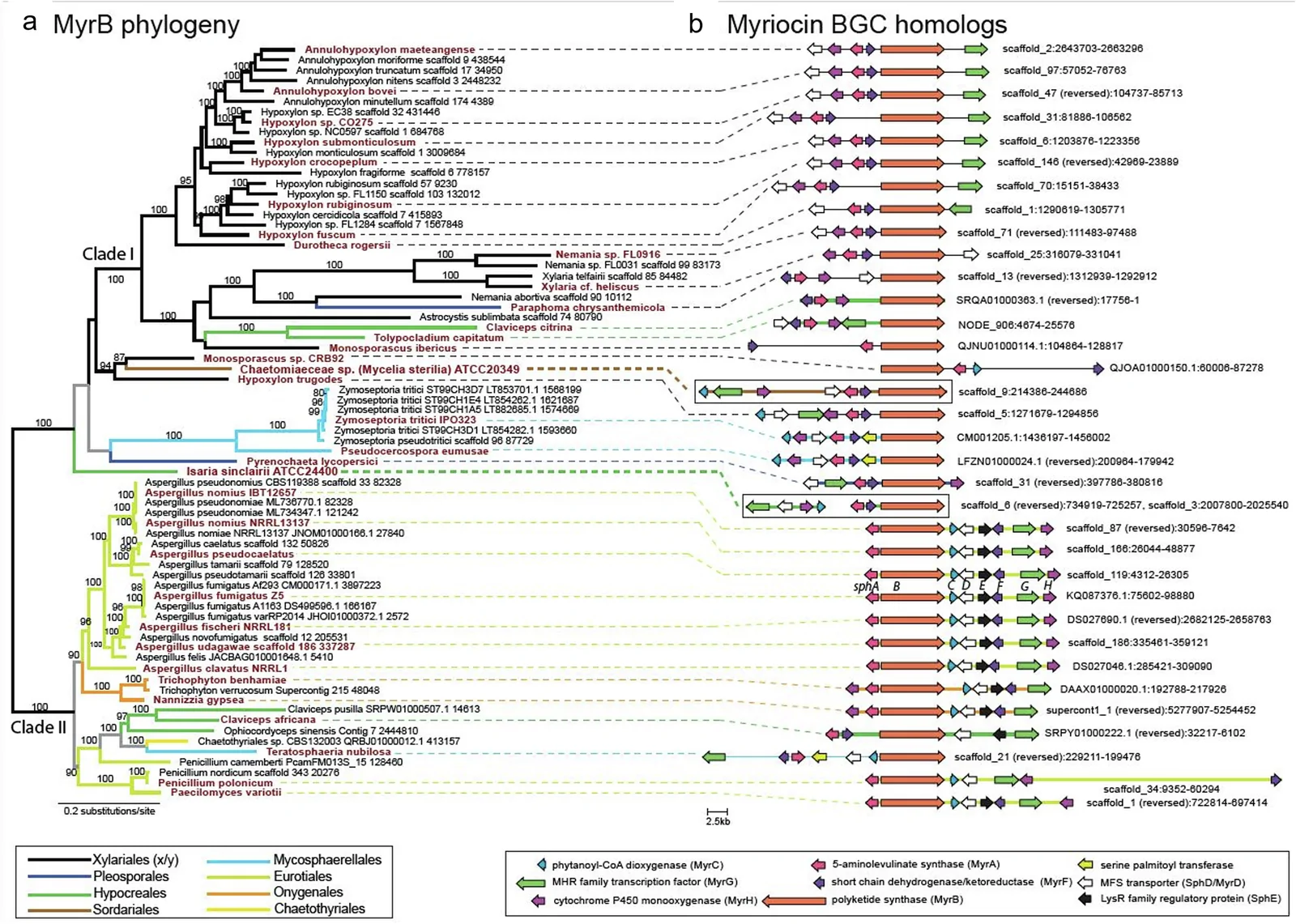
Phylosynteny of the myriocin biosynthetic gene cluster. **a** Maximum likelihood phylogeny of putatively functional MyrB sequences in Pezizomycotina. Red taxon names indicate MyrB sequences from representative BGCs in (**b**), sampled across the phylogeny. BGC coordinates for the remaining MyrB sequences are given. Support values are the percentage of 1000 ultrafast bootstraps in IQTREE, and only values ≥ 80 are shown. The scale bar and branch lengths signify the number of substitutions per site. b. Representative BGCs are aligned at the MyrB gene to demonstrate shared gene order among BGCs associated with MyrB clades. Gene homologs encoding the sphingofungin pathway (sphA,B,C,D,E,F,G,H) are indicated above the *A. fumigatus* Z5 cluster. Taxonomy at the level of order of genomes containing MyrB is reconstructed on tree branches and shown in connections to BGCs and scaffolds, as shown in the key.

Close homologs of myriocin and sphingofungin BGCs are broadly but sparsely distributed across *Sordariomycetes (Xylariales, Hypocreales*, and *Chaetomiaceae), Dothideomycetes (Pleosporales and Mycosphaerellales)*, and *Eurotiomycetes (Eurotiales, Onygenales*, and *Chaetothyriales)*. A phylosynteny analysis using a database that included 1,112 *Pezizomycotina* genomes demonstrates homology between myriocin BGCs and sphingofungin BGCs (Fig. 7). The 170 members of the BGC family that include the central PKS, *myrB*, were detected through a query of six genes with shared synteny between *I. sinclairii* and *M. sterilia* (*myrA,B,C,F,G,H*) plus sphingofungin BGC genes *sphD* (encoding a Major Facilitator Superfamily transporter) and *sphE* (encoding a ketoreductase, KDRS). These BGCs were inferred to contain between 4 and 8 homologous genes. Similarity in gene order among BGCs largely follows two syntenic patterns that correlate with the two clades in the MyrB gene tree (Fig. 7). Clade I (supported by100 rapid bootstrap replicates [BS]) is composed largely of *Xylariales*. The majority of myriocin BGCs in Clade I contained homologs of 6 genes (*sphD, myrH, myrA, myrF, myrB*, and *myrG*), while *myrC* (a phytanoyl-CoA dioxygenase) was not detected. The bulk of the genes encoding the ‘accessory’ enzymes in Clade I myriocin BGCs (*myr A,F,H, sphD*) lie upstream of the PKS, *myrB*, in the genome. In contrast, Clade II (also supported by 100 BS) is composed largely of *Eurotiomycetes* and mostly contains BGCs with homologs of all 8 query genes. Accessory enzyme genes in Clade II tend to be downstream of *myrB*, except for *myrA*.

Gene order in *M. sterilia* is unique, but similar to two BGCs with which it forms the clade (94% BS) adjacent (without support) to Clade I. The *M. sterilia* BGC contains a large gap between clusters of three (*myrC,G,H*) and four (*sphD, myrAFB*) genes. Interestingly, the BGCs correspond to a similar grouping in the six gene homologs in *I. sinclairii*, which also form two clusters of four (*myrG,sphD, myrH,C*) and three (*myrA,F,B*) genes. However, while in *M. sterilia* the clusters are split by a gap, in *I. sinclairii* the clusters are each on separate scaffolds that correspond to the split in the *M. sterilia* cluster. While one of these scaffolds shares synteny with *M. sterilia* and most of clade I, the other has a unique gene order. The only core enzyme-encoding gene that does not occur in all major clades is *myrC* (phytanoyl-CoA-dioxygenase), which was not found in any clade I BGCs. We also failed to detect

homologs of the LysR family regulatory protein (*sphE*) outside of Clade II. A serine palmitoyl transferase (SPT) was detected only in *Dothideomycetes* BGCs, but in both Clade I and Clade II.

**Fig. 8:**
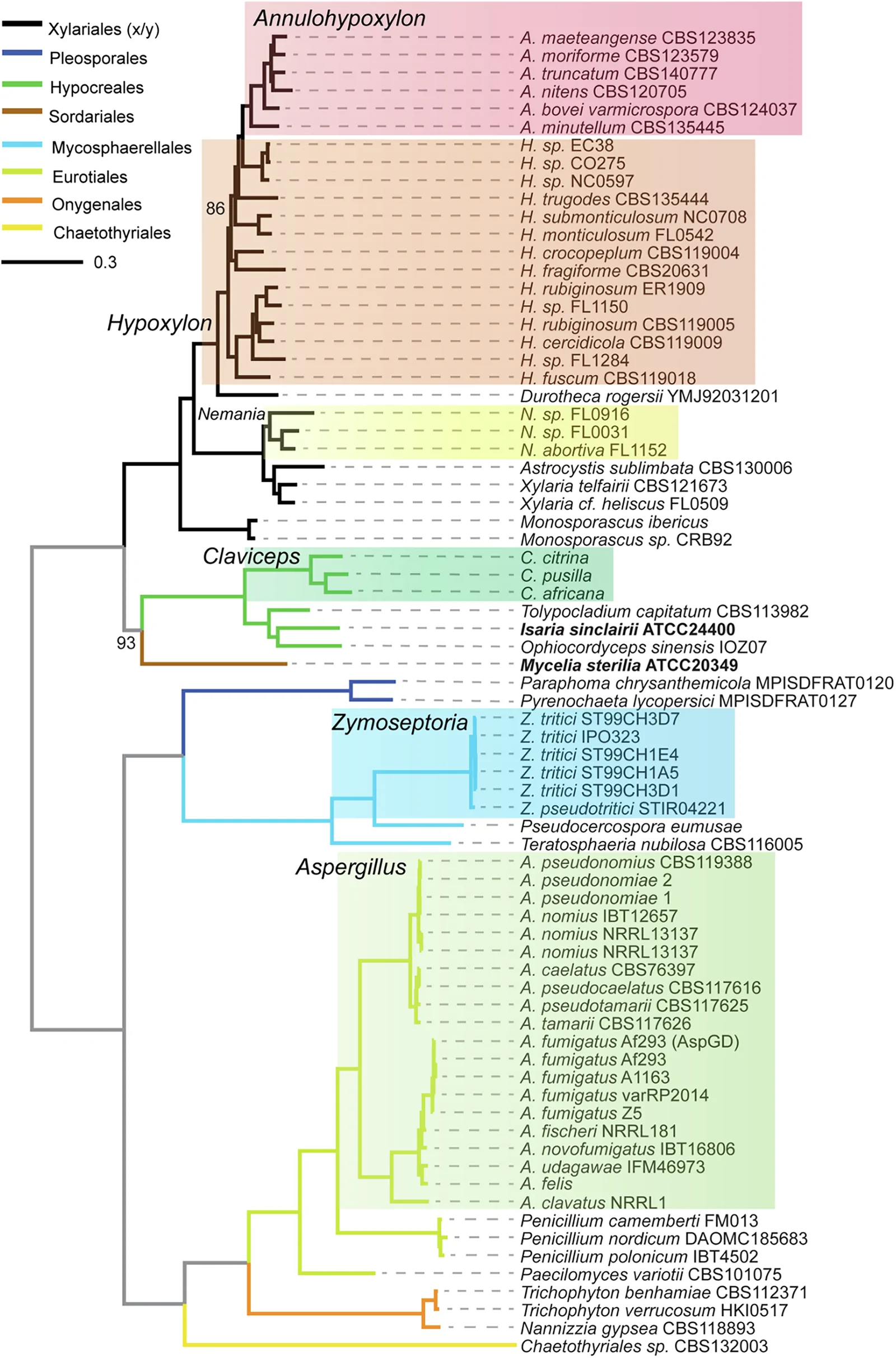
Species phylogeny for all taxa in the MyrB tree. Maximum likelihood tree inferred by IQ-TREE (v.2.2.6 COVID-edition) using 752 single-copy orthologs. All branches were supported by 100% of 1000 ultrafast bootstrap replicates, except for the two nodes indicated by their bootstrap percent values. The scale bar and branch lengths signify the number of substitutions per site.

The dominance of *Xylariales* in *myrB* Clade I and *Eurotiomycetes* in *myrB* Clade II, and general correspondence between the *myrB* tree (Fig. 7) and the species tree within these clades (Fig. 8) (exceptions noted below), suggests myriocin and sphingofungin BGCs have long histories within those two lineages, respectively. We note that a few fungi from other lineages are nested within each clade, suggesting their recent myriocin biosynthesis origins were the result of horizontal gene transfer (HGT). For example, two Hypocreales BGCs, in *Claviceps citrina* and *Tolypocladium capitatum*, are placed with strong support in Clade I, while those of three others (*Claviceps pusila, Claviceps africana*, and *Ophiocordyceps sinensis*) are placed with strong support in Clade II. The first of these putative HGTs is reproduced in the *myrA* and *myrF* gene trees, although those trees are not well supported overall (Supplementary Data Set 2). Similarly, there are two *Pleosporales (*Dothideomycetes) BGCs, one placed deep in Clade I among *Xylariales* species, and the other in an unsupported position sister to an unplaced clade of *Mycosphaerellales* (Dothideomycetes) BGC. The *Teratosphaeria nubilosa* (Mycosphaerellales) BGC is sister to Chaetothyriales sp. CBS132003 (Eurotiomycetes) within Clade I.

The placement of the *M. sterilia* (Sordariales) myriocin BGC in a clade adjacent to Clade I, close to and with a similar gene order to the BGC in *Hypoxylon trugodes* (*Xylariales*), suggests an HGT from *Hypoxylaceae* to *Chaetomiaceae*. By contrast, the fragmented BGC in *I. sinclairii* (*Hypocreales*) is not particularly close to any other taxa in the MyrB phylogeny, leaving its origin cryptic. Overall, the myriocin BGC family appears to have diversified through multiple horizontal transfer events punctuating vertical descent in two main orders (*Eurotiales* and *Xylariales*). It is likely that many BGCs have experienced gene loss and potentially reconfiguration into other gene networks because *myrA* and *myrF* trees contain orthologs of clustered genes from relatives of taxa in the *myrB* tree, and because multiple *myrB* genes were not included due to multiple stop codons in their coding sequences, indicating loss of function.

## Discussion

By sequencing the genomes of two myriocin-producing fungi, *Isaria sinclairii* and *Mycelia sterilia*, we have identified their putative orthologous BGCs involved in myriocin biosynthesis and defined the core genes that encode the enzymes that control the biosynthesis of the major Sphinganine-Like Mycotoxins (SLMs): myriocin, sphingofungin and fumonisin B. This group of myriocin-like secondary metabolites inhibit specific stages of the sphingosine biosynthetic pathway in eukaryotic cells, suggesting that they have co-evolved and been selected during fungal confrontation within the environment. However, each mycotoxin exerts differential effects against other fungi. For example, myriocin is not equally effective against the 5 clades of *Candida auris*, and viridiofungins inhibit serine palmitoyltransferase (SPT) in *C. albicans* but not in *Saccharomyces cerevisiae*^19,45^. Overall, the specificity of each mycotoxin may reflect its required level of efficacy against environmentally relevant adversaries.

Beyond the shared presence of the putative myriocin BGC, there appears to be little similarity in the lifestyles of *M. sterilia* and *I. sinclairii*. *M. sterilia* (ATCC24400) was isolated from a soil sample and is a generic name, as no inducing conditions for spore formation or a morphological transition from the hyphal form have been identified. It can be classed as a thermophile due to its higher growth rate at 45 °C than at 37 °C in permissive media. Genome analysis places *M. sterilia* in the *Chaetomiaceae* family, which includes thermophilic species important for the production of industrially relevant bioactive metabolites^46^. Indeed, the biotechnology industry exploited thermophilic *Chaetomium globosum* and *C. thermophilum* species for myriocin production until the 2010-20 decade, when its production became fully synthetic due to its increased use as a pharmaceutical and the consequent requirement for stricter regulatory control. The ability of *M. sterilia* to produce significantly greater amounts of myriocin by biomass than *I. sinclairii* is therefore consistent with its placement within this industrially relevant *Chaetomiaceae sp. (Sordariomycetes*) family.

While *M. sterilia* is a constitutively filamentous fungus, *I. sinclairii* undergoes a yeast-to-hypha transition between 25 °C and 37 °C. This temperature-directed switch is also seen in *Candida albicans*, which lives as a commensal yeast in humans but undergoes morphogenesis to generate invasive hyphae in its pathogenic form. This switch is the reverse of the major disease-associated pathogens, *Cryptococcus neoformans, Paracoccidioides brasiliensis, Histoplasma capsulatum* and *Coccidioides immitis*, which are filamentous in the environment but grow as yeast at human body temperature. As *I. sinclairii* is an entomopathogen, it is possible that the filamentous form is required for host invasion and the formation of external fruiting bodies for wind-mediated spore dispersal outside the host.

Myriocin and other SLMs are produced by fungi with very differing lifestyles, so it seems unlikely that these sphingolipid inhibitors are targeted directly at a single specific species. For example, the production of sphingofungin by *Aspergillus fumigatus* was not used as a specific defence against predation by amoeba^30^. Instead, the diverse host range of the putative myriocin BGC suggests that it confers a competitive advantage to fungi as a defensive toxin against other fungi within their environment. Both *M. sterilia* and *I. sinclairii* retained and tolerated greater amounts of intracellular myriocin than they secreted, suggesting they have an internal resistance mechanism rather than relying on efflux of the toxin. Indeed, analysis of the MyrD transporter using the SignalP-6.0 server did not identify a localization determinant, so the function of this protein in myriocin metabolism remains to be elucidated.*M. sterilia* may have co-evolved resistance to myriocin as it lies within a related clade of BGC carriers. However, HGT of a BGC that encodes a toxic metabolite is expected to favour recipient hosts with pre-existing tolerance, such as over-expression of, or mutations in, the SPT subunits, or the ability to modify myriocin to reduce binding to SPT^47^. Some BGCs in SLM-producing fungi carry an intrinsic resistance mechanism in the form of bi-functional enzymes that act on both the toxin and native sphingolipid/ceramide synthase pathways. This ensures that self-production of sphingosines is maintained through an increase in production^48,49^, thus avoiding self-poisoning during horizontal gene transfer. However, these BGCs comprise several more genes than the myriocin BGC that we propose here^49,50^. We also note that three putative myriocin BGCs encode an additional SPT, such as those in *Mycosphaerellales* BGCs, which may augment self-production of sphingosines as a resistance mechanism, or may replace the function of impaired native SPTs to enhance the fitness of the pathway during HGT^51^. However, resistance mechanisms can be complex and remain to be elucidated in *I. sinclairii* and *M. sterilia*.

Our finding that aminomalonate is the preferred substrate for MyrA, instead of L-serine, is consistent with recent findings for sphingofungin biosynthesis in *A. fumigatus*^30^, where successful heterologous expression of the sphingofungin BGC in a non-producing fungus, *A. niger*, suggested that aminomalonate may be a readily available precursor in fungi. Further, replacement of the *A. fumigatus* SphA with PvSphA, its ortholog from *Paecilomyces variotii*, produced several sphingofungin variants. Therefore, even though AfSphA could accept only aminomalonate as a substrate, PvSphA was less specific and was able to exploit other aminomalonate derivatives available in *A. fumigatus*. Our work showed that, while aminomalonate is the preferred substrate for *Is*SphA, it is also able to accept L-serine to produce a C_20_-sphinganine product. Therefore, horizontal gene transfer of an SPT-inhibitor cluster might only succeed if a functional self-protection mechanism is in place, for example, contained within the BGC or inherent in the recipient species. The specificity of the candidate AOS and the availability of substrates and any derivatives will determine the number of variants produced and, hence, the effective external range within the environment.

Analyses of the *myrB* distribution and phylogeny combined with comparisons of gene composition and order in homologous BGCs containing *myrB* suggest the proposed myriocin BGC is part of a broadly, but sparsely distributed, BGC family of SLMs that also includes sphingofungin BGCs, as described in *Aspergillus fumigatus* and *Paecilomyces variotii*^30^. The *myrB* phylogeny suggests that two main clades in the SLM BGC family are predominantly found in Xylariales (Clade I) and Eurotiales (Clade II). Within these clades, placement of specific clusters from alternative taxa is consistent with occasional HGT events. Specifically, the myriocin BGC identified in *Chaetomiaceae sp. (Sordariomycetes*) is strongly supported in a clade of *Xylariales* adjacent to Clade I.

The origin of the BGC family remains ambiguous because all genes except the *cis* regulatory gene are distributed throughout most of the tree. Because there is no support for the placement of Clade I, it is unclear as to whether Clade I represents an ancestral state that lacked *myrC*, or the ancestral cluster had all the requisite enzymes, but *myrC* was lost. It is also curious how enzymes are similarly sorted in subclusters in *Chaetomiaceae sp*. and *I. sinclairii* genomes in a way not seen in any others. Given the placement of *I. sinclairii* at the base of the *myrB* tree, it could represent two smaller ancestral BGCs that later merged to become the progenitor of the BGC family. Alternatively, subclusters in these two species might represent branch points in metabolism where alternative genetic networks are driving genomic architecture. Metabolic rewiring could also be suggested by the failure to detect the regulatory protein SphE, but again, it is currently unclear whether such a difference reflects its gain in Clade II vs loss in Clade I.

In summary, this study provides the first genetic and biochemical evidence to support the annotation of the core BGC that enables myriocin production, highlighting the key enzymes responsible for the formation of the fatty acid backbone and the characteristic head group. Moreover, determination of the genome sequences of two myriocin-producing fungi allowed a deep phylogenetic analysis that revealed the widespread presence of this putative BGC across many other fungi. Our assignment of the fungal myriocin BGC is further supported by our recent work in *Pseudomonas fluorescens*, which produces an SLM that protects *C. elegans* from attack by a pathogenic *Bacillus*^52^. In this Gram-negative microbe, an analogous set of core enzymes (PKS, AOS and KDSR) generates the long-chain sphingoid base intermediate that is further tailored to give the mature, bioactive SLM. Our discovery suggests that many other cross-kingdom organisms have the potential to synthesize myriocin and other SLMs, whose structures and functions remain to be elucidated. This study lays the foundation for future work to delete the genes in the putative core myriocin BGC and processing enzymes, and investigate their impact on SLM production. Furthermore, transfer of the identified BGC into a heterologous host accompanied by detection of myriocin production will confirm our annotation and prompt further investigation of the molecular details underpinning the biosynthesis of this important mycotoxin.

## Methods

### Cell culture and imaging

*I. sinclairii* (ATCC24400) and *M. sterilia* (ATCC20349*)* were obtained from the American Type Culture Collection. *I. sinclairii* was stored at −80 °C as 500 µL yeast cells in Tryptic Soy Broth (TSB) (3% (*w*/*v*) tryptic soy (Fisher)) in 500 µL 50% glycerol. *M. sterilia* was stored as agar cubes (excised from a potato dextrose agar plate (PDA)) in 500 µL 50% glycerol. *I. sinclairii* yeast was grown at 25 °C in TSB or on tryptic soy 2% agar (TSA), and hyphae were induced in TSB at 37 °C. Cubes of *M. sterilia* were thawed at room temperature (RT) and placed on PDA or in liquid PD (0.039% (w/v) (Sigma)) at 37 °C with shaking at 200 rpm. *I. sinclairii* nuclei were stained with VECTASHIELD^©^ with DAPI (Vector Laboratories, CA). Fungal cell walls and septa were imaged at 63x magnification on a fluorescence microscope (Zeiss Axiovision), after addition of 10 µg/mL Calcofluour White, centrifugation and washing in ddH_2_O. To measure colony growth, *I. sinclairii* was cultured as yeast, and *M. sterilia* hyphae were picked from a plate and fragmented by vortexing in ddH_2_0. Cells (10 µL) were inoculated onto TSA or PDA plates and incubated at 25, 37 or 45 °C. Colony diameter was measured at 24 d for *I. sinclairii* or 5 d for *M. sterilia*.

### Genome sequencing

Genomic DNA was extracted using a QIAGEN QIAamp kit and sequenced on the Illumina MiSeq (2x300bp) and Oxford NanoPore MinION (R9.4) platforms. gDNA (500 ng) was cleaned using a QIAGEN QIAamp kit. Quantification and quality were checked using a Qubit dsDNA HS Assay on a Qubit Fluorometer 2.0 and Agilent gDNA ScreenTape on an Agilent 2200 TapeStation, respectively. High molecular weight (hmw) gDNA was fragmented to ~ 350 bp using the Diagenode Bioruptor Pico. *M*. *sterilia* lmw gDNA was not fragmented. Fragmentation was confirmed with HSD1000 ScreenTape on the Agilent 2200 TapeStation. Sequencing libraries were prepared using the Illumina TruSeq Nano DNA Library Preparation Kit. The libraries were quantified using the D1000 ScreenTape on the Agilent 2200 TapeStation in conjunction with qPCR and the Kapa Biosciences Complete kit for Illumina library quantification (Universal) on an Illumina Eco qPCR machine. The libraries were diluted and loaded at a ratio of 50% *I. sinclairii* and 50% *M. sterilia* material on the Illumina MiSeq and sequenced with the Illumina MiSeq v3 600 cycle kit. A single Oxford NanoPore MinION library was prepared from fresh independent DNA extracts, using the native barcoding (EXP-NBD103) and ligation kits (SQK-LSK108), and sequenced on a single MinION R9.4 flowcell (FLO-MIN106) for 48 h.

### Genome assembly

Genomes were assembled, scaffolded and polished using a hybrid strategy and assembly completeness was estimated against the OrthoDB v8 fungal reference set of 290 single-copy genes. The raw Illumina data were examined in *fastqc* 0.11.3^53^ and processed with *trim_galore* 0.4.0^54^ to remove Illumina sequence adapters and trim poor-quality bases with a phred score below 30. The trimmed Illumina data were used to estimate genome size, levels of heterozygosity and duplication using the k-mer coverage method. K-mers of 21bp length were counted using *kmc* 3.0.0^55^, and the counts were analysed in *genomescopE*^56^. The raw data were basecalled, demultiplexed and quality-filtered in *albacore* 2.3.1 (Oxford NanoPore Technologies), followed by adapter trimming and another round of demultiplexing in *porechop* 0.2.3 (https://github.com/rrwick/Porechop). These filtering steps retained 1,126,913 reads for *I. sinclairii* (1.106 Gbp) and 2,159,418 reads for *M. sterilia* (2.19 Gbp). A hybrid-assembly strategy was used to combine the benefits of short-read and long-read sequencing technologies. The Illumina data were assembled into short high-quality contigs using the *platanus* 1.2.4 assembler^57^ with an initial k-mer size of 32. Hybrid assemblies were then generated by extending and joining these contigs with the MinION reads using the *dbg2olc* hybrid assembler^58^ followed by consensus generation with two rounds of *racon* 1.3.1^59^ and consensus polishing with five rounds of *pilon* 1.20^60^. In parallel, MinION-only assemblies were generated in *canu* 1.7^61^ and polished with *pilon* for five rounds. MinION-only assemblies were merged with the hybrid assemblies using *quickmerge*^62^, followed by a final round of contig extension and repeat resolution with MinION reads using *finishersc*^63^. The final merged assemblies were then polished a final time with two rounds of *pilon*. Assembly quality statistics were obtained using *quast*^64^, *qualimap* 2.1.1^65^ and *busco*^66^ with the OrthoDB v8 fungal reference set of 290 single-copy genes. Assembly redundancy/duplications with a minimum 90% sequence identity across at least 70% of the contig length were quantified using REDUNDANS 0.14a^67^.

### Identification of the myriocin gene cluster using FungiSMASH

The genomes of *I. sinclarii* and *M. sterilia* were analysed using antiSMASH fungal version 7.1.0; the detection strictness was set to “relaxed”, and the extract features “KnownClusterBlast,” “ClusterBlast,” “SubClusterBlast,” “MIBiG cluster comparison,” “ActiveSiteFinder,” “REFinder,” and “Cluster Pfam analysis” were used^25^. The genes predicted in biosynthetic gene clusters (BGCs) were further searched using NCBI BLAST. The genomic locations were extracted from the contigs using *samtools faidx*^68^. The sequences of the two assemblies were then aligned and inspected in *geneious*. In *I. sinclairii*, the myriocin cluster is designated as Cluster 5 and is located on contig Segkk0, NCBI accession RXPI01000001; in *M. sterilia*, myriocin is Cluster 18 and is located on contig Segkk40, NCBI accession RXPH01000041.

### BGC distribution and diversity

Homologs of the myriocin BGC locus were detected in a database of 2236 fungal genomes (Supplementary Data Set 2) using *CLOCI* v0.1.11 with default parameters as described in Konkel et al. ^69^. Briefly, *CLOCI* circumscribes gene cluster families by identifying homologous loci with unexpectedly shared microsynteny compared to null distributions of randomly sampled loci. Gene cluster family 1920, corresponding to myriocin, was extracted by identifying the similarity network of homologous loci that contain close homologs of MyrB (Supplementary Data Set 2). Best gene models were then determined for each locus using exonerate v.2.2.0 with the protein2genome:bestfit model, minimum identity of 23%, and maximum intron size of 200bp, using manually curated sequences of *myrA, myrB, myrC, myrF, myrG, myrH, spheD*, and *sphE* as queries. All loci were examined for unannotated genes by tblastn of protein models against the genomic scaffold, and gaps in BGCs were examined by blastx against the NCBI nonredundant protein database. Putative pseudogenes were identified by manually checking for at least 2 stop codons in predicted conserved runs of amino acid sequences, and additional sequences with one stop codon were removed after verifying truncation > 30% in preliminary alignments and/or long branches in preliminary phylogenetic analyses. Gene homology and alignment for 34 representative BGCs across the MyrB phylogeny (see below) were determined with Clinker v.0.0.28 using exonerate gene coordinates and a minimum amino acid identity of 22.5%.

### Phylogenetic analyses

Two species trees were generated for this study. To define the taxonomic placement of *Mycelia sterilia*, we inferred a phylogenomic tree of 483 *Sordariomycetes* genomes derived from an alignment of 51 near single-copy genes (Supplementary Data Set 2). *Mycelia sterilia* was provisionally identified as *Chaetomiaceae* sp. based on placement in a clade of *Chaetomiaceae* species with 100% ultrafast bootstrap support. To identify the genes for phylogenomic reconstruction we clustered protein sequences from all proteomes using MMseqs v.14.7e284 implemented in *db2hgs* (Mycotools v.0.30.36) and defined near single-copy genes as those present in *Mycelia sterilia* and missing from at most one genome, with a median copy number per genome of one, and a copy number standard deviation less than 0.5. To identify the best hit protein for these sequences in genomes with multiple copies, we aligned each near single-copy sequence group with MAFFT v.7.487, constructed profile hidden Markov models of alignments using hmmbuild v.3.3.2, and identified the best hit for each model in all genomes the models were present in using hmmsearch implemented in *db2search* (Mycotools). We built the phylogenomic tree by aligning the final sequence set in MAFFT v.7.487, trimming with ClipKIT v.1.3.0, and inferring the multi-gene tree with best evolutionary models applied to each gene partition via IQ-TREE v.2.2.6 COVID-edition.

To compare the MyrB tree to the 74 taxa where it was deemed functional, a phylogenomic tree was generated with these genomes from single-copy orthologs (SCO) determined by OrthoFinder v.2.3.12. Individual amino acid trees were computed for 752 SCO by aligning with MAFFT (v.7.487), trimming via ClipKIT (v.1.3.0), and inferring the maximum likelihood topology in IQ-TREE (v.2.2.6 COVID-edition) with 1000 ultrafast bootstrap replicates. 95 SCO had an average bootstrap support of 90% and were concatenated for phylogenomic analysis (Supplementary Data Set 2). The phylogenomic tree was determined using IQ-TREE with the best evolutionary models for each partition determined by ModelFinder, and support was evaluated by 1000 ultrafast bootstrap replicates. To generate gene trees for MyrA, MyrB, and MyrF, amino acid sequences were aligned with mafft v.7.407 with default parameters, trimmed with trimal v.1.4 using the -automated1 method, and maximum likelihood phylogeny inferred using IQ-TREE v. 1.6.11 with models determined by ModelFinder (MyrA = LG + I + G4, MyrB=JTT + F + R5, MyrF = JTT + I + G4) and branch support evaluated by 1000 ultrafast bootstrap replicates (Supplementary Data Set 2).

### Generation of cluster heatmaps

Pairwise percentage identities for the PKS genes of both fungi were generated using Clustal OMEGA^70^. The PKS open reading frame (ORF) was translated using standard codon usage. Percentage identity for each pairing was used to produce heat maps with 10% bins (0-10%, 11-20%, etc.). Where the sequence of key biosynthetic genes was split across more than one ORF, all ORFs were run, and the ORF producing the highest percentage identities across all its comparisons was used in the heat map. To investigate whether there was extended homology in the putative myriocin clusters, Clustal OMEGA was used to determine percentage identities for each pairwise comparison of the ORFs within the two clusters. BLASTp searches were performed for each of the ORFs annotated as containing “central biosynthetic genes” for each cluster with an assigned type. For clusters where there was more than one central biosynthetic gene ORF, each was run independently. Putative clusters were not examined. The BLASTp search database was restricted to fungi. The top 5 hits returned for each search were examined to determine whether there were any validated proteins. Any proteins denoted as putative or hypothetical were excluded. The top hit, out of hits fitting these criteria, was selected. DNA sequences Segkk0.cluster005 and Segkk40.cluster018 were processed using ExPASy Translate. Open reading frames encoding PKS domains were identified by BLASTp analysis and searched against ClusterCAD^71^ and UniProt to define domain boundaries (with the exception of the inactive SAM-dependent methyltransferase). Nine of the closest myriocin PKS homologues were selected for sequence alignment following identification by BLAST analysis, accounting for both percent identity and query coverage. Sequence alignment, phylogeny and percent identity were performed using Clustal Omega and visualised using ESPript3.0 and Microsoft Excel.

### Sequence analysis

BLASTp was configured to search non-redundant sequences (NRS), UniProt KB/Swiss-Prot or Protein Data Bank (PDB) databases for homologous sequences. MyrB domain boundaries were determined using a combination of ClusterCAD^72^ and BLASTp. Multiple sequence alignments (MSA) and percent identity matrices were computed using Clustal Omega^70^ using default parameters. MSAs were visualised using ESPript 3^73^. Theoretical molecular weights (MWs) and isoelectric points (pI) were computed using ProtScale^74^. *Is*MyrA evolutionary conservation analysis was performed using the ConSurf server configured to build MSAs using MAFFT; 133 homologous sequences with identities ranging from 30–95% were compiled from UNIREF90 using the HMMER search algorithm, and conservation scores were calculated via the Bayesian method and visualised using UCSF ChimeraX (v. 1.6)^75^.

### Structure prediction

All structural predictions were performed using ColabFold^76,77^. In brief, a deep MSA was generated using MMSeqs2 prior to structure prediction using AlphaFold 2 (structural templates were not utilised for prediction). When appropriate, ColabFold was configured to perform multimeric prediction. The output of the AlphaFold 2 structure module was recycled up to 3 times for refinement. For each sequence, a total of 5 models were generated and ranked by Predicted Template Model score (pTM); Predicted Local Distance Difference Test (pLDDT) scores were also computed for each model to evaluate fold-level confidence. The best model was subsequently relaxed to eliminate steric clashes. Visual inspection was performed in UCSF ChimeraX (v1.6) and PyMOL (v2.5.4), and electrostatic potentials were computed using APBS Electrostatics^78^. Topological analysis was performed using the CASTp 3.0 server^79^. For molecular docking studies, both the ligand and receptor were prepared using AutoDockTools, and ligand docking was performed using AutoDock Vina (v1.1.2)^80^. Ligand-receptor hydropathy surfaces were computed in BIOVIA Discovery Studio 2020.

### Myriocin detection by LC-MS

Myriocin biosynthesis was induced by growing *I. sinclairii* or *M. sterilia* in 50 mL fermentation medium (FM) (3% (*w*/*v*) D-glucose (anhydrous), 0.5% (w/v) Bacto peptone (Difco No. 0118-01), 0.3% (*w*/*v*) Yeast extract (Oxoid), 0.03% (*w*/*v*) KH_2_PO_4_ (Monobasic), 0.03% (w/v) K_2_HPO_4_ (dibasic) and 0.03% (w/v) MgSO_4_.7H_2_O. pH was adjusted to 5.5 with HCl prior to autoclaving. for 24d. *I. sinclairii* was incubated with shaking at 25 or 37 °C and *M. sterilia* at 37 or 45 °C^81^. Cells were pelleted at 12,000 rpm and supernatants removed. Cells were desiccated at −105 °C for 2d (Labogene ScanVac CoolSafe) in pre-weighed tubes for determination of dry-weight biomass. Biomass (mg) was determined from desiccated pellets from five independent biological repeats. Supernatants were filtered using a Nalgene Rapid-Flow filter unit (Thermo Scientific) with a 0.2 µm pore size PES membrane and stored at −80 °C. Dried cell pellets (100 mg) were powdered, resuspended in ethyl acetate (5 mL) and sonicated for 10 min. Water (5 mL) was added to wash the ethyl acetate layer, which was then removed, and the aqueous solution was extracted with 5 mL ethyl acetate. Ethyl acetate extracts were combined, dried under reduced pressure and resuspended in 1 mL of acetonitrile/water (90:10), which was further diluted (1:100) for UHPLC-HRMS analysis and myriocin quantification. For culture supernatants, water was added to a total of 40 mL to counter variable evaporation during culture, and 1 mL extracted with ethyl acetate (2 × 5 mL). The combined organic extracts were dried under reduced pressure and resuspended in 1 mL acetonitrile/water (90:10), which was further diluted (1:10) for UHPLC-HRMS analysis and myriocin quantification. Myriocin standard (Sigma, M1177-5MG) was diluted in acetonitrile/water (90:10) to final concentrations of 1, 0.5, 0.25, 0.1, 0.05 and 0.025 µg/mL and analysed by UHPLC-HRMS. Ultra-high-performance liquid chromatography-quadrupole time-of-flight mass spectrometry (UHPLC–QTOFMS) was performed on an Agilent Infinity 1290 UHPLC system (Agilent Technologies, Santa Clara, CA, USA). Separation was achieved on a 2.1 × 50 mm, 2.5 µm, XBridge® BEH C18 column (Waters) fitted with 2.1 × 5 mm, 2.5 µm, XBridge® BEH C18 V-Gd cartridge (Waters) held at 25 °C. The sample (1 µL) was eluted at a flow rate of 0.4 mL/min using a linear gradient from 30% acetonitrile (LC-MS grade) in Milli-Q water buffered with 0.1% formic acid (gradient steps shown in Figure. S12). Mass spectrometry (MS) detection was performed on an Agilent 6545 LC/Q-TOF equipped with an Agilent Dual Jet Stream electrospray ion source (ESI) with a drying gas temperature of 320 °C, a gas flow of 8 L/min, sheath gas temperature of 350 °C and a flow of 11 L/min. Capillary voltage was set to 3500 V and nozzle voltage to 1000 V in positive mode. MS spectra were recorded as centroid data, at an *m/z* of 100–1700, with the scan rate set to 1 spectra/sec. Data were handled using Agilent MassHunter Qualitative Analysis software (Agilent Technologies, Santa Clara, CA). The extracted ion chromatogram (EIC) for mass 402.285 was used to detect myriocin. The area of the peak was integrated and plotted against the known concentration to obtain the calibration curves. The areas of the peak for the EIC (402.258) were used to quantify the amount of myriocin in the extracted samples (Supplementary Data Set 1: https://doi.org/10.6084/m9.figshare.31048009).

### *myrA* and *myrB* RT-PCR

Cells were grown in fermentation medium at 37 °C with shaking at 200 rpm and harvested on Day 10 and Day 20 by centrifugation at 12,100 rpm for 10 min. The cell pellet was resuspended in 3 mL RNALater (Invitrogen, AM7024), snap frozen in liquid N and stored at −80 °C. RNA was extracted from 0.5 mL aliquots of cell suspensions by Monarch Total RNA mini kit (New England Biolabs, T2010), as per the manufacturer’s instructions. Residual DNA was removed with the Turbo DNA-free kit (Invitrogen, AM1907), and RNA was quantified by a Qubit fluorometer that uses fluorescent dye binding specificity to differentiate between DNA and RNA. For cDNA synthesis, 286 ng RNA/sample was diluted in 10 µL H_2_O and 1 µL 100 µg/mL oligo(dT)15 primer (Promega, C1101) was added. Samples were incubated in a thermocycler at 70 °C for 10 min and cooled to 4 °C. Reverse transcription was undertaken using a Moloney murine leukemia virus (MMLV) reverse transcriptase kit (Promega, M1701) with incubation at 37 °C for 60 min and 95 °C for 2 min. Each PCR reaction used Phusion High-Fidelity DNA Polymerase with 2 µL cDNA (New England Biolabs, M0530L) to amplify ~200 bp from actin, *myrA* and *myrB* coding sequences using primers listed in Figure S4b.

### Functional analysis of *Is*MyrA

The 480 aa sequence for *Is*MyrA (Figure S11a) was synthesised by GeneArt and inserted into a pET18 plasmid. Primers *IsmyrA*-F (5’-ctttaagaaggagatataccatggccgctgcatcctttc–3’) and *IsmyrA*-R (5’-ccctgaaaataaagattctctgcgttggcggccatcag–3’) were used to amplify *IsmyrA* and 21 nt overlaps with a linearized pET28 plasmid to generate the pET28IsAOSV3 vector using Gibson assembly: 1 μL plasmid (35 ng), 1 μL gene insert (70 ng) (1–2 ratio), 5 μL HiFi DNA Assembly Master Mix (New England Biolabs), 2.5 μL ddH_2_O, incubated for 45 min at 50 °C. The assembly reaction (2 μL) was added to NEB *E. coli* 5-alpha cells, incubated according to general protocols and plated on LB agar plates (30 μg/mL^−1^ Kanamycin). Positive colonies were confirmed by restriction digest using 300 ng DNA, 2.5 µL (1X) CutSmart buffer 10X (NEB), 0.5 µL *Xba*I (NEB), and/or 0.5 µL *Pst*I enzyme (NEB), ddH_2_O up to 25 µL incubated for 1 h at 37 °C, and analysed by agarose gel. Positive plasmids were sequenced (Eurofins). The resulting IsMyrA product of 52.6 kDa contained a C-terminal TEV-His6 tag and was used for both interaction and modelling studies (Figure S11b and Figure S14).

### Large-scale overexpression of *Is*MyrA

A single *E. coli* BL21(DE3) colony harboring pET28IsAOSv3 (kanamycin 30 µg/mL) was grown in 1 L LB at 37 °C to OD₆₀₀ = 0.6, then cooled on ice. Protein expression was induced with 1 mM IPTG and incubated overnight at 20 °C with shaking. Cells were pelleted (4000 rpm, 20 min), combined, and re-pelleted (8000 rpm, 20 min), yielding a yellow-green pellet. Cells were resuspended in cold binding buffer (20 mM phosphate pH 7.5, 150 mM NaCl, 10 mM imidazole, 25 µM PLP) and lysed by sonication (30 s pulse, 30 s off, 10 cycles). The lysate was pelleted by centrifugation (Fiberlite F15-8x50 cy rotor, 14,000 rpm, 45 min, 4 °C) and the cell-free extract collected. His-tagged *Is*MyrA was purified using a 5 mL HisTrap FF column, washed with binding buffer and eluted with a gradient of 0 to 100% in 25 mL with elution buffer (20 mM phosphate pH 7.5, 150 mM NaCl, 300 mM imidazole, 25 µM PLP) in 2 mL fractions (Figure S11c). Fractions were diluted in exchange buffer (20 mM phosphate pH 7.5, 150 mM NaCl, 25 µM PLP) and concentrated using a 30K MWCO centrifugal  concentrator to a final volume of 1–2 mL. The enzyme was further purified using a Superdex 200 size-exclusion chromatography (SEC) column pre-equilibrated and with run  with exchange buffer (0.5 mL/min) (Figure S11d). Fractions containing *Is*MyrA were combined and concentrated. Storage buffer (20 mM phosphate (pH 7.5), 150 mM NaCl, 25 µM PLP, 50% v/v glycerol) was added to purified *Is*MyrA to a final glycerol concentration of 25% v/v. *Is*MyrA was flash frozen and stored at −80 °C. The yield of purified enzyme was estimated by Bradford assay (~11 mg/L bacterial culture).

### UV–Vis spectrophotometry

*Is*MyrA was converted to the holo-form by 1 h dialysis at 4 °C in 20 mM potassium phosphate (pH 7.5) with 150 mM NaCl and 25 µM PLP. Excess PLP was removed, and protein was concentrated to 10–20 mg/ mL using a 30 kDa VivaSpin filter. UV–Vis spectra (200–800 nm) of 150 μL samples (20 mM phosphate buffer, pH 7.5; 40 μM *Is*MyrA-TEv-His₆ were recorded in quartz cuvettes (Varian Cary 50). To monitor external aldimine formation, L-serine or aminomalonate (0–80 mM final concentration) was added to *Is*MyrA and mixed by pipette. Solutions were incubated for 10 min at RT, and the UV–Vis spectrum plotted using Cary WinUV software (Varian) at 25 °C.

### Substrate interactions

Potential substrates, L-serine and aminomalonate, were prepared as stock solutions of 0.1–50 mM. In quartz cuvettes 1 μL of each substrate concentration was added to 150 μL *Is*MyrA enzyme (10 μM) in 20 mM phosphate buffer (pH 7.5), mixed by pipetting. Solutions were incubated for 10 min at room temperature, and the UV-Vis spectrum was plotted using Cary WinUV software (Varian) (Fig. 6b). The black line in each spectrum is the holo-form of the enzyme (10 mM MyrA, 20 mM potassium phosphate buffer (pH 7.5), 150 mM NaCl, 25 °C). Increasing concentrations of L-serine were added (0, 0.1, 0.5, 1, 2, 5, 10, 20, 40, 60, and 80 mM; colour lines), and the spectrum was recorded after 10 min. For aminomalonate, concentrations were 0, 0.05, 0.1, 0.5, 1, 2, 5, 8, 12, and 16 mM.

### DTNB assay

SPT activity was spectroscopically monitored via the release of CoASH from the reaction of C_16_-CoA/C_18_-CoA with L-serine or aminomalonate (Figure. S11e) (Merch and Santa Cruz Biotechnology) using 5,5’ dithiobis-2-nitrobenzoic acid (DTNB, Ellman’s reagent)^44^. The coloured TNB^2-^ anion was measured at 412 nm (λmax = 412 nm; *ε* = 14,150/M-1cm 1) (Figure S11e). Kinetic assay: in a 96-well plate containing DTNB (0.4 mM), *Is*MyrA enzyme (0.8 mg/mL), L-serine or aminomalonate (5 mM), C_16_-CoA or C_18_-CoA (200 mM), phosphate buffer (20 mM), pH 7.5 was added to a final volume of 100 μL. The reaction mix was preincubated at 30 °C for 30 min before the addition of the enzyme to initiate the reaction. The concentration of C_16_-CoA/C_18_-CoA was maintained at 200 mM. Absorbance was read at 412 nm every 5 s for 30 min in a plate reader (Figure. S11f).

### Activity assay by LCMS

*Is*MyrA-TEv-His₆ (8 mg/mL) was incubated with L-serine or aminomalonate (5 mM) and C_16_-CoA or C_18_-CoA (200 µM) in 20 mM phosphate buffer (pH 7.5, 100 µL total volume). Reactions were run for 180 min at 30 °C with shaking (800 rpm, ThermoMixer C). Heat-inactivated enzyme (15 min, >100 °C) served as a negative control. Reactions were quenched with 100 µL acetonitrile, centrifuged (14,000 rpm, 15 min), and diluted 1:1 with water containing 3% formic acid. Samples were analysed by LC-HRMS (Waters Acquity UPLC Class I Plus with Synapt G2-Si Q-TOF HDMS) using a Premier CSH C18 column (1.7 µm, 2.1 × 100 mm) and VanGuard FIT cartridge. Column temp: 55 °C; flow rate: 0.2 mL/min. Gradient: 95% A:5%B (0–1.5 min), 5% A:95%B (1.5–11 min), 95%A:5%B (11.0–12 min); A: water + 0.1% FA; B: acetonitrile + 0.1% FA.

### Statistics and reproducibility

Statistical analysis was carried out using a 2-tailed Student’s *t*-test in Graphpad Prism 11. Myriocin was extracted from cell pellet biomass (mg) and accompanying supernatant volumes (mL) for both fungi harvested from cultures grown at two temperatures. Myriocin concentrations were measured from five biologically independent samples generated from two independent experiments.

### Reporting summary

Further information on research design is available in the Nature Portfolio Reporting Summary linked to this article.

## Supplementary information

Transparent Peer Review file

Supplementary Information

Description of Additional Supplementary Files

Supplementary Data 1

Supplementary Data 2

Supplementary Data 3A

Supplementary Data 3B

Supplemenary Data 3C

Reporting Summary

## Data Availability

Genome sequences are deposited in NCBI GenBank (Accession numbers: RXPH00000000 (*M. sterilia)* and RXPI00000000 (*I. sinclairii*), BioProject PRJNA509326, and on MycoCosm (Joint Genomes Institute) (*M. sterilia* - https://genome.jgi.doe.gov/Mycester1 and *I. sinclairii* - https://genome.jgi.doe.gov/Isarsinc1). Raw Illumina short-read and Oxford NanoPore MinION long-read genome sequence data are available from NCBI SRA, BioProject PRJNA509326. Numerical source data for Fig. 2a, b and Fig. 3a are labelled as ‘Colony Widths’ and ‘Myriocin Yield’, respectively, and available in Supplementary Data Set 1: Myriocin quantification_Greco: https://doi.org/10.6084/m9.figshare.32325195. Unedited gel images from Fig. 4b, c are shown in Figure. S13. Uncropped gel image from Fig. 6a and Figure. S11b is shown in Figure. S14. Source data for Fig. 6b, d and S11F are available as Rutter et al. Fig. 6b (UV–vis spectrum of IsMyrA), Rutter et al. Fig. 6d (Extracted ion chromatogram) and Figure. S11F (Spectrophotometric detection of the TNB2- anion) in Supplementary Data Set 3_Ortiz at https://doi.org/10.6084/m9.figshare.32652846. Source data for Figs. 7 and 8 are available in Supplementary Data Set 2: Myriocin BCG Phylogeny_Konkel_Slot: https://doi.org/10.6084/m9.figshare.29302175. Plasmid pET28IsAOSV3 is available from Addgene and from the Campopiano lab on request.

## Code availability

The following software, versions and links were used to analyse data in this study: CLOCI v0.1.11 https://github.com/xonq/clocihttps://doi.org/10.1093/nar/gkae625. exonerate v.2.2.0 https://bioweb.pasteur.fr/packages/pack@exonerate@2.2.0 https://doi.org/10.1186/1471-2105-6-31. clinker v.0.0.28 https://github.com/gamcil/clinkerhttps://doi.org/10.1093/bioinformatics/btab007. MMseqs v.14.7e284 https://github.com/soedinglab/MMseqs2https://doi.org/10.1038/nbt.3988. Mycotools v.0.30.36 https://github.com/xonq/mycotoolshttps://doi.org/10.1101/2023.09.08.556886. mafft v.7.487 https://mafft.cbrc.jp/alignment/software/source.htmlhttps://doi.org/10.1093/molbev/mst010. hmmbuild v.3.3.2 http://hmmer.org/download.htmlhttps://doi.org/10.1371/journal.pcbi.1002195. ClipKIT v.1.3.0 https://github.com/JLSteenwyk/ClipKIThttps://doi.org/10.1371/journal.pbio.3001007. IQ-TREE v. 1.6.11 https://github.com/Cibiv/IQ-TREE/https://doi.org/10.1093/molbev/msu300. IQ-TREE v.2.2.6 COVID-edition https://github.com/iqtree/iqtree2/https://doi.org/10.1093/molbev/msaa015. OrthoFinder v.2.3.12 https://github.com/davidemms/OrthoFinder/https://doi.org/10.1186/s13059-019-1832-y. fungiSMASH 7.1.0 https://fungismash.secondarymetabolites.org/#!/start.

## References

[1] Kluepfel, D. et al. Claude Vezina. Myriocin, a new antifungal antibiotic from *Myriococcum albomyces*. *J. Antibiot.* **25**, 109–115 (1972). 10.7164/antibiotics.25.109 5034807

[2] Yoshikawa, M. Asayuki, Yasuhiro Okuno, Y. Y. & Murakami, N. Obutoshi. Total synthesis of a novel immunosuppressant, myriocin (Thermozymocidin, ISP-I), and Z-myriocin. *Chem. Pharm. Bull.* **42**, 994–996 (1994). 10.1248/cpb.42.994 8020136

[3] Šašek, V., Sailer, M., Vokoun, J. & Musílek, V. Production of thermozymocidin (myriocin) by the pyrenomycete *Melanconis flavovirens*. *J. Basic Microbiol.* **29**, 383–390 (1989). 10.1002/jobm.3620290619 2614676

[4] Fujita, T. etsuro et al. Fugal metabolites. Part 11. A potent immunosuppressive activity found in *Isaria sinclairii* metabolite. *J. Antibiot.* **47**, 208–215 (1994). 10.7164/antibiotics.47.208 8150717

[5] Dickson, R. C. & Lester, R. L. Yeast sphingolipids. *Biochim. Biophys. Acta (BBA) - Gen. Subj.* **1426**, 347–357 (1999). 10.1016/s0304-4165(98)00135-4 9878820

[6] Miyake, Y., Kozutsumi, Y., Nakamura, S., Fujita, T. & Kawasaki, T. Serine palmitoyltransferase is the primary target of a sphingosine-like immunosuppressant, ISP-1/myriocin. *Biochem. Biophys. Res. Commun.* **211**, 396–403 (1995). 10.1006/bbrc.1995.1827 7794249

[7] Hannun, Y. & Obeid, L. Ceramide and the eukaryotic stress response. *Biochem. Soc. Trans.* **25**, 1171–1175 (1997). 10.1042/bst0251171 9449970

[8] Im, D.-S. Linking Chinese medicine and G-protein-coupled receptors. *Trends Pharmacol. Sci.* **24**, 2–4 (2003). 10.1016/s0165-6147(02)00012-3 12498721

[9] Van Middlesworth, F. et al. Sphingofungins A, B, C, and D; a new family of antifungal agents i. Fermentation, isolation, and biological activity. *J. Antibiot.* **45**, 861–867 (1992). 10.7164/antibiotics.45.861 1500351

[10] Horn, W. S. et al. Sphingofungins E and F: novel serinepalmitoyl trans-ferase inhibitors from *Paecilomyces variotii*. *J. Antibiot.* **45**, 1692–1696 (1992). 10.7164/antibiotics.45.1692 1474000

[11] Wang, E., Norred, W., Bacon, C., Riley, R. & Merrill, A. H. Jr Inhibition of sphingolipid biosynthesis by fumonisins. Implications for diseases associated with Fusarium moniliforme. *J. Biol. Chem.* **266**, 14486–14490 (1991). 1860857

[12] O’Connor, P. et al. Oral fingolimod (FTY720) in multiple sclerosis: two-year results of a phase II extension study. *Neurology* **72**, 73–79 (2009). 10.1212/01.wnl.0000338569.32367.3d 19122034

[13] Brinkmann, V. et al. Fingolimod (FTY720): discovery and development of an oral drug to treat multiple sclerosis. *Nat. Rev. Drug Discov.* **9**, 883–897 (2010). 10.1038/nrd3248 21031003

[14] Tatematsu, K., Tanaka, Y., Sugiyama, M., Sudoh, M. & Mizokami, M. Host sphingolipid biosynthesis is a promising therapeutic target for the inhibition of hepatitis B virus replication. *J. Med. Virol.* **83**, 587–593 (2011). 10.1002/jmv.21970 21328371

[15] Umehara, T. et al. Serine palmitoyltransferase inhibitor suppresses HCV replication in a mouse model. *Biochem. Biophys. Res. Commun.* **346**, 67–73 (2006). 10.1016/j.bbrc.2006.05.085 16750511

[16] Brakhage, A. A. & Schroeckh, V. Fungal secondary metabolites–strategies to activate silent gene clusters. *Fungal Genet. Biol.* **48**, 15–22 (2011). 10.1016/j.fgb.2010.04.004 20433937

[17] de Melo, N. R. et al. Myriocin significantly increases the mortality of a non-mammalian model host during Candida pathogenesis. *PLoS ONE* **8**, e78905 (2013). 10.1371/journal.pone.0078905 PMC3829820 24260135

[18] Perdoni, F. et al. Antifungal activity of Myriocin on clinically relevant *Aspergillus fumigatus* strains producing biofilm. *BMC Microbiol.* **15**, 1–8 (2015). 10.1186/s12866-015-0588-0 PMC4628231 26519193

[19] Kumar, M. et al. Sphingolipidomics of drug resistant Candida auris clinical isolates reveal distinct sphingolipid species signatures. *Biochim. Biophys. Acta (BBA)-Mol. Cell Biol. Lipids* **1866**, 158815 (2021). 10.1016/j.bbalip.2020.158815 PMC7695621 32942047

[20] Sasaki, S. et al. Fungal metabolites. part 14†. Novel potent immunosuppressants, mycestericins, produced by *Mycelia sterilia*. *J. Antibiot.* **47**, 420–433 (1994). 10.7164/antibiotics.47.420 8195042

[21] Morgenstern, I. et al. A molecular phylogeny of thermophilic fungi. *Fungal Biol.* **116**, 489–502 (2012). 10.1016/j.funbio.2012.01.010 22483047

[22] Hill, R., Leitch, I. J. & Gaya, E. Targeting Ascomycota genomes: what and how big? *Fungal Biol. Rev.* **36**, 52–59 (2021).

[23] Montalbán-López, M. et al. New developments in RiPP discovery, enzymology and engineering. *Nat. Prod. Rep.* **38**, 130–239 (2021). 10.1039/d0np00027b PMC7864896 32935693

[24] Kloosterman, A. M. et al. Expansion of RiPP biosynthetic space through integration of pan-genomics and machine learning uncovers a novel class of lanthipeptides. *PLoS Biol.* **18**, 10.1371/journal.pbio.3001026 (2020). PMC7794033 33351797

[25] Blin, K. et al. antiSMASH 7.0: new and improved predictions for detection, regulation, chemical structures and visualisation. *Nucleic Acids Res.* **51**, W46–W50 (2023). 10.1093/nar/gkad344 PMC10320115 37140036

[26] Chooi, S. C. K.aY. -H. Out for a RiPP: challenges and advances in genome mining of ribosomal peptides from fungi. *Nat. Prod. Rep.* **39**, 222–230 (2021). 10.1039/d1np00048a 34581394

[27] Wang, J., Deng, Z., Liang, J. & Wang, Z. Structural enzymology of iterative type I polyketide synthases: various routes to catalytic programming. *Nat. Prod. Rep.* **40**, 1498–1520 (2023). 10.1039/d3np00015j 37581222

[28] Wang, J. et al. Structural basis for the biosynthesis of lovastatin. *Nat. Commun.* **12**, 867 (2021). 10.1038/s41467-021-21174-8 PMC7870829 33558520

[29] Xie, X., Meehan, M. J., Xu, W., Dorrestein, P. C. & Tang, Y. Acyltransferase mediated polyketide release from a fungal megasynthase. *J. Am. Chem. Soc.* **131**, 8388–8389 (2009). 10.1021/ja903203g PMC2878198 19530726

[30] Bissell, A. U. et al. Biosynthesis of the sphingolipid inhibitors sphingofungins in filamentous fungi requires aminomalonate as a metabolic precursor. *ACS Chem. Biol.* **17**, 386–394 (2022). 10.1021/acschembio.1c00839 35023724

[31] Herbst, D. A., Jakob, R. P., Zähringer, F. & Maier, T. Mycocerosic acid synthase exemplifies the architecture of reducing polyketide synthases. *Nature* **531**, 533–537 (2016). 10.1038/nature16993 26976449

[32] Maier, T., Leibundgut, M. & Ban, N. The crystal structure of a mammalian fatty acid synthase. *Science* **321**, 1315–1322 (2008). 10.1126/science.1161269 18772430

[33] Meinke, J. L. et al. Structural and functional studies of a gem-dimethylating methyltransferase from a trans-acyltransferase assembly line. *ACS Chem. Biol.* **13**, 3306–3314 (2018). 10.1021/acschembio.8b00733 PMC6470028 30371052

[34] Skiba, M. A. et al. Domain organization and active site architecture of a polyketide synthase C-methyltransferase. *ACS Chem. Biol.* **11**, 3319–3327 (2016). 10.1021/acschembio.6b00759 PMC5224524 27723289

[35] Skiba, M. A. et al. Biosynthesis of t-butyl in apratoxin A: functional analysis and architecture of a PKS loading module. *ACS Chem. Biol.* **13**, 1640–1650 (2018). 10.1021/acschembio.8b00252 PMC6003868 29701944

[36] Storm, P. A., Herbst, D. A., Maier, T. & Townsend, C. A. Functional and structural analysis of programmed C-methylation in the biosynthesis of the fungal polyketide citrinin. *Cell Chem. Biol.* **24**, 316–325 (2017). 10.1016/j.chembiol.2017.01.008 PMC5419425 28238725

[37] Kerbarh, O., Campopiano, D. J. & Baxter, R. L. Mechanism of α-oxoamine synthases: identification of the intermediate Claisen product in the 8-amino-7-oxononanoate synthase reaction. *Chem. Commun*. **1**, 60–62 (2006). 10.1039/b511837a 16353092

[38] Shimizu, K. et al. The α-oxoamine synthase gene fum8 is involved in fumonisin B2 biosynthesis in *Aspergillus niger*. *Mycoscience* **56**, 301–308 (2014).

[39] Chen, A. et al. Mechanism-based cross-linking probes capture the *Escherichia coli* ketosynthase FabB in conformationally distinct catalytic states. *Biol. Crystallogr.* **78**, 1171–1179 (2022). 10.1107/S2059798322007434 PMC9435599 36048156

[40] Mindrebo, J. T. et al. Structural basis of acyl-carrier protein interactions in fatty acid and polyketide biosynthesis. in *Comprehensive Natural Products III* (Elsevier, 2020).

[41] Richardson, S. M., Marchetti, P. M., Herrera, M. A. & Campopiano, D. J. Coupled natural fusion enzymes in a novel biocatalytic cascade convert fatty acids to amines. *ACS Catal.* **12**, 12701–12710 (2022). 10.1021/acscatal.2c02954 PMC9594044 36313522

[42] Kao, T.-H., Chen, Y., Pai, C.-H., Chang, M.-C. & Wang, A. H.-J. Structure of a NADPH-dependent blue fluorescent protein revealed the unique role of Gly176 on the fluorescence enhancement. *J. Struct. Biol.* **174**, 485–493 (2011). 10.1016/j.jsb.2011.02.010 21397029

[43] Zhao, P. et al. Crystal structure of the 3-ketodihydrosphingosine reductase TSC10 from Cryptococcus neoformans. *Biochem. Biophys. Res. Commun.* **670**, 73–78 (2023). 10.1016/j.bbrc.2023.05.109 37285720

[44] Raman, M. C. et al. The external aldimine form of serine palmitoyltransferase: structural, kinetic, and spectroscopic analysis of the wild-type enzyme and HSAN1 mutant mimics. *J. Biol. Chem.* **284**, 17328–17339 (2009). 10.1074/jbc.M109.008680 PMC2719368 19376777

[45] Suzanne, M. et al. Viridiofungins, novel inhibitors of sphingolipid synthesis. *J. Antibiot.* **50**, 339–343 (1997). 10.7164/antibiotics.50.339 9186561

[46] Wang, X. et al. Taxonomy, phylogeny and identification of Chaetomiaceae with emphasis on thermophilic species. *Stud. Mycol.* **101**, 121 (2022). 10.3114/sim.2022.101.03 PMC9365047 36059895

[47] Momoi, M. et al. SLI1 (YGR212W) is a major gene conferring resistance to the sphingolipid biosynthesis inhibitor ISP-1, and encodes an ISP-1 N-acetyltransferase in yeast. *Biochem. J.* **381**, 321–328 (2004). 10.1042/BJ20040108 PMC1133791 15025559

[48] Janevska, S. et al. Self-protection against the sphingolipid biosynthesis inhibitor fumonisin B1 is conferred by a FUM cluster-encoded ceramide synthase. *MBio* **11**, 00455–00420 (2020). 10.1128/mBio.00455-20 PMC7298705 32546615

[49] Jojić, K. et al. The spatial organization of sphingofungin biosynthesis in *Aspergillus fumigatus* and its cross-interaction with sphingolipid metabolism. *MBio* **15**, e00195–00124 (2024). 10.1128/mbio.00195-24 PMC10936153 38380921

[50] Gherlone, F. et al. The palmitoyl-CoA ligase Fum16 is part of a *Fusarium verticillioides* fumonisin subcluster involved in self-protection. *mBio* **16**, e02681–02624 (2025). 10.1128/mbio.02681-24 PMC11796371 39704544

[51] Keller, N. P. Translating biosynthetic gene clusters into fungal armor and weaponry. *Nat. Chem. Biol.* **11**, 671–677 (2015). 10.1038/nchembio.1897 PMC4682562 26284674

[52] Peters, L. et al. Polyketide synthase-derived sphingolipids mediate microbiota protection against a bacterial pathogen in *C. elegans*. *Nat. Commun.* **16**, 5151 (2025). 10.1038/s41467-025-60234-1 PMC12134224 40461452

[53] Andrews, S. *FastQC: a Quality Control Tool for High Throughput Sequence Data*, https://github.com/s-andrews/FastQC, Babraham Bioinformatics - FastQC A Quality Control tool for High Throughput Sequence Data (2010).

[54] Krueger, F. *Trim Galore!: a Wrapper Around Cutadapt and FastQC to Consistently Apply Adapter and Quality Trimming to FastQ Files, with Extra Functionality for RRBS Data.* Babraham Institute GitHub - FelixKrueger/TrimGalore: Consistent adapter and quality trimming for NGS, with extra functionality for RRBS GitHub https://github.com/FelixKrueger/TrimGalore (2015).

[55] Kokot, M., Długosz, M. & Deorowicz, S. KMC 3: counting and manipulating k-mer statistics. *Bioinformatics* **33**, 2759–2761 (2017). 10.1093/bioinformatics/btx304 28472236

[56] Vurture, G. W. et al. GenomeScope: fast reference-free genome profiling from short reads. *Bioinformatics* **33**, 2202–2204 (2017). 10.1093/bioinformatics/btx153 PMC5870704 28369201

[57] Kajitani, R. et al. Efficient de novo assembly of highly heterozygous genomes from whole-genome shotgun short reads. *Genome Res.* **24**, 1384–1395 (2014). 10.1101/gr.170720.113 PMC4120091 24755901

[58] Ye, C., Hill, C., Wu, S., Ruan, J. & Ma, Z. DBG2OLC: efficient assembly of large genomes using long erroneous reads of the third generation sequencing technologies. *Sci. Rep*. **6**, 31900 (2016). 10.1038/srep31900 PMC5004134 27573208

[59] Vaser, R., Sović, I., Nagarajan, N. & Šikić, M. Fast and accurate de novo genome assembly from long uncorrected reads. *Genome Res.* **27**, 737–746 (2017). 10.1101/gr.214270.116 PMC5411768 28100585

[60] Walker, B. J. et al. Pilon: an integrated tool for comprehensive microbial variant detection and genome assembly improvement. *PLoS ONE* **9**, e112963 (2014). 10.1371/journal.pone.0112963 PMC4237348 25409509

[61] Koren, S. et al. Canu: scalable and accurate long-read assembly via adaptive k-mer weighting and repeat separation. *Genome Res.* **27**, 722–736 (2017). 10.1101/gr.215087.116 PMC5411767 28298431

[62] Chakraborty, M., Baldwin-Brown, J. G., Long, A. D. & Emerson, J. Contiguous and accurate de novo assembly of metazoan genomes with modest long read coverage. *Nucleic Acids Res.* **44**, e147–e147 (2016). 10.1093/nar/gkw654 PMC5100563 27458204

[63] Lam, K.-K., LaButti, K., Khalak, A. & Tse, D. FinisherSC: a repeat-aware tool for upgrading de novo assembly using long reads. *Bioinformatics* **31**, 3207–3209 (2015). 10.1093/bioinformatics/btv280 26040454

[64] Gurevich, A., Saveliev, V., Vyahhi, N. & Tesler, G. QUAST: quality assessment tool for genome assemblies. *Bioinformatics* **29**, 1072–1075 (2013). 10.1093/bioinformatics/btt086 PMC3624806 23422339

[65] Okonechnikov, K., Conesa, A. & García-Alcalde, F. Qualimap 2: advanced multi-sample quality control for high-throughput sequencing data. *Bioinformatics* **32**, 292–294 (2016). 10.1093/bioinformatics/btv566 PMC4708105 26428292

[66] Simão, F. A., Waterhouse, R. M., Ioannidis, P., Kriventseva, E. V. & Zdobnov, E. M. BUSCO: assessing genome assembly and annotation completeness with single-copy orthologs. *Bioinformatics* **31**, 3210–3212 (2015). 10.1093/bioinformatics/btv351 26059717

[67] Pryszcz, L. P. & Gabaldón, T. Redundans: an assembly pipeline for highly heterozygous genomes. *Nucleic Acids Res.* **44**, e113–e113 (2016). 10.1093/nar/gkw294 PMC4937319 27131372

[68] Li, H. et al. The sequence alignment/map format and SAMtools. *bioinformatics* **25**, 2078–2079 (2009). 10.1093/bioinformatics/btp352 PMC2723002 19505943

[69] Konkel, Z., Kubatko, L. & Slot, J. C. CLOCI: unveiling cryptic fungal gene clusters with generalized detection. *Nucleic Acids Res.* **52**, e75–e75 (2024). 10.1093/nar/gkae625 PMC11381361 39016185

[70] Sievers, F. et al. Fast, scalable generation of high-quality protein multiple sequence alignments using Clustal Omega. *Mol. Syst. Biol.* **7**, 539 (2011). 10.1038/msb.2011.75 PMC3261699 21988835

[71] Eng, C. H. et al. ClusterCAD: a computational platform for type I modular polyketide synthase design. *Nucleic Acids Res.* **46**, D509–D515 (2018). 10.1093/nar/gkx893 PMC5753242 29040649

[72] Tao, X. B. et al. ClusterCAD 2.0: an updated computational platform for chimeric type I polyketide synthase and nonribosomal peptide synthetase design. *Nucleic Acids Res.* **51**, D532–D538 (2023). 10.1093/nar/gkac1075 PMC9825560 36416273

[73] Robert, X. & Gouet, P. Deciphering key features in protein structures with the new ENDscript server. *Nucleic Acids Res.* **42**, W320–W324 (2014). 10.1093/nar/gku316 PMC4086106 24753421

[74] Gasteiger, E. et al. The proteomics server for in-depth protein knowledge and analysis. *Nucleic Acids Res.* **31**, 3784–3788 (2023). 10.1093/nar/gkg563 PMC168970 12824418

[75] Pettersen, E. F. et al. UCSF ChimeraX: Structure visualization for researchers, educators, and developers. *Protein Sci.* **30**, 70–82 (2021). 10.1002/pro.3943 PMC7737788 32881101

[76] Jumper, J. et al. Highly accurate protein structure prediction with AlphaFold. *Nature* **596**, 583–589 (2021). 10.1038/s41586-021-03819-2 PMC8371605 34265844

[77] Mirdita, M. et al. ColabFold: making protein folding accessible to all. *Nat. Methods* **19**, 679–682 (2022). 10.1038/s41592-022-01488-1 PMC9184281 35637307

[78] Jurrus, E. et al. Improvements to the APBS biomolecular solvation software suite. *Protein Sci.* **27**, 112–128 (2018). 10.1002/pro.3280 PMC5734301 28836357

[79] Tian, W., Chen, C., Lei, X., Zhao, J. & Liang, J. CASTp 3.0: computed atlas of surface topography of proteins. *Nucleic Acids Res.* **46**, W363–W367 (2018). 10.1093/nar/gky473 PMC6031066 29860391

[80] Eberhardt, J., Santos-Martins, D., Tillack, A. F. & Forli, S. AutoDock Vina 1.2. 0: New docking methods, expanded force field, and Python bindings. *J. Chem. Inf. Model.* **61**, 3891–3898 (2021). 10.1021/acs.jcim.1c00203 PMC10683950 34278794

[81] Riley, R. T. & Plattner, R. D. in *Methods in Enzymology*, Vol. 311, 348–361 (Elsevier, 2000). 10.1016/s0076-6879(00)11095-x 10563339

