# Supplementary material for: Evolution, structure and function of the putative biosynthetic gene cluster of the fungal secondary metabolite myriocin, a potent inhibitory sphingolipid
Source: Commun Biol. 2026 Jul 17;9:1249. doi: 10.1038/s42003-026-10708-9 (PMC13612406; doi:10.1038/s42003-026-10708-9)
Supplement: Supplementary file 1 [file 42003_2026_10708_MOESM1_ESM.pdf]

nature portfolio

Peer Review File

Evolution, structure and function of the putative biosynthetic gene cluster of the fungal secondary metabolite myriocin, a potent inhibitory sphingolipid

Corresponding Author: Dr Alexandra Brand

This file contains all reviewer reports in order by version, followed by all author rebuttals in order by version.

Version 0:

Reviewer comments:

Reviewer #1

(Remarks to the Author)
This manuscript reports the identification and partial characterization of the biosynthetic gene clusters of a fungal natural product myriocin. Given the medicinal importance of myriocin, this manuscript would be of general interest to the biology community and thus suitable for the journal. However, I have several comments for the authors' consideration and corrections.

- Line 171: Based on the difference in the molecular formulas of this derivative and myriocin, it appears that the derivative is a hydrated product of myriocin. Also, one cannot judge what functional group is introduced just based on the molecular formula. If this compound was not compared with a standard, please just mention the molecular formula.
- Regarding the self-resistance mechanism toward myriocin, have the authors examined the sequences of serine palmitoyltransferase(s) encoded by the producing strains?
- Line 216: The authors mention that three RiPP BGCs were found. However, it is well known that antiSMASH (fungiSMASH) analysis often classifies non-RiPP BGCs as RiPP BGCs. Did the authors find a possible precursor peptide within these BGCs? Similarly, classification as "indole" should be given with care, as some of the "indole" BGCs may not produce metabolites with an indole moiety (Fig. S3).
- Line 230: "Highly reducing" is more commonly used than "fully reducing."
- Lines 247-250: Comparison with characterized PKSs would be more informative.
- Fig. 3b: Please also include the genes in the flanking regions, which would help show the boundaries of BGCs. On the right side of the figure, the gene names should be given without capitalizing the first letter, but with italicization. MFS should be replaced with myrD. Also, I feel it is better if the gene labels are given just above each gene.
- Line 270: "ß" should be replaced with "β."
- Line 280-281: This statement is not always true. Nonenzymatic chain release is known in some PKSs lacking a chain-releasing domain.
- Fig. 4: Predicted functions of MyrC and MyrH should be included in the figure.
- Given the experimental results with MyrA and the structure of myriocin, compound 4c should be accepted by the enzyme. Please discuss the possible origin of this compound.
- Fig. 6: I am wondering whether all BGCs in this figure have been manually inspected. Some BGCs have fewer genes than the myr cluster, but is this not because of annotation errors or other problems in gene prediction?
- This manuscript contains a number of unnecessary capitalizations and italicizations, such as "Polyketide Synthases" and "Ascomycetes" in the abstract. Necessary italicizations are missing somewhere else in the manuscript as well (e.g., "m/z" in line 167). Please carefully check the whole manuscript to correct these errors.

Reviewer #2

(Remarks to the Author)
This article reported the identification of putative biosynthetic gene clusters (BGCs) for myriocin, a strong inhibitor of sphingolipid biosynthesis with immunosuppressant and antifungal activities, through genome sequencing of two fungal producers. Comparative genomic analyses between the two strains strongly suggest genomic regions responsible for myriocin biosynthesis in both strains. This is quite important information for further study of this promising bioactive

compound.
However, the standard approach to identify the BGC of a compound is to delete the candidate genes and confirm the loss of compound production in the native strain, or to perform heterologous/in vitro production of the compound from the corresponding genes. Until such experiments are performed, the cluster is putative and cannot yet be described as the biosynthetic cluster. Although the presented data strongly suggest that the identified BGCs are involved in myriocin biosynthesis, as homologs of myrB are known to participate in the biosynthesis of other PKS compounds like lovastatin, there remains a possibility that these clusters encode other PKS products, or that they are inactive. Thus, gene deletion or heterologous/in vitro expression and production experiments are essential to conclusively identify these clusters as those responsible for myriocin biosynthesis. Such confirmation would also simplify the manuscript by reducing reliance on external assumptions.
Besides that, the analytical and experimental procedures are clear, and the experiment of myrA in E. coli, together with its binding & activity assays to fatty acid substrates, are consistent with the assumption that the cluster is indeed the myriocin BGC. Therefore, I recommend this manuscript for publication in Communications Biology after addressing the following major and minor points.

Major point:
Please conduct gene deletion or heterologous/in vitro production experiments to establish that the identified clusters are responsible for myriocin biosynthesis.

Minor comments:
L.156: Revise “a myriocin BGC” to “a putative myriocin BGC”.
L.172 etc: Ensure consistent use of digits in Mw’s.
L.177: 50%; Use a consistent style, e.g., “1.5-fold”.
Fig.2a: Consider moving this figure to the Introduction, since compounds other than myriocin are not discussed in this section.
Fig.2b: Add y-axes with scale bars for each spectrum.
L.212: Use NCBI accession nos. to designate contigs.
L.223-225: Mention the gene names (myrB and myrA) again here for clarity.
L.224-225: Provide justification for assuming this enzyme’s involvement in myriocin biosynthesis. You might briefly mention the explanation in L.282-289 here, or move it to here.
L.235: Clarify whether there are other syntenic pairs of clusters between the two strains, and explain why the pair of Clusters 5/18 was chosen.
Fig.3b: Consider showing all ORFs, not only the seven highlighted ones; Fig.3a may be better placed in SI.
Fig.5e: Replace “for 1” etc. with numbering directly on the structures, shown in the upper left of each panel.

Reviewer #3

(Remarks to the Author)
In this manuscript, Rutter and co-workers describe the identification of a putative biosynthetic gene cluster (BGC) from I. sinclairii and M. sterilia encoding the sphingolipid inhibitor myriocin. This required establishing cultivation conditions, quantifying, sequencing and assembling two genomes, annotating and comparative genomics. In vitro characterization of the alpha oxo-amine synthase (AOS; MyrA) confirms the reaction between amino-malonate and fatty acid surrogates. In depth structural modelling is utilized to infer mechanistic insights into MyrA, MyrB and MryF. Extensive evolutionary analyses identify homologous BGCs across various fungi and enable an understanding of the origins and conservation of the BGC.
Overall, the manuscript is very well written with beautiful figures. The biological significance of myriocin and structurally similar anti-fungals is especially relevant and topical, therefore the identification of homologous BGCs across so many diverse species of fungi is especially important to the anti-fungal community. The experiments conducted are mostly sufficient and the results / conclusions drawn are valid. However, more direct proof e.g. gene knock-out would have been ideal, but recognizing that this type of experiment is notoriously challenging, the characterization of MyrA will have to suffice.
Specific comments / queries are listed below:
1. A persistent theme was lack of understanding of the self-resistance mechanisms towards these compounds by the producing fungi. Within the BGC is a gene encoding a transporter, is there any way to determine / predict whether this is immediately transporting the mature molecule outside of the cell vs. transporting substrates / intermediates into / between sub-cellular compartments?
2. Alternatively, with now having access to homologous BGCs, could mutations within the serine palmitoyltransferase (SPT) target be identified?
3. During the initial characterization of myriocin in this study, a hydroxylated derivative was also mentioned. If you were to perform HRMS/MS you may be able to determine its exact structure via fragmentation analysis (and also unambiguously confirm myriocin production). The MS spectra provided doesn't show much fragmentation and there appear to be subtle differences between samples, although some of the differences could be noise due to the low concentrations used. Knowledge of this structure could be useful to understand its relevance and whether it has enhanced / diminished biological activities compared to myriocin.
4. The proposed biosynthesis is logical based on genes present and similarities to fumonisin structure / biosynthesis. Have the authors considered RT-PCR / differential expression to confirm that all genes from the BGCs are expressed?
5. The legend of Figure 4 refers to binding affinities but none are provided on the image.
In summary, I think this is an important study supported by in-depth comparative genomics, structural modelling, and characterization of MyrA.

Reviewer #4

(Remarks to the Author)
In Figure 4B, the binding affinities ($kcal·mol^{-1}$) are not displayed, as noted in the legend. For improved clarity and interpretability, I recommend labeling the key residues (e.g., K271 [or possibly L271, if this is a typographical error], N63, H152, and others) directly in Figure 4B.

In addition, on line 342 the manuscript refers to Figures 9a and 9b, but these figures are not present in the main text. It appears that the authors may instead be referring to Figures S9a and S9b. This should be clarified and corrected accordingly.

Version 1:

Reviewer comments:

Reviewer #1

(Remarks to the Author)
I have no additional major comments, but regarding my comment #6, I meant that it would be informative to compare the PKSs with characterized enzymes in Figure 4d (formerly Figure 3c), because the functions of the proteins in this table are unclear.

Reviewer #2

(Remarks to the Author)
The manuscript was nicely revised, particularly in the description of how the authors narrowed down the candidate BGCs to a single pair in the two strains. The authors additionally performed gene expression analyses of myrA and myrB at days 10 and 20. However, these experiments do not replace the gene deletion or heterologous expression analyses required to conclusively identify the BGC responsible for myriocin production. Although gene deletion may not be feasible in the parent strains, heterologous expression of the candidate BGC in an alternative fungal host could be attempted to directly confirm its role in myriocin biosynthesis. Therefore, the additional expression analysis does not exclude alternative possibilities and does not allow a definitive conclusion that this BGC produces myriocin.
Moreover, gene expression under production conditions does not necessarily demonstrate that the genes are directly responsible for compound biosynthesis. In addition, myrA and myrB expression was detected at day 20 but not at day 10, which is inconsistent with the result shown in Fig. 3, where myriocin production is observed after 24 h incubation. This apparent discrepancy should be explicitly addressed and discussed.
While the comparative genome analysis and the in vitro activity assay of MyrA strongly support the hypothesis that the BGC is involved in myriocin biosynthesis, the authors should avoid using the terms “identification” or “identify” to describe the BGC assignment based on the current experimental evidence. More cautious wording is strongly recommended throughout the manuscript.

Minor points:
The legend of Fig. 3 does not correspond to the revised figure.

Reviewer #3

(Remarks to the Author)
The authors have carefully and satisfactorily addressed the reviewer's concerns, providing experimental data for expression of MyrA and B (Figure 4b, c) and clarified areas of the text where there were questions. Therefore, I recommend this study for publication.

Version 2:

Reviewer comments:

Reviewer #1

(Remarks to the Author)
I just have one comment for the authors' consideration. Figure 4d remains the same, and it was unclear to me why MyrB needs to be compared with LovB and LovF; the newly added sentences in lines 314-321 are not very relevant and informative.

Reviewer #2

(Remarks to the Author)
Thank you for revising the title and wording. I have no further comments for publication. I apologize for misreading the incubation time span as 24 hours.

Open Access This Peer Review File is licensed under a Creative Commons Attribution 4.0 International License, which permits use, sharing, adaptation, distribution and reproduction in any medium or format, as long as you give appropriate credit to the original author(s) and the source, provide a link to the Creative Commons license, and indicate if changes were made.
In cases where reviewers are anonymous, credit should be given to 'Anonymous Referee' and the source.
The images or other third party material in this Peer Review File are included in the article's Creative Commons license, unless indicated otherwise in a credit line to the material. If material is not included in the article's Creative Commons license and your intended use is not permitted by statutory regulation or exceeds the permitted use, you will need to obtain permission directly from the copyright holder.
To view a copy of this license, visit https://creativecommons.org/licenses/by/4.0/

COMMSBIO-25-6192-T: RESPONSE TO REVIEWERS

Evolution, structure and function of the biosynthetic gene cluster of myriocin, a potent inhibitory sphingolipid

Note that line-numbers refer to the manuscript in Tracked Changes mode. Reviewer comments have been numbered for ease of reference.

Reviewer Comments

Reviewer 1: This manuscript reports the identification and partial characterization of the biosynthetic gene clusters of a fungal natural product myriocin. Given the medicinal importance of myriocin, this manuscript would be of general interest to the biology community and thus suitable for the journal. However, I have several comments for the authors' consideration and corrections.

1. - Line 171: Based on the difference in the molecular formulas of this derivative and myriocin, it appears that the derivative is a hydrated product of myriocin. Also, one cannot judge what functional group is introduced just based on the molecular formula. If this compound was not compared with a standard, please just mention the molecular formula.

We thank the reviewer for this comment. We have revised the text made it clearer that this compound was likely to be a myriocin derivative, based on the predicted molecular formula and is consistent with a previously described myriocin derivative (Lines 179-182). We have removed the term 'hydrated product' from the manuscript.

2. Regarding the self-resistance mechanism toward myriocin, have the authors examined the sequences of serine palmitoyltransferase(s) encoded by the producing strains?

   Please refer to our response to Reviewer 3, Points 1 & 2, below.

3. - Line 216: The authors mention that three RiPP BGCs were found. However, it is well known that antiSMASH (fungiSMASH) analysis often classifies non-RiPP BGCs as RiPP BGCs. Similarly, classification as "indole" should be given with care, as some of the "indole" BGCs may not produce metabolites with an indole moiety (Fig. S3).

The results we report here derive from the most recently-available version of the commonly-used BGC-identifier software, antiSMASH. We report the output findings from this software in Figure S3 and mention them in the manuscript but we do not claim that we have verified the function of these BGCs and this information may be of interest to others in the field. Instead we used the software to identify BGCs that specifically contain the two genes predicted to be required for myriocin biosynthesis. We have inserted a caveat in the text regarding the potential misidentification of BGCs by antiSMASH as a prediction tool (Line 252-256), and also in the legend of Fig. S3 (Lines 1103-1106).

4. Did the authors find a possible precursor peptide within these BGCs?

We agree this would be a follow-up step in verifying any RiPP BGCs reported by antiSMASH. Given the caveats we state above and the fact that these were not targets of our analyses, further investigation of these clusters is beyond the scope of this study.

5. - Line 230: “Highly reducing” is more commonly used than “fully reducing.”

We have revised the text as per the Reviewer’s suggestion (now Line 275)..

6. - Lines 247-250: Comparison with characterized PKSs would be more informative.

The reviewer recognises that careful examination of the structural features of the putative MyrB, in light of what is known about validated PKSs, would provide support for its proposed function. The reviewer may have missed that this is indeed what we have done in the paragraph starting on Line 333, where each of the domains known to be present in published PKS structures is individually identified and compared in the MyrB proteins. We now guide the reader to this analysis by referring to it in Line 266.

7. - Fig. 3b: Please also include the genes in the flanking regions, which would help show the boundaries of BGCs.

We appreciate this Reviewer’s suggestion to highlight the evidence of cluster boundaries. We now show genes in the flanking regions, which lack additional homology between the focal strains (now Fig 4a).

8. -Fig.3b: On the right side of the figure, the gene names should be given without capitalizing the first letter, but with italicization.

The gene names are now in lower case and italicized (now Fig4.a).

9. -Fig. 3b: MFS should be replaced with myrD. Also, I feel it is better if the gene labels are given just above each gene.

We now show the MFS transporter as *myrD* and the labels now appear above each gene. We have also inserted the predicted gene function underneath the *myrA* and *myrB* genes for ease of reference.

10. - Line 270: “ß” should be replaced with “β.”

This has been revised accordingly.

11. - Line 280-281: This statement is not always true. Nonenzymatic chain release is known in some PKSs lacking a chain-releasing domain.

This statement has been revised to express likelihood rather than certainty (Lines 278-284).

12. - Fig. 4: Predicted functions of MyrC and MyrH should be included in the figure.

The predicted functions of MyrC and MyrH have been included in the figure (now Fig. 5).

13. - Given the experimental results with MyrA and the structure of myriocin, compound 4c should be accepted by the enzyme. Please discuss the possible origin of this compound.

New text has been inserted in Lines (380-384), in which we acknowledge a lack of evidence surrounding the bioavailability or biosynthesis of 2-amino-2-(hydoxymethyl)malonate as a potential substrate for MyrA in *I. sinclairii* and *M. sterilia*.

14. - Fig. 6: I am wondering whether all BGCs in this figure have been manually inspected. Some BGCs have fewer genes than the myr cluster, but is this not because of annotation errors or other problems in gene prediction?

The pipeline for the comparison of BGCs entails searches for all homologs at all loci using tblastn. This is the most effective way of identifying genes when annotations vary in their completeness and accuracy. We originally neglected detailing this step in the methods, but have now included a sentence about it (lines 720-722). We used *exonerate* to model the size and location of each gene de novo. As a follow-up, suspicious gaps were interrogated by blastx against the NCBI non-redundant protein database in order to account for all sequence space in each locus. This resulted in the uncovering of an additional homologous gene (serine palmitoyl transferase) that was not part of the original search. Overall, this is a highly thorough examination of the homology among loci.

15. - This manuscript contains a number of unnecessary capitalizations and italicizations, such as “Polyketide Synthases” and “Ascomycetes” in the abstract. Necessary italicizations are missing somewhere else in the manuscript as well (e.g., “m/z” in line 167). Please carefully check the whole manuscript to correct these errors.

Now revised and corrected.

Reviewer 2: This article reported the identification of putative biosynthetic gene clusters (BGCs) for myriocin, a strong inhibitor of sphingolipid biosynthesis with immunosuppressant and antifungal activities, through genome sequencing of two fungal producers. Comparative genomic analyses between the two strains strongly suggest genomic regions responsible for myriocin biosynthesis in both strains. This is quite important information for further study of this promising bioactive compound.
However, the standard approach to identify the BGC of a compound is to delete the candidate genes and confirm the loss of compound production in the native strain, or to perform heterologous/in vitro production of the compound from the corresponding genes. Until such experiments are performed, the cluster is putative and cannot yet be described as the biosynthetic cluster. Although the presented data strongly suggest that the identified BGCs are involved in myriocin biosynthesis, as homologs of myrB are known to participate in the

biosynthesis of other PKS compounds like lovastatin, there remains a possibility that these clusters encode other PKS products, or that they are inactive. Thus, gene deletion or heterologous/in vitro expression and production experiments are essential to conclusively identify these clusters as those responsible for myriocin biosynthesis. Such confirmation would also simplify the manuscript by reducing reliance on external assumptions.
Besides that, the analytical and experimental procedures are clear, and the experiment of myrA in E. coli, together with its binding & activity assays to fatty acid substrates, are consistent with the assumption that the cluster is indeed the myriocin BGC. Therefore, I recommend this manuscript for publication in Communications Biology after addressing the following major and minor points.

Major point:
1. Please conduct gene deletion or heterologous/in vitro production experiments to establish that the identified clusters are responsible for myriocin biosynthesis.

No gene deletion strategies have yet been developed for these two myriocin-producing fungi. However, in accordance with the Reviewer's suggestion to carry out *in vitro* production experiments, we have undertaken new RT-PCR assays to detect gene expression of *myrA* and *myrB* under the conditions in which we showed that myriocin is biosynthesised. We show that both genes are expressed in these conditions (Lines 287-294). Furthermore, using actin as a constitutively-expressed internal control, we show that although actin is expressed at Days 10 and 20, expression of *myrA* and *myrB* is detectable at Day 20, but not at Day 10. We present these findings as a new Figure 4b. Taken together, the new RT-PCR expression data and the demonstrated function of heterologously-expressed MyrA constitute strong evidence that we have correctly identified the myriocin BGC.

Minor comments:
2. L.156: Revise "a myriocin BGC" to "a putative myriocin BGC".

This has been revised accordingly.

3. L.172 etc: Ensure consistent use of digits in Mw's.

The format of digits has been revised to be consistent.

4. L.177: 50%; Use a consistent style, e.g., "1.5-fold".

The text has been revised to use the '1.5-fold' style.

5. Fig.2a: Consider moving this figure to the Introduction, since compounds other than myriocin are not discussed in this section.

We agree with the Reviewer that this figure is useful in the Introduction, where the SLM-like compounds are first mentioned. This figure has therefore been moved to the Introduction as Figure 1.

6. Fig.2b: Add y-axes with scale bars for each spectrum.

This figure has been updated to include the y-axis on all three chromatograms.

7. L.212: Use NCBI accession nos. to designate contigs.

We have now authorised NCBI to publish the genomes and release the Accession numbers. These have been inserted in the manuscript to replace the original contig identifiers we used in our first submission, which were derived from the MycoCosm submissions of the genome assemblies (Lines 245, 246, 709 and 710).

8. L.223-225: Mention the gene names (myrB and myrA) again here for clarity.

The text has been revised accordingly.

9. L.224-225: Provide justification for assuming this enzyme's involvement in myriocin biosynthesis. You might briefly mention the explanation in L.282-289 here, or move it to here.

Insertion of revised text (Line 265-266) refers the reader to the AOS section in L.282-289 below (now Lines 359-367).

10. L.235: Clarify whether there are other syntenic pairs of clusters between the two strains, and explain why the pair of Clusters 5/18 was chosen.

This point has been addressed in the manuscript by clarifying the step-wise process through which the myriocin BGC was identified, where the search focussed first on identifying the predicted PKS-AOS gene pairing, which was found only in one BGC in each fungus. The identification of a further 5 shared genes in that cluster, along with a high level of synteny, supported the view that this cluster was unique and responsible for myriocin biosynthesis. An analysis of possible synteny in the remaining 45 gene clusters identified in these two fungi was beyond the scope of this study.

11. Fig.3b: Consider showing all ORFs, not only the seven highlighted ones;

We now show up to 5 genes in the flanking regions, which lack additional homology (>/=25% amino acid ID) between the focal strains, and are therefore shown in grey.

12. Fig.3a may be better placed in SI.

Figure 3a has been moved to Supplementary Figures and Tables as a new Fig S4a.

13. Fig.5e: Replace "for 1" etc. with numbering directly on the structures, shown in the upper left of each panel.

The numbering has been revised such that the numbers is Fig. 5e now reflect the numbering used in Fig. 5d (now Figure 6d,e).

Reviewer 3: In this manuscript, Rutter and co-workers describe the identification of a putative biosynthetic gene cluster (BGC) from I. sinclairii and M. sterilia encoding the sphingolipid inhibitor myriocin. This required establishing cultivation conditions, quantifying, sequencing and assembling two genomes, annotating and comparative genomics. In vitro characterization of the alpha oxo-amine synthase (AOS; MyrA) confirms the reaction between amino-malonate and fatty acid surrogates. In depth structural modelling is utilized to infer mechanistic insights into MyrA, MyrB and MryF. Extensive evolutionary analyses identify homologous BGCs across various fungi and enable an understanding of the origins and conservation of the BGC. Overall, the manuscript is very well written with beautiful figures. The biological significance of myriocin and structurally similar anti-fungals is especially relevant and topical, therefore the identification of homologous BGCs across so many diverse species of fungi is especially important to the anti-fungal community. The experiments conducted are mostly sufficient and the results / conclusions drawn are valid. However, more direct proof e.g. gene knock-out would have been ideal, but recognizing that this type of experiment is notoriously challenging, the characterization of MyrA will have to suffice. Specific comments / queries are listed below:

1. A persistent theme was lack of understanding of the self-resistance mechanisms towards these compounds by the producing fungi. Within the BGC is a gene encoding a transporter, is there any way to determine / predict whether this is immediately transporting the mature molecule outside of the cell vs. transporting substrates / intermediates into / between sub-cellular compartments?
2. Alternatively, with now having access to homologous BGCs, could mutations within the serine palmitoyltransferase (SPT) target be identified?

Mechanisms that impart self-resistance to myriocin are an interesting and important topic. Our analysis of the phylogeny across the available genomes containing myriocin-like BGCs provides a starting point for such an investigation. However, meaningful characterization of SPT sequence differences or transporter function and localization is exceptionally complex and beyond the scope of this study. Instead, we have focused on the metabolism, expression, and evolution of these newly-identified, myriocin-producing BGCs. However, we can report that we ran the predicted protein sequences of the MFS (*myrD*) genes through the SignalP-6.0 server but found no indications of a localization determinant in the N or C termini of these proteins. We have inserted this additional finding in Lines 614-618.

3. During the initial characterization of myriocin in this study, a hydroxylated derivative was also mentioned. If you were to perform HRMS/MS you may be able to determine its exact structure via fragmentation analysis (and also unambiguously confirm myriocin production). The MS spectra provided doesn't show much fragmentation and there appear to be subtle differences between samples, although some of the differences could be noise due to the low concentrations used. Knowledge of this structure could be useful to understand its relevance and whether it has enhanced / diminished biological activities compared to myriocin.

We have revised this sentence to make it clear that our suggested formula is a prediction and is consistent with previously published work. The subtle differences between samples are, as the reviewer suggests, likely due to noise in the low concentration present in the samples.

4. The proposed biosynthesis is logical based on genes present and similarities to fumonisin structure / biosynthesis. Have the authors considered RT-PCR / differential expression to confirm that all genes from the BGCs are expressed?

RT-PCR analysis of *myrA* and *myrB* gene expression by RT-PCR has been carried out and are shown as new Fig. 4b. Please see Response to Reviewer 2 above.

5. The legend of Figure 4 refers to binding affinities but none are provided on the image.

The binding affinities have now been inserted into this figure (now Figure 5b).

Reviewer 4: No overall comment.

1. In Figure 4B, the binding affinities ($kcal \cdot mol^{-1}$) are not displayed, as noted in the legend.

The binding affinities have now been inserted into this figure (now Fig. 5).

2. For improved clarity and interpretability, I recommend labeling the key residues (e.g., K271 [or possibly L271, if this is a typographical error], N63, H152, and others) directly in Figure 4B.

This residue has now been labelled in Figure 4B (now Fig. 5b) and the typographical error has been corrected from L_{271} to K_{271}.

3. In addition, on line 342 the manuscript refers to Figures 9a and 9b, but these figures are not present in the main text. It appears that the authors may instead be referring to Figures S9a and S9b. This should be clarified and corrected accordingly.

This figure reference has been revised to refer to the figures in the Supplementary Information (now Fig. S10).

MRC Centre for Medical Mycology
Room 319, Geoffrey Pope Building
University of Exeter
Stocker Road
Exeter EX4 4QD

31 March 2026

Re: Resubmission of Article COMMSBIO-25-6192-A

Response to Referees

Evolution, structure and function of the putative biosynthetic gene cluster of myriocin, a potent inhibitory sphingolipid

We thank the referees for their positive reviews of our manuscript. Below, we describe (in blue font) how we have addressed the comments received and I am pleased to resubmit the manuscript for consideration.

Reviewer comments and author responses:

Reviewer #1:
"I have no additional major comments, but regarding my comment #6, I meant that it would be informative to compare the PKSs with characterized enzymes in Figure 4d (formerly Figure 3c), because the functions of the proteins in this table are unclear."

We thank the referee for this comment. Accordingly, we have now expanded our analysis (primarily in lines 309-321 of the manuscript) to further explain and support our annotation of the MyrB as an iPKS. We have chosen an appropriate and related system, fungal lovastatin biosynthesis, that contains two well-characterised PKS systems; LovB and LovF and we cite the corresponding JACS and Nature Comms papers that describe the biochemical analysis and cryo-EM structures of these iPKSs. We also cite a recent Natural Product Report of these interesting PKS enzymes.

Reviewer #2:
Point 1: "The manuscript was nicely revised, particularly in the description of how the authors narrowed down the candidate BGCs to a single pair in the two strains. The authors additionally performed gene expression analyses of myrA and myrB at days 10 and 20. However, these experiments do not replace the gene deletion or heterologous expression analyses required to conclusively identify the BGC responsible for myriocin production. Although gene deletion may not be feasible in the parent strains, heterologous expression of the candidate BGC in an alternative fungal host could be attempted to directly confirm its role in myriocin biosynthesis. Therefore, the additional expression analysis does not exclude

alternative possibilities and does not allow a definitive conclusion that this BGC produces myriocin."
"While the comparative genome analysis and the in vitro activity assay of MyrA strongly support the hypothesis that the BGC is involved in myriocin biosynthesis, the authors should avoid using the terms "identification" or "identify" to describe the BGC assignment based on the current experimental evidence. More cautious wording is strongly recommended throughout the manuscript."

We appreciate this reviewer's comments regarding the deletion of the proposed myriocin BGC or the movement of the BGC to a heterologous host as being the 'definitive conclusion'. In the absence of gene deletion as the defining factor, due to the current lack of genetic tools in these fungi, we have amended our wording by adding "putative" to the title and softening the language throughout the manuscript. We conclude in the manuscript that our combined genetic, biochemical and bioinformatic analyses lays the foundation for future studies of myriocin biosynthesis.

Point 2: "Moreover, gene expression under production conditions does not necessarily demonstrate that the genes are directly responsible for compound biosynthesis. In addition, myrA and myrB expression was detected at day 20 but not at day 10, which is inconsistent with the result shown in Fig. 3, where myriocin production is observed after 24 h incubation. This apparent discrepancy should be explicitly addressed and discussed."

The Reviewer appears to have misread the manuscript. All descriptions of the detection of myriocin are correctly cited as derived from cultures incubated for 24 days and not for 24 hours. Therefore, the time-frame in which we could detect both expression of the gene cluster (by RT-PCR) and the myriocin metabolite (by UHPLC-HRMS) align within the culture period of 20 - 24 days.

Point 3: "The legend of Fig. 3 does not correspond to the revised figure."

We have corrected the legend for Figure 3 by removing the reference to the chemical structures, which were transferred to Figure 1 in our first resubmission.

Reviewer #3
"The authors have carefully and satisfactorily addressed the reviewer's concerns, providing experimental data for expression of MyrA and B (Figure 4b, c) and clarified areas of the text where there were questions. Therefore, I recommend this study for publication."

We thank the Reviewer for this recommendation. No further revisions are required.

Alexandra C Brand, PhD, FRSB
Associate Professor & Wellcome Senior Research Fellow
MRC Centre for Medical Mycology

COMMSBIO-25-6192-B

Evolution, structure and function of the putative biosynthetic gene cluster of the fungal secondary metabolite myriocin, a potent inhibitory sphingolipid

Response to Reviewer #1

Reviewer #1 (Remarks to the Author):

I just have one comment for the authors' consideration. Figure 4d remains the same, and it was unclear to me why MyrB needs to be compared with LovB and LovF; the newly added sentences in lines 314-321 are not very relevant and informative.

We have addressed the query made by Reviewer #1 regarding the extent to which the MyrB homologs presented in Figure 4d were relevant to our description in the text of the iPKS enzymes, LovB and LovF. To do this, we have revised the 4d figure legend (lines 1152-1154) and the text (Lines 239-254) in order to clarify and separate the very different points that each of these makes: Figure 4d indicates the result of a BLASTp search using MyrB in order to ascertain the distribution of its closest homologs across fungal BGCs. The search revealed that multiple close homologs exist with high levels of sequence identity (64.3 – 95.6%) in specific genera of fungi but, in the text, we point out that, although this tells us of their presence, their structure and function are yet to be fully characterised. However, in the text, we then discuss the fully-characterised LovB and LovF iPKSs, which do not feature in Figure 4d due to their lower sequence identity but are cited as examples of iPKSs in which structure and function are known. Their characterisation (and therefore inclusion) is informative because their sharing of domains with MyrB supports our prediction of MyrB structure and function.
