# Supplementary material for: Evolution, structure and function of the putative biosynthetic gene cluster of the fungal secondary metabolite myriocin, a potent inhibitory sphingolipid
Source: Commun Biol. 2026 Jul 17;9:1249. doi: 10.1038/s42003-026-10708-9 (PMC13612406; doi:10.1038/s42003-026-10708-9)
Supplement: Supplementary file 2 [file 42003_2026_10708_MOESM2_ESM.pdf]

SUPPLEMENTARY FIGURES

Evolution, structure and function of the putative biosynthetic gene cluster of the fungal secondary metabolite myriocin, a potent inhibitory sphingolipid

Ben Rutter[1], Michael A Herrera[2], Gustavo Perez Ortiz[2], Claudio Greco[3], Ben Ashley[2], Hai Deng[4], Abigail Hay[1], Cameron Bedford[5], Marius Wenzel[6,7], Stephen Mondo[8], Zachary Konkel[9], Jason C Slot[9], Qinxi Ma[5], Nicolas Helmstetter[5], Rhys A. Farrer[5], Dominic J. Campopiano*[2] & Alexandra C Brand*‡[1,5,10]

[1] School of Medicine, Medical Sciences & Nutrition, University of Aberdeen, Aberdeen, UK

[2] School of Chemistry, University of Edinburgh, Edinburgh, UK

[3] Department of Biosciences, Swansea University, Swansea, UK

[4] Department of Chemistry, School of Natural & Computing Sciences, University of Aberdeen, Aberdeen, UK

[5] Medical Research Council Centre for Medical Mycology at the University of Exeter, Exeter, UK

[6] Centre for Genome-Enabled Biology & Medicine, University of Aberdeen, Aberdeen, UK

[7]School of Biological Sciences, University of Aberdeen, Aberdeen, UK

[8]US Department of Energy Joint Genome Institute, Lawrence Berkeley National Laboratory, Berkeley,, CA 94720, USA.

[9]Department of Plant Pathology, The Ohio State University, Columbus, USA

[10]Living Systems Institute, University of Exeter, Exeter, UK

*Co-senior authors

‡Corresponding author: Alexandra Brand a.brand@exeter.ac.uk

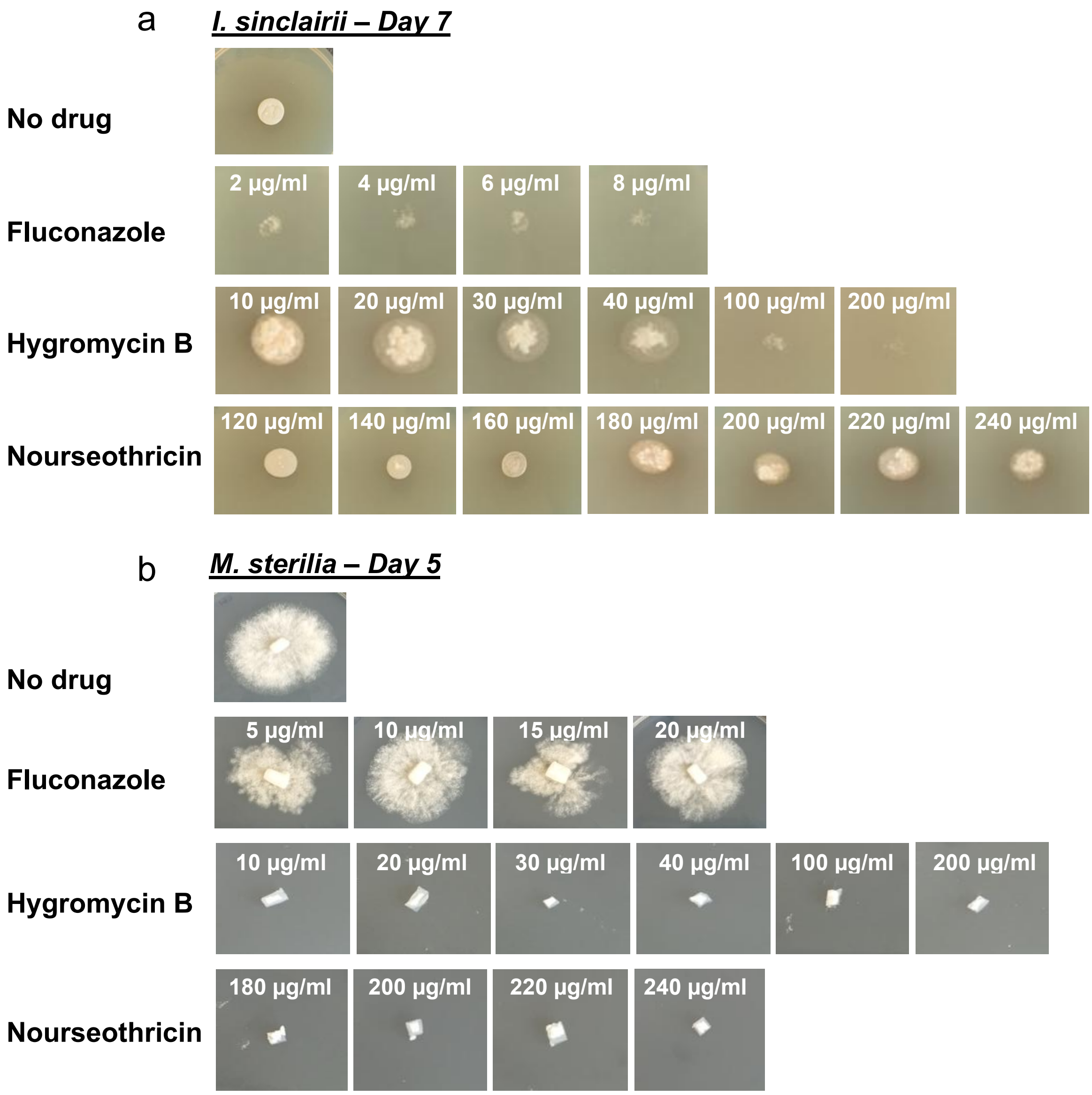

c

Drug	*I. sinclairii* sensitivity (µg/ml)	*M. sterilia* sensitivity (µg/ml)
Fluconazole	< 2	> 20
Hygromycin B	20	< 10
Nourseothricin	240	< 180

Fig. S1: Susceptibility of *I. sinclairii* and *M. sterilia* to fluconazole, hygromycin B and noursethricin. *I. sinclairii* or *M. sterilia* were inoculated onto tryptic soy agar or potato dextrose agar, respectively, containing increasing concentrations of the antifungal agents fluconazole, hygromycin B or nourseothricin. A) *I. sinclairii* plates were incubated at 25 °C for 7 d. B) *M. sterilia* plates were incubated at 37 °C for 5 d. C) Sensitivity was quantified by measuring the ratio of colony diameter compared to the no-drug control.

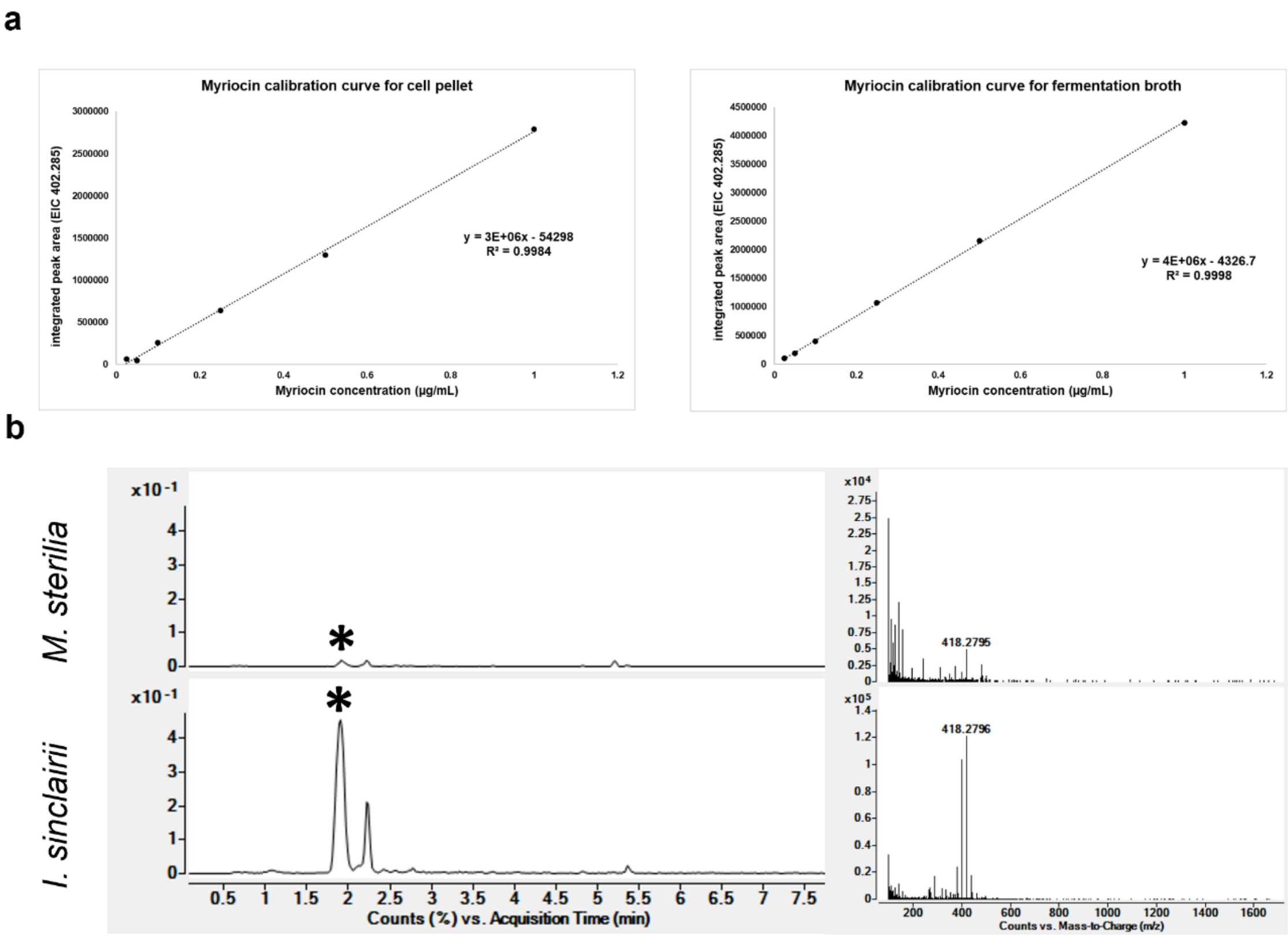

Fig. S2: Myriocin quantification and detection of myriocin derivates. a. Calibration curves for the quantification of myriocin for the cell pellet (left) and for the fermentation broths (right). b. UHPLC-HRMS analysis in positive mode of the organic extracts for the myriocin derivative. EIC for 418.28, noted with an * (left) and mass spectra (right) for *M. sterilia* (top) and *I. sinclairii* (bottom).

a

Assembly parameters	*I. sinclairii*	*M. sterilia*
Total genome size (bp)	25,179,760	34,156,770
Number of contigs	59	141
Maximum contig length (bp)	2,840,681	2,447,423
Mean Illumina coverage	222	139
Mean MinION coverage	42	61
BUSCO completeness (%)	98.6	99.3
N50 value (bp)	1,423,211	817,506
Properly paired (%)	99.63	98.29
GC (%)	57	56
Total number of non-ATGC characters	0	0
Total number of proteins/genes	8167	8706
Secondary metabolite clusters (AntiSMASH)	27	20

b *I. sinclairii*

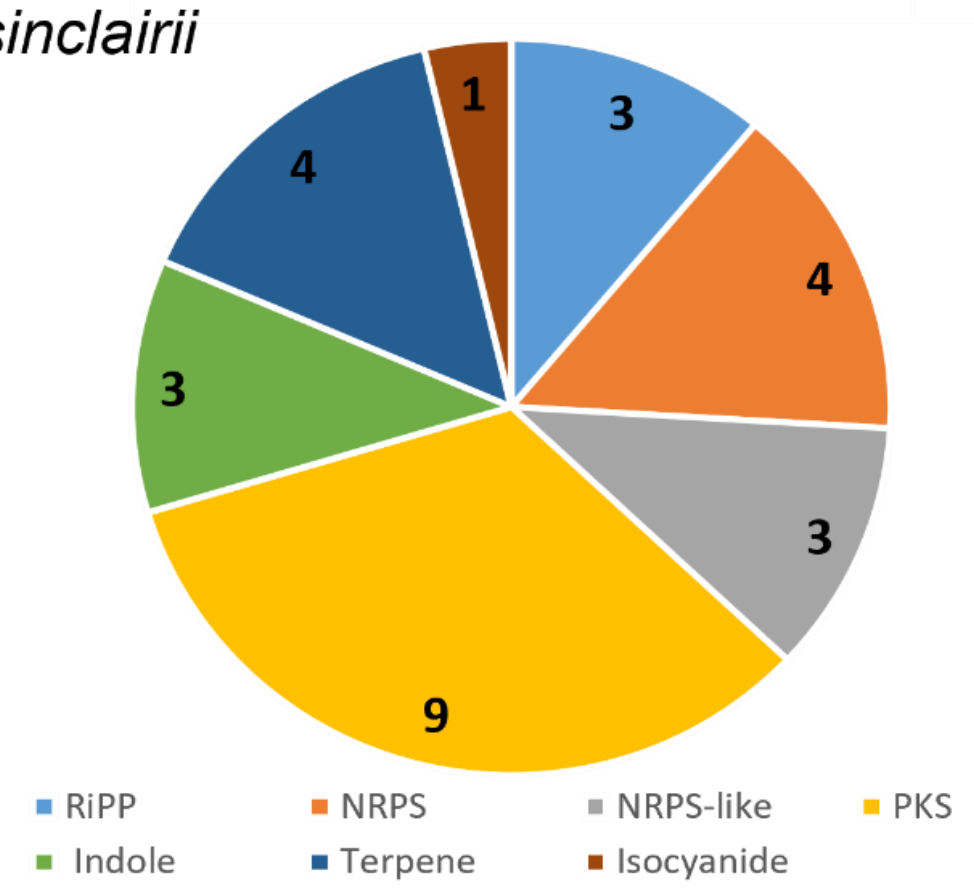

Secondary Metabolite Class	Amount
RiPP	3
NRPS	4
NRPS-like	3
PKS	9
Hybrid PKS - NRPS	0
Indole	3
Terpene	4
Isocyanide	1
**Total**	**27**

c *M. sterilia*

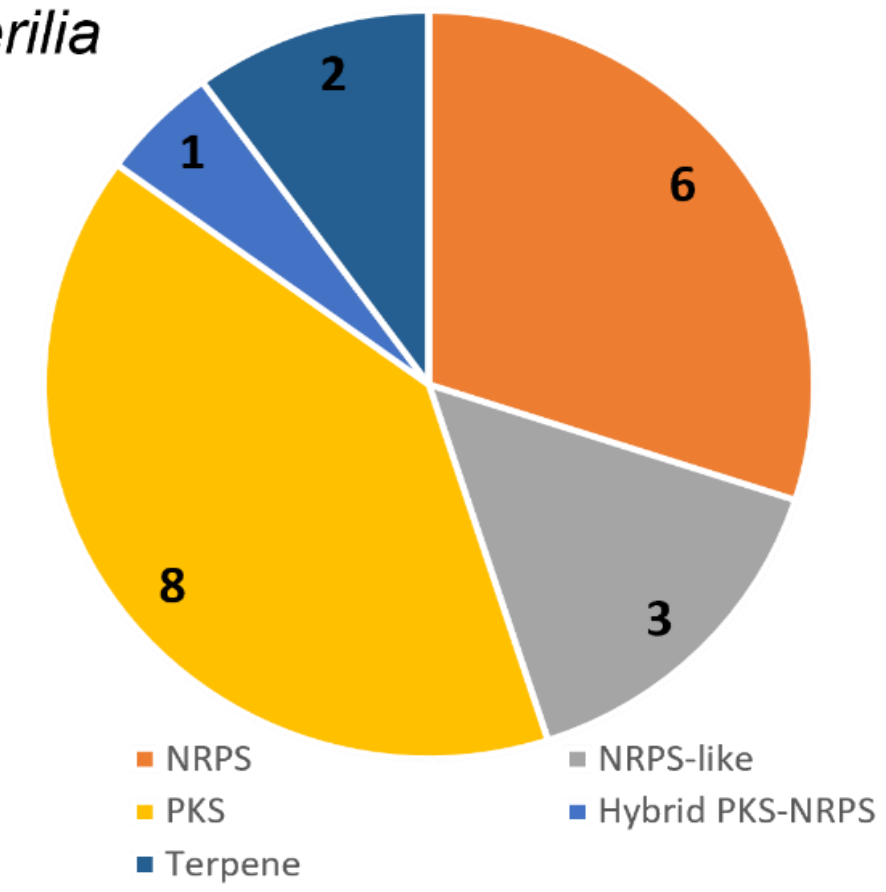

Secondary Metabolite Class	Amount
RiPP	0
NRPS	6
NRPS-like	3
PKS	8
Hybrid PKS-NRPS	1
Indole	0
Terpene	2
Isocyanide	0
**Total**	**20**

Fig. S3: Genome assembly parameters and antiSMASH analysis of secondary metabolite gene clusters by class. a. Comparison of genome assembly parameters for *I. sinclairii* and *M. sterilia*. b,c. Breakdown of secondary metabolite biosynthesis gene clusters by class (AntiSMASH V7.1.0). Putative classifications are based on antiSMASH v7.1.0 fungal mode; RiPP and indole predictions may include false positives and require experimental validation (e.g., via precursor peptide identification or metabolomics). No RiPP BGCs were linked to characterized metabolites in these genomes.

a

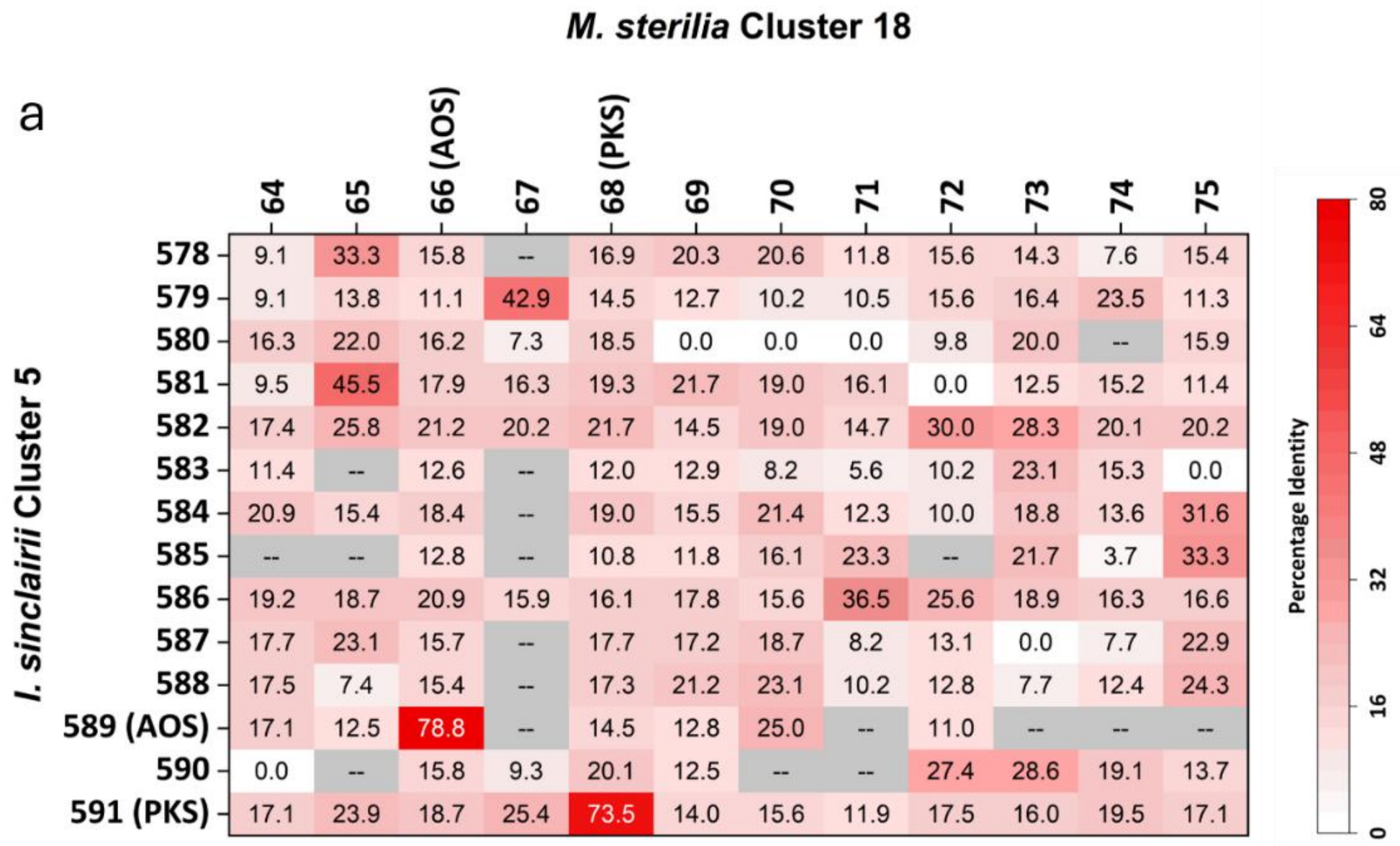

b

Primer name	Primer sequence (5′ – 3′)	Product size (bp)
MsmyrA_1-F	GGCTGGTGAAGCACTTCTT	186
MsmyrA_1-R	CCAGCTGCAGGTGGAAGAC	
MsmyrB_1-F	GCTCGCATGATCGACAACTA	201
MsmyrB_1-R	CGTGAACACCATCACGACGTT	
MsActin-F	CCAACATAGTCCTCTCAGGC	164
MsActin-R	TGAGACCGGCAAGAATACTG	
IsmyrA_1-F	CCATCGACTTCTGCTCCAAC	181
IsmyrA_1-R	CCGTGAAAGTCGGCAATCTC	
IsmyrB_1-F	CGCTCATCAAGATGGTCATG	169
IsmyrB_1-R	CCAAACGAGTTGAGGCTGAT	
IsActin-F	AATCAACCCCAAGTCCAACC	169
IsActin-R	AAATGGGAACAACGTGGGTG	

Fig. S4: a. Identification in *I. sinclairii* and *M. sterilia* genomes of a shared gene cluster revealed the predicted iPKS and AOS genes required for myriocin biosynthesis, which were expressed concomitantly with myriocin biosynthesis. A heatmap indicates the percentage identities for each pair of open reading frames (ORFs) within the two clusters in *M.sterilia* (Cluster 18) and *I. sinclairii* (Cluster 5); b. Primers derived from the above clusters and used for RT-PCR of *myrA*, *myrB* and actin (internal control) mRNA isolated from *I. sinclairii* and *M. sterilia*.

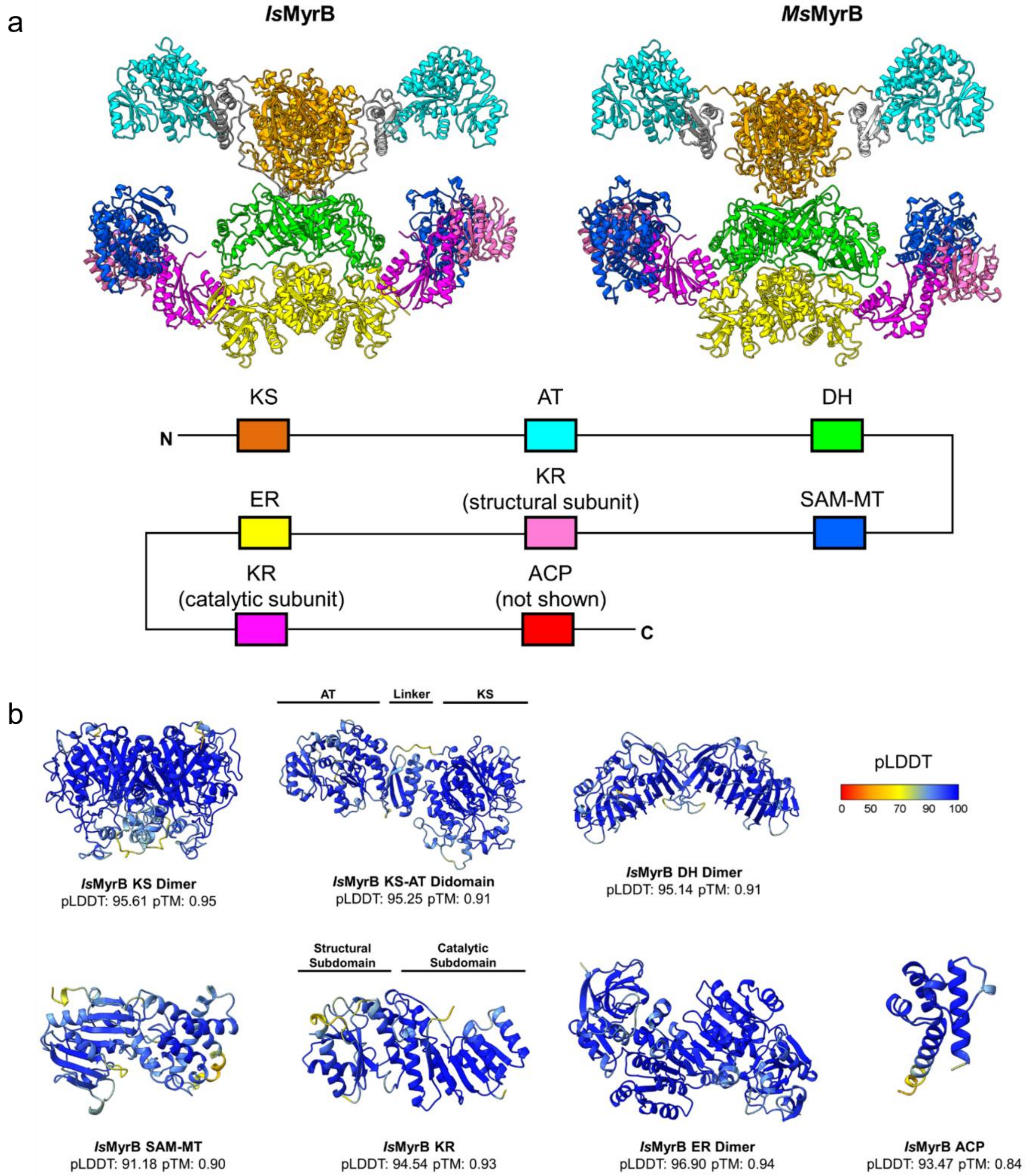

Fig. S5: a. Composite predicted structural models showing the relative domain organisation of the IsMyrB and MsMyrB homodimers. Mammalian fatty acid synthase (PDB: 2VZ8) was used as a template guide, using structural alignment tools in PyMOL. Acyl Carrier Proteins (ACPs) have been omitted. Colours indicate active domains: KS = Ketoacyl Synthase, AT = acytransferase, DH = dehydratase, SAM-MT = *S*-adenosylmethionine-dependent methyltransferase. KR = ketoreductase, ER = enoylreductase. b. The constitutive domains of IsMyrB, coloured by pLDDT confidence score (0-100).

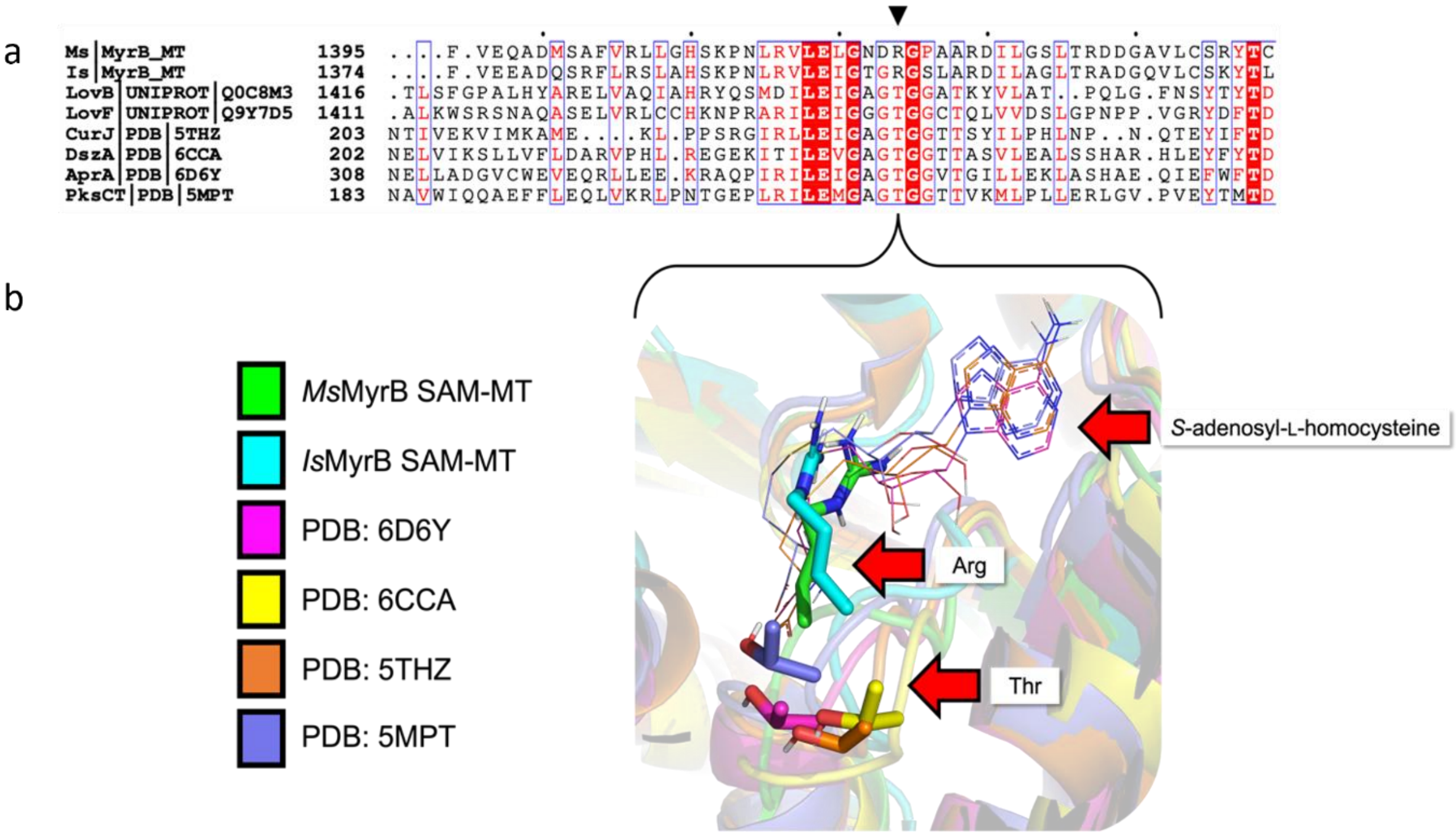

Fig. S6: Occlusion of the *Is*MyrB and *Ms*MyrB *S*-adenosylmethionine binding pocket by a key threonine →arginine mutation. a. Comparison with active, homologous S-adenosylmethionine methyltransferases (SAM-MTs) from the Protein Data Bank revealed a shared mutation in *I. sinclairii* and *M. sterilia* where the 3-carbon threonine is substituted by a 6-carbon arginine (black arrow). b. Modelling suggests that this substitution occludes the S-adenosylmethionine binding pocket, which likely inactivates the domain and correlates with the lack of methyl branching in myriocin. PDB = Protein Data Base.

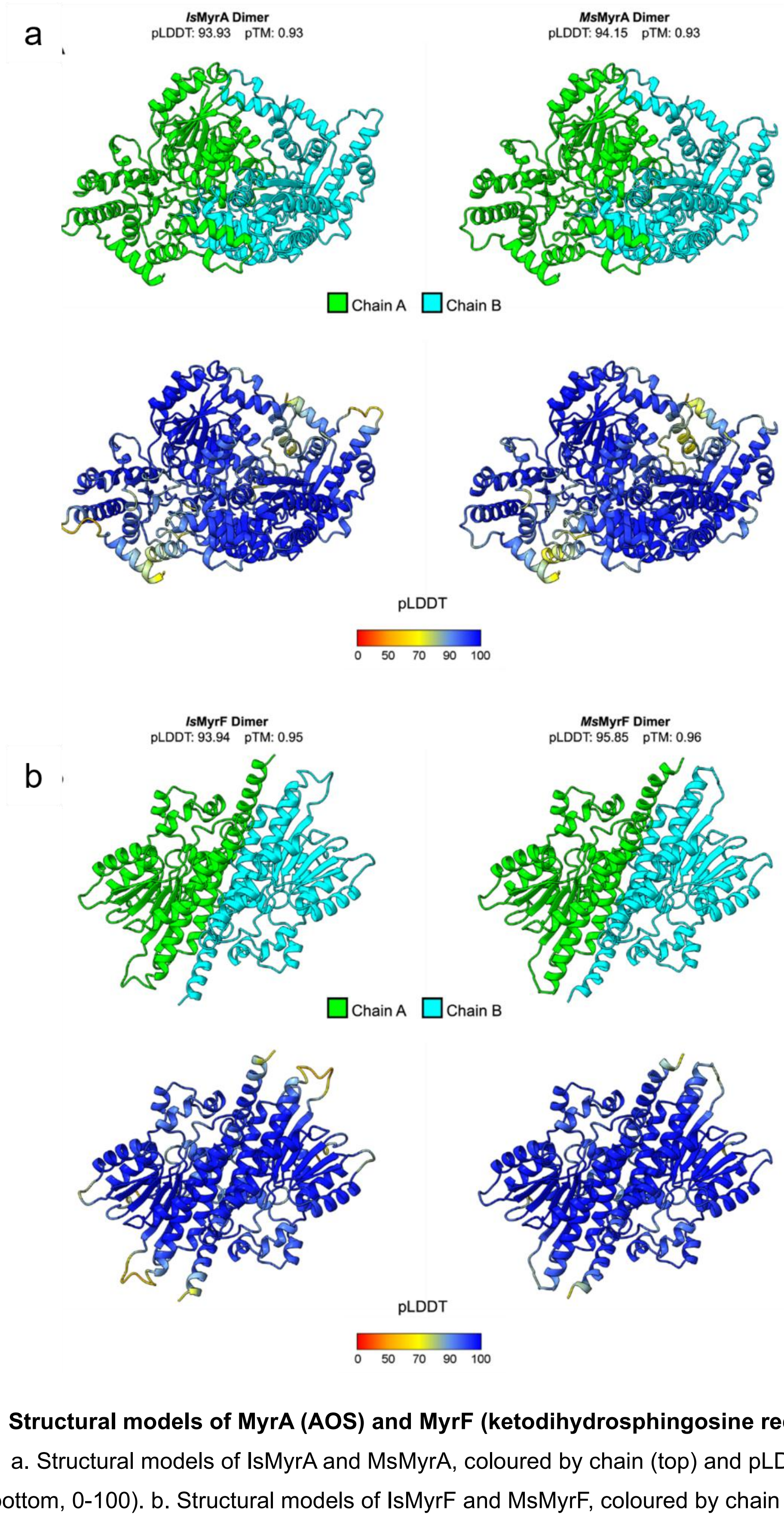

Fig. S7: Structural models of MyrA (AOS) and MyrF (ketodihydrosphingosine reductase – KDRS). a. Structural models of IsMyrA and MsMyrA, coloured by chain (top) and pLDDT confidence score (bottom, 0-100). b. Structural models of IsMyrF and MsMyrF, coloured by chain (top) and pLDDT confidence score (bottom, 0-100).

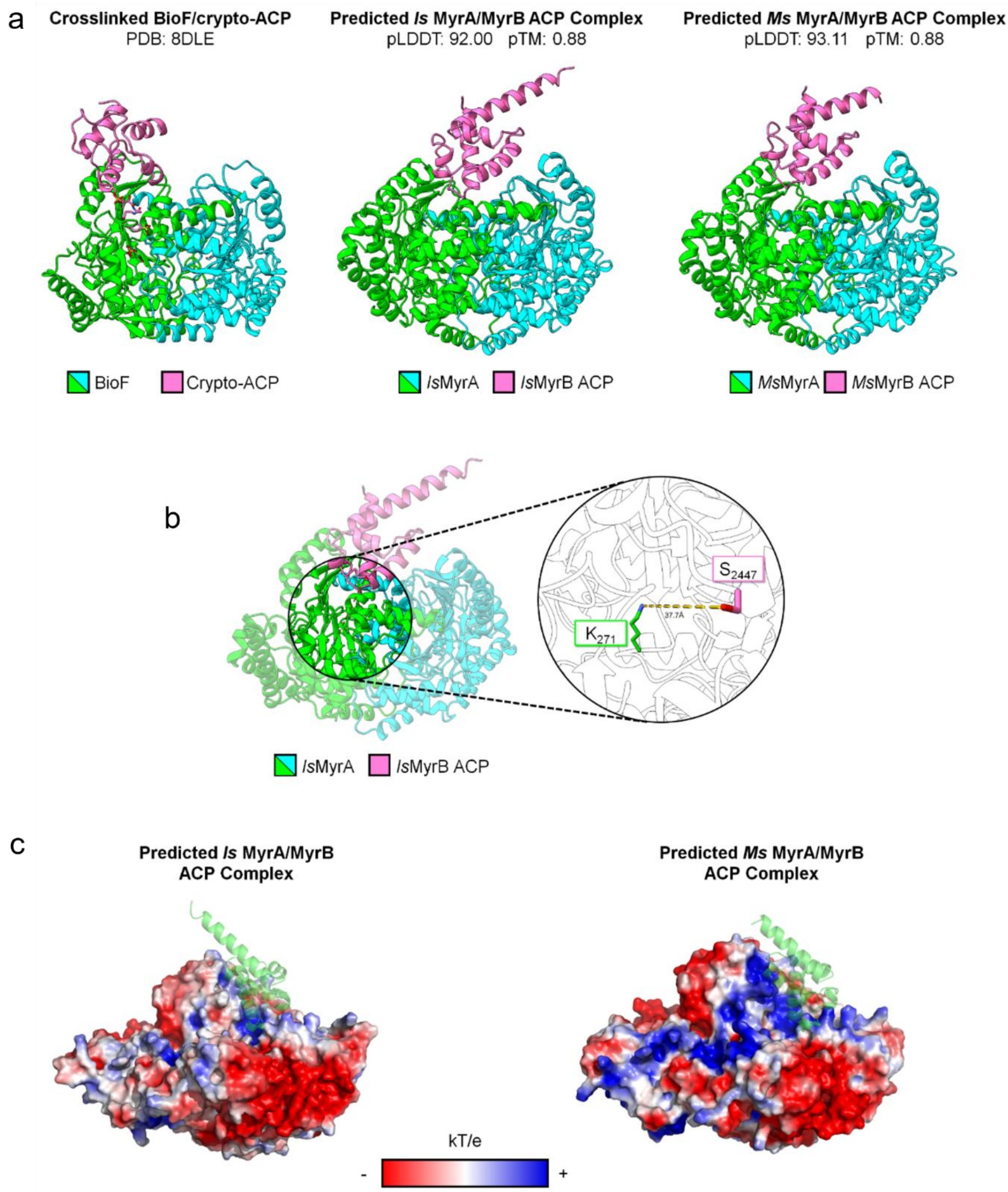

Fig. S8: Interaction between the predicted MyrA (AOS) and MyrB (iPKS) acyl carrier protein complexes. a. Comparison between *E. coli* BioWF/crypto-acyl carrier protein (ACP) experimental structure and the predicted MyrA and *Is*MyrB ACP complexes. b. Predicted docking interface between *Is*MyrA and *Is*MyrB ACP, showing the catalytic *Is*MyrB S_{2447} positioned directly above the substrate binding tunnel of *Is*MyrA. c. Computed MyrA APBS electrostatic surface potentials, identifying patches of electropositivity around the AOS-ACP docking interface. MyrB ACPs are shown in green.

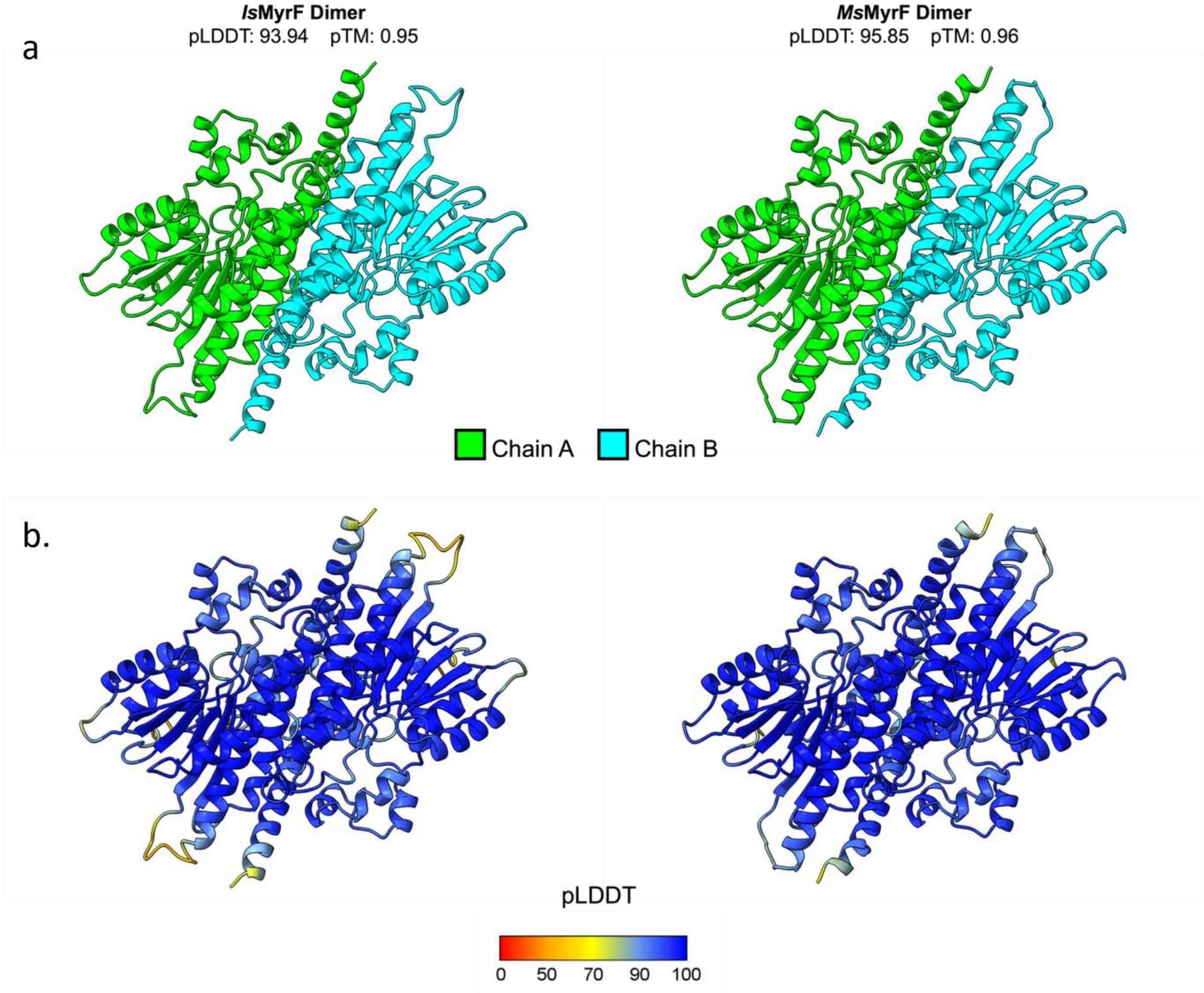

Fig. S9: Structural models of *Is*MyrF and *Ms*MyrF homodimers. a. Models coloured by chain. b. Models colours by pLDDT confidence score, as shown.

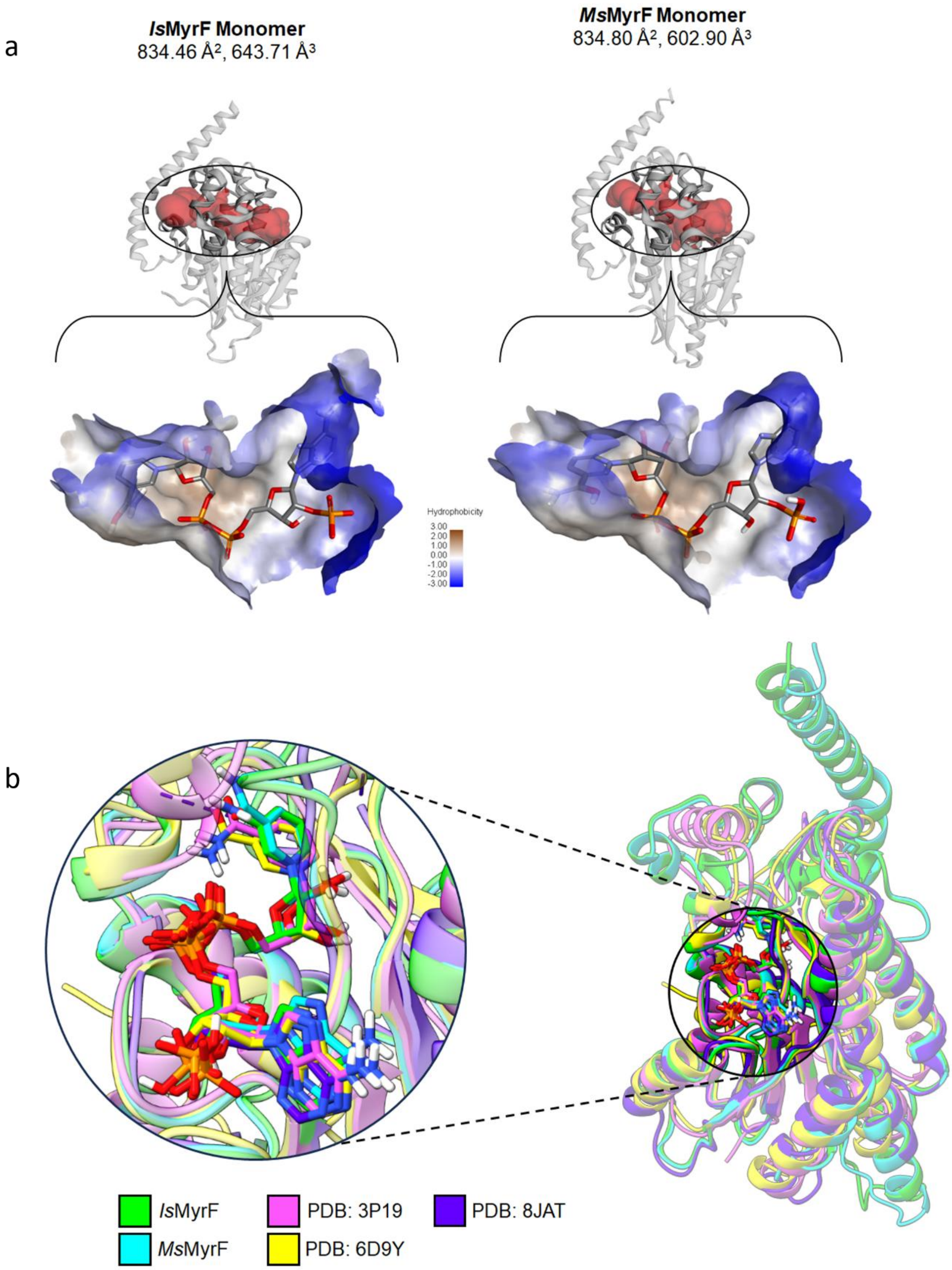

Fig. S10: Structural analysis of *Is*MyrF and *Ms*MyrF (myriocin ketodihydrosphingosine reductase (KDSR)). a. Identification of a binding pocket in MyrF by topological analysis (CASTp3.0). NADP+ was docked in the identified binding pockets (- 12.5 kcal mol^{-1}) and Kyte-Doolittle hydropathy surfaces were visualised in BIOVIA Discovery Studio 2020. b. Superimposition of homologous, NAD(P)-bound crystal structures over MyrF (with NADP^{+} docked).

a

```
MAAASFLASWIRGQKLRAPQMKDAPTFFRNLEEALDVRRVDHAMFTRTKSTWKTGDAIDF
CSNDLLSLGSTGQLRAAFLDELAQNPGFALYAGGSRLMDGNYDYIEQVEQEIADFHGAET
ALIVASGYEGNAAIYSAIPRPGDAIVYDELVHASTHDGMAHSLALTRAAFRHNDVDSFRD
TLTSVVDSQPMIRDGSRTIIVSVESVYSMDGDVCPLVELLEVAREVCPKGNTAFVVDEAH
ATGILGPNGAGLVNALGVENDIAIRLHTCGKALASTGAVILGNATVRTALMNFARSVIYT
TAPSFPSVAGIRAAYNLMRTGATKKPQDNIQHLVKYFFKTISSNPIWEKANDMGILSIPV
SDDWESRDFVAHIVPVWTRQRYNWWLVFHLQLSGISAFPIDYPTVPKGQSRIRVMFHAAN
TEADADFLAATICNWAKEMIDIEEGAEGKGKMPKAAQQVYALMAANAenlyfqgHHHHHH
```

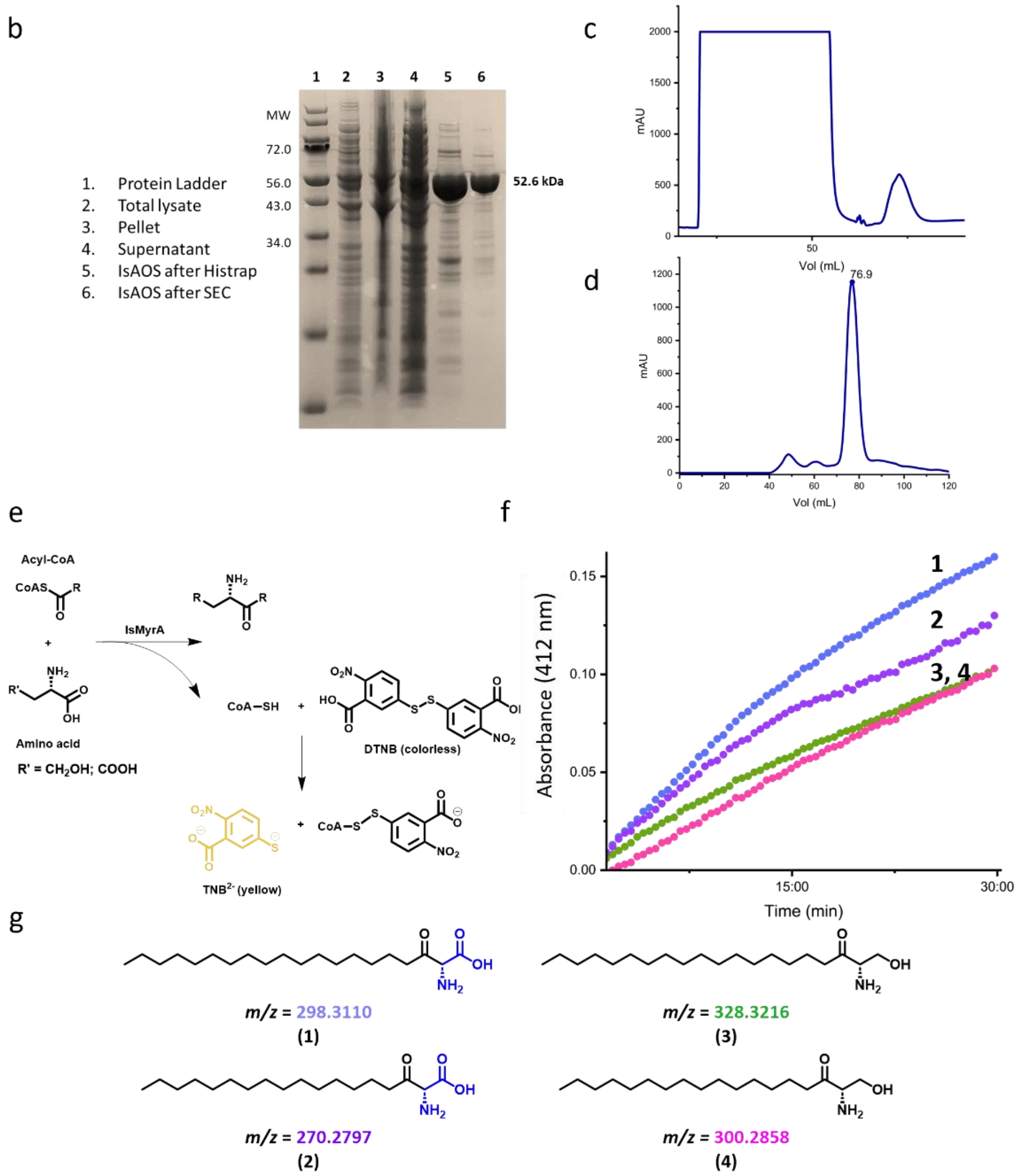

Fig. S11: IsMyrA protein purification and DTNB activity assay. a. Sequence of recombinant IsMyrA construct. b. SDS-PAGE gel of purification of IsMyrA. HiMark Pre-stained Protein Standard was used as ladder. Lanes 2 to 6 show each step of purification from transformed *E. coli* BL21 (DE3) 18+ hours post-induction using IPTG. See Fig. S14 for uncropped gel image. c. IsMyrA IMAC chromatogram. Cell-free extract was loaded using an AKTA start onto a 5 mL Nickel His-Trap FF column and eluted with a 10 – 300 mM imidazole gradient. Protein was detected at 280 nm. d. SEC purification of IsMyrA using a HiLoad 16/600 Superdex 200 pg (120 mL) SEC column, monitored at 280 nm. e. DTNB assay general reaction. The activity of IsMyrA was detected by capturing the CoASH released with 5,5′-dithiobis-(2-nitrobenzoic) acid (DTNB) and the spectrophotometric detection of the TNB^{2-} anion (412 nm). f. IsMyrA activity against acyl-CoAs (C_{16} and C_{18}) and amino acids aminomalonate and L-serine, detected using the DTNB assay. Raw data is available at https://doi.org/10.6084/m9.figshare.32652846. g. Molecular formula of the respective product of each reaction shown in (f).

Time [min]	A [%]	B [%]	Flow [mL/min]
**0.00**	70.	30	0.4
**1.00**	70	30	0.4
**8.00**	2	98	0.4
**9.50**	2	98	0.4
**9.60**	70	30	0.4
**10.00**	70	30	0.4

Figure S12: **Gradient steps for 30% acetonitrile LC-Method used for myriocin detection**.

A = MeCN. B = H_2O + 0.1% formic acid.

a

b

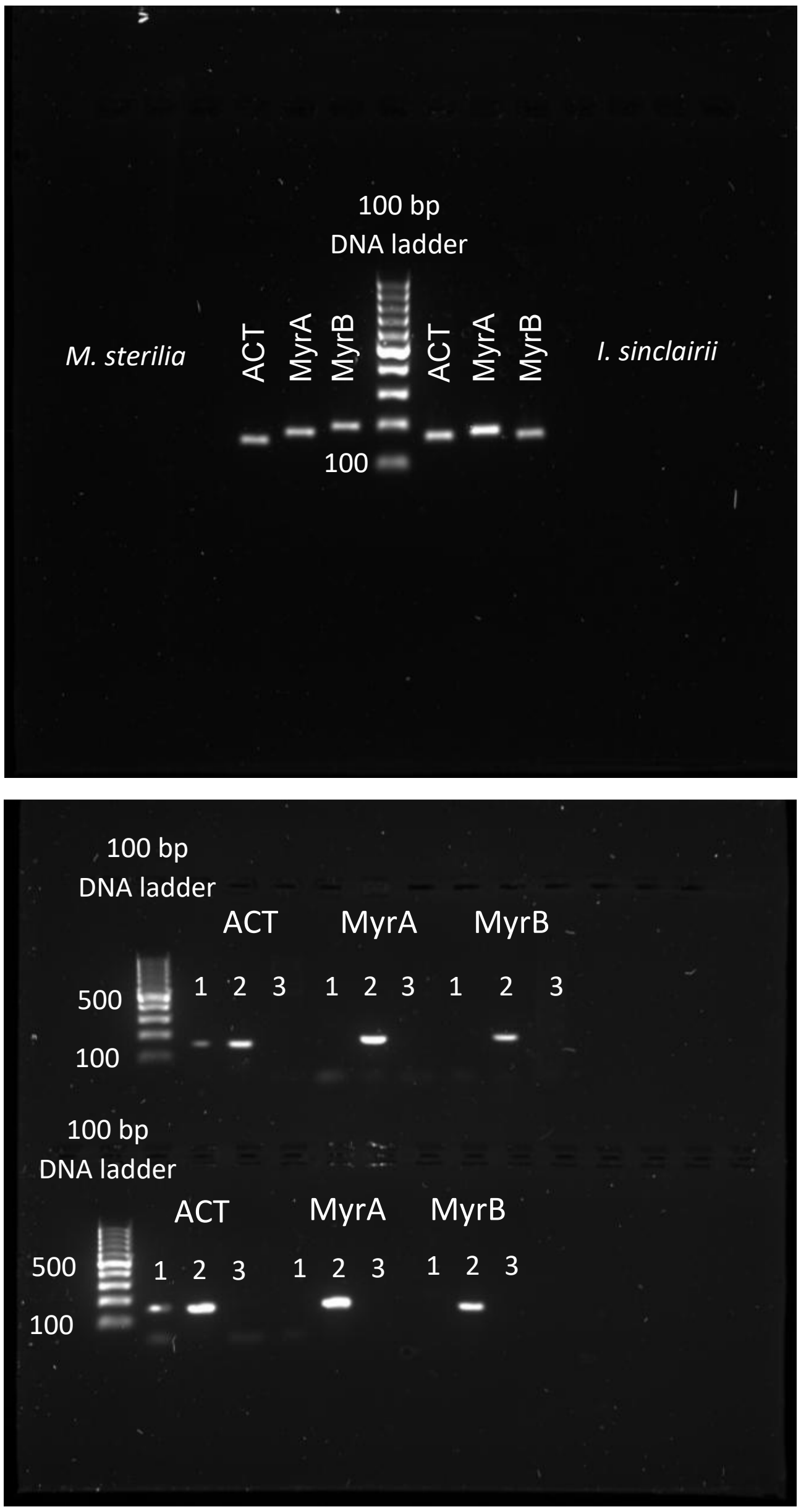

Fig. S13: Uncropped gel images of PCR and RT-PCR products shown in Fig. 4. a. PCR products from genomic DNA for actin *myrA* and *myrB*. Left: *M. sterilia*, right: *I. sinclairii.*
b. RT-PCR products for actin, *myrA* and *myrB* (1 = Day 10, 2 = Day 20, 3 = No nucleic acid control). Above: *M. sterilia*, below: *I. sinclairii.*

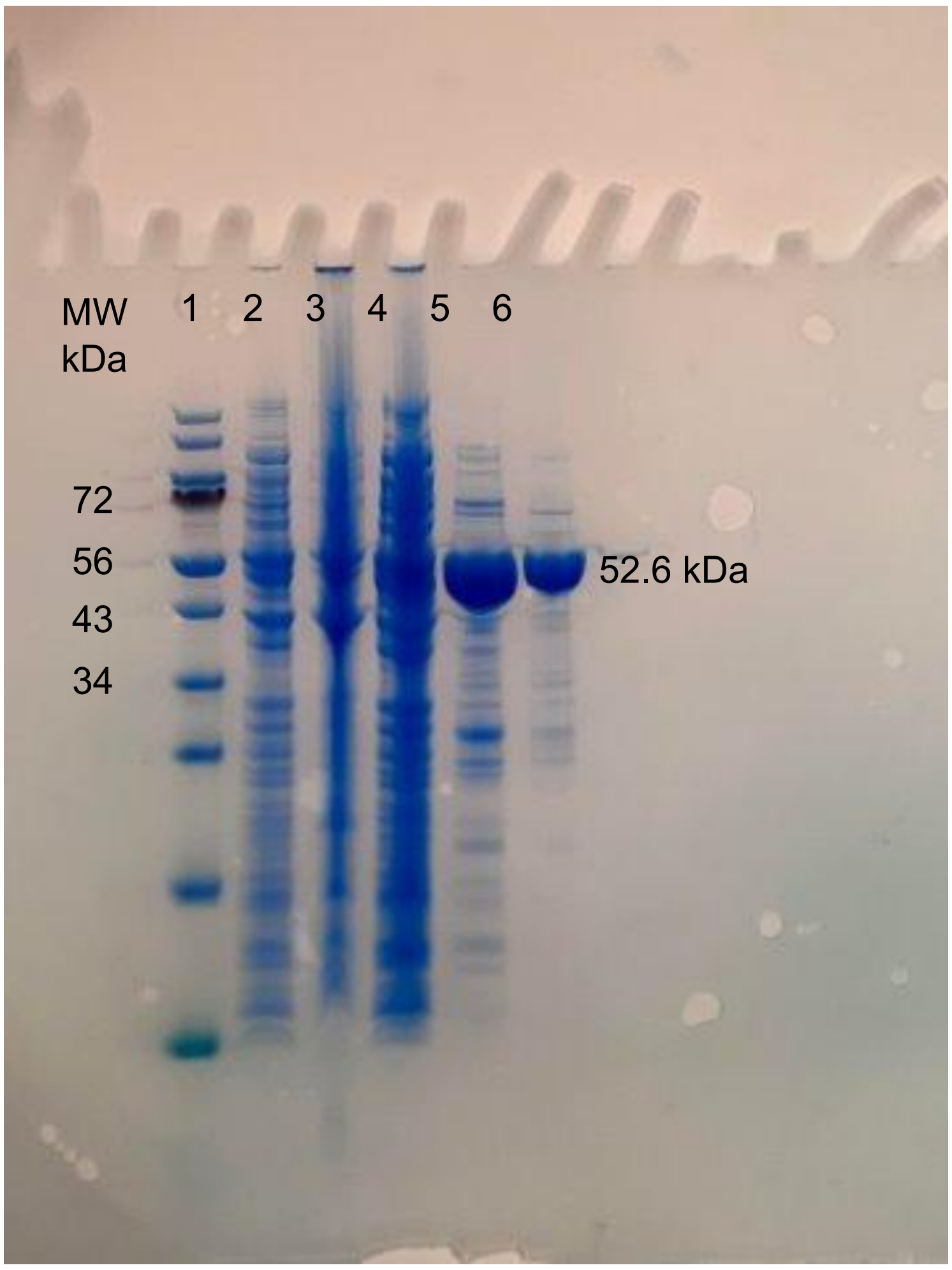

Fig. S14: Uncropped SDS-PAGE gel images of purified recombinant IsMyrA shown in Fig. 6 and Fig. S11.
Lanes (from left to right): 1. Protein molecular weight ladder (kDa); 2. Total lysate; 3. Pellet; 4. Supernatant; 5. IsAOS after Histrap; 6. IsAOS after SEC.
