# Supplementary material for: Evolution, structure and function of the putative biosynthetic gene cluster of the fungal secondary metabolite myriocin, a potent inhibitory sphingolipid
Source: Commun Biol. 2026 Jul 17;9:1249. doi: 10.1038/s42003-026-10708-9 (PMC13612406; doi:10.1038/s42003-026-10708-9)
Supplement: Supplementary file 3 [file 42003_2026_10708_MOESM3_ESM.pdf]

Description of Additional Supplementary Files

File name: Supplementary Data 1

Description: Quantification of myriocin production

File name: Supplementary Data 2

Description: Phylogenetic analysis of MyrB

File name: Supplementary Data 3A

Description: Raw data: Figure 6b

File name: Supplementary Data 3B

Description: Raw data: Figure 6d

File name: Supplementary Data 3C

Description: Raw data: Figure S11f
